# Review Article: The effectiveness of school‐based interventions for reducing screen time – a systematic review and meta‐analysis

**DOI:** 10.1111/camh.70022

**Published:** 2025-07-14

**Authors:** Nai Ming Lai, Yin Sear Lim, Nathorn Chaiyakunapruk, Shaun Wen Huey Lee, Tippawan Liabsuetrakul, Mohammad Sabbir, S.M. Hasan Mahmud, Umme Ruman Siddiqi, Tanvir Ahmed, Faheem U.L. Hasan, Pei Xuan Kuan

**Affiliations:** ^1^ School of Medicine Faculty of Health and Medical Sciences, Taylor's University Subang Jaya Malaysia; ^2^ Department of Pharmacotherapy University of Utah College of Pharmacy Salt Lake City UT USA; ^3^ School of Pharmacy Monash University Malaysia Bandar Sunway Malaysia; ^4^ Department of Epidemiology Faculty of Medicine, Prince of Songkla University Hat Yai Thailand; ^5^ Institute of Epidemiology Disease Control and Research Dhaka Bangladesh; ^6^ Directorate General of Health Services Dhaka Bangladesh; ^7^ Upazila Health and Family Planning Office Keraniganj Upazila Health Complex Dhaka Bangladesh; ^8^ Department of Medicine M Abdur Rahim Medical College Hospital Dinajpur Bangladesh; ^9^ Digital Health Research and Innovation Institute for Clinical Research, National Institutes of Health Setia Alam Malaysia

**Keywords:** Screen time, sedentary time, school, physical activity

## Abstract

**Background:**

Excessive screen time is associated with adverse physical and mental well‐being for children. Interventions to reduce screen time in different settings have been studied extensively, with mixed findings. We systematically reviewed evidence that evaluated the effects of screen time reduction interventions conducted in school settings on screen usage, physical, and mental health of school children.

**Methods:**

PubMed, Cochrane, PsycInfo, and Education Resources Information Centre (ERIC) (from inception till 12 September 2024) were searched for relevant randomised and cluster‐randomised trials. We assessed risk of bias using the Cochrane risk of bias 2 tool, performed Bayesian random‐effects meta‐analysis, and rated certainty of evidence using GRADE.

**Results:**

Thirty‐nine studies (95,033 participants), published between 1999 and 2024, were included. High risk of bias and great heterogeneity contributed to low‐certainty evidence for most outcomes. School‐based interventions modestly reduced screen time (SMD: −0.10, 95% CrI: −0.14, −0.06, 27 studies, *n* = 19,751, *I* ^2^: 85%) and increased physical activity (SMD: 0.10, 95% CrI: 0.02, 0.19, 21 studies, *n* = 14,944, *I* ^2^: 88%). No clear difference was observed in BMI (MD: −0.15, 95% CrI: −0.39, 0.03, 13 studies, *n* = 4683, *I* ^2^: 51%), although the subgroup of studies evaluating comprehensive lifestyle interventions appeared to show a slight BMI reduction, while studies evaluating screen‐time focused interventions showed no difference.

**Conclusions:**

School‐based interventions modestly reduce screen time and increase physical activity, but their effects on BMI are inconclusive. Variations in local school systems and cultural practices might have contributed to heterogeneity in study findings and should be considered in policy decisions. Future studies should strengthen the cluster‐randomization process and include academic performance as an outcome.

**Key Practitioner Messages**

**What is already known?**

- The harmful effects of excessive screen time on children have been studied extensively. Various interventions to reduce screen time have been evaluated, including school‐based interventions, with mixed findings of their effectiveness.

**What this study adds?**

- This systematic review and meta‐analysis of 39 studies (95,033 participants) shows that school‐based interventions slightly reduced screen time and increased physical activity among school children. The effect of school‐based interventions on BMI requires further evaluation.

**What is significant for clinical practice?**

- School‐based interventions may be considered in reducing screen time and increasing physical activity among school children. However, heterogeneity among study results suggests a need to consider local population characteristics, school system, and cultural factors in policy recommendations. Future research should improve the cluster‐randomization process and incorporate the results of academic performance.

## Introduction

Screen time refers to the time spent on electronic screens such as television, cell phones, computers, tablets, and games consoles (Barber et al., 2017). Easy access to portable media devices and the internet, with their appealing and addictive content, has led to a significant increase in screen time for educational, social, and entertainment purposes (Qi, Yan, & Yin, 2023). Reports showed that children aged 6–14 spend, on average, 3 hr daily, and older adolescents twice the amount on screen, with an increasing trend over the decade (Qi et al., 2023; Rideout, Foehr, & Roberts, 2010).

Screen time is mostly sedentary. Sedentary behaviour, defined as any waking behaviour with an energy expenditure of <1.5 metabolic equivalents (e.g., sitting, reclining or lying down) (WHO, 2017), has been identified as a risk factor for obesity and noncommunicable diseases (Katzmarzyk, Friedenreich, Shiroma, & I‐Min, 2022). With increased reliance on technology, advancement of transportation, and changes in cultural values following economic development (Labrique, Vasudevan, Kochi, Fabricant, & Mehl, 2013), many children and adults today lead a predominantly screen‐focused sedentary lifestyle (WHO, 2018).

Excessive screen time in children has been associated with problems such as dry eyes, weight gain, and psychological issues (Madigan, Browne, Racine, Mori, & Tough, 2019; Mineshita et al., 2021; Stiglic & Viner, 2019). Access to harmful content, particularly for children with limited self‐control, can exacerbate these problems, leading to addiction, poor social function, and self‐care (Beyens, Frison, & Eggermont, 2016; Rosen et al., 2013; Swider‐Cios, Vermeij, & Sitskoorn, 2023). A postulated mediator between screen time and cognitive, emotional, and behavioral problems is sleep disruption (Guerrero, Barnes, Chaput, & Tremblay, 2019; Li et al., 2019). The National Sleep Foundation's consensus statement highlights that screen use, particularly prebedtime content consumption, impairs sleep health in children and adolescents (Hartstein et al., 2024). While earlier research attributed sleep disruption primarily to blue light emission from screens (Cain & Gradisar, 2010), recent evidence suggests a more complex relationship involving screen content, timing of use, and sleep patterns (Bauducco et al., 2024). On the other hand, applications that involve sports and fitness management, active gaming movement, and healthy eating may offer a means to improve health (Iribarren et al., 2021; Middelweerd, Mollee, van der Wal, Brug, & te Velde, 2014). However, variable quality of the commercially available digital applications and their unsupervised use have raised concerns (Akbar, Coiera, & Magrabi, 2020; Sharp & O'Sullivan, 2017).

The use of digital devices in school has increased substantially in the past decade. Since the COVID‐19 pandemic, many schools officially integrate e‐Learning into their curricula (Ryan, Henderson, & Aagaard, 2021; Sonnenschein, Stites, Gursoy, & Khorsandian, 2023). A study showed that up to half of secondary school students use digital devices daily in school, and a significant proportion reported having eye strain (Ichhpujani, Singh, Foulsham, Thakur, & Lamba, 2019), gadget addiction (Jamir, Duggal, Nehra, Singh, & Grover, 2019) and academic deterioration (Jamir et al., 2019; Liza et al., 2023). The ongoing debate surrounding the use of digital devices in classrooms underscores the complexity of the issue, as striking a balance between students' rights, communicability and effective learning while minimizing physical and mental harm remains a challenge (“Debating the Use of Digital Devices in the Classroom,” 2012; Smale, Hutcheson, & Russo, 2021).

While guidelines recommend against excessive screen exposure (WHO, 2019), questions remain on the effectiveness of interventions aimed at reducing screen time. It has been reported that reduced screen time may not consistently translate to increased activity and health benefits (Fakhouri, Hughes, Brody, Kit, & Ogden, 2013; Patnode, Evans, Senger, Redmond, & Lin, 2017). Moreover, studies assessing such interventions varied widely in settings and strategies, with mixed findings (Jones et al., 2021; Wahi, Parkin, Beyene, Uleryk, & Birken, 2011). Suboptimal documentation of study methods and outcomes makes replication and application challenging (Wong, Bachman, Griggs, & Hartz, 2023). Improved clarity in the evidence is needed by regular synthesis of up‐to‐date, high‐level evidence on the effectiveness of screen‐time reduction interventions in different settings. Notably, understanding school‐based interventions is crucial due to the pivotal role schools play in children's learning, behavior, and health. To our knowledge, the last published meta‐analysis on school‐based interventions to reduce screen time was published in 2014 (Friedrich, Polet, Schuch, & Wagner, 2014). An updated synthesis of the evidence, as we have aimed to undertake here, is warranted following the publication of several new studies over the last decade.

## Methods

The review was registered in PROSPERO (CRD42022321753) and the Malaysian National Medical Research Register (ID‐23‐01760‐5QT). We conducted the review following Cochrane methods (Higgins et al., 2022) and reported following the PRISMA 2020 guidelines (Page et al., 2021) (Appendix S1). Additional details on our methods, including changes from the protocol, are described in Appendix S3.

### Inclusion criteria

We included RCTs, cluster RCTs and quasi‐randomized studies that enrolled students of primary, secondary or high schools. We excluded studies in preschool or kindergarten as these are covered by several published and ongoing reviews (Raj, Zulkefli, Minhat, & Ahmad, 2022). The studies should evaluate school‐based interventions that included a screen time reduction component, comparing with no intervention, or current standard educational program. Our prespecified primary outcomes included screen or sedentary time, academic performance and physical activity, measured using validated tools. Secondary outcomes included body mass index (BMI), mental health‐related outcomes such as internet addiction, self‐efficacy or perception of well‐being and incidence of anxiety or depression, level of knowledge, school attendance, incidence of bullying and user satisfaction from teachers or students.

### Search strategies

We searched MEDLINE (PubMed), Cochrane Central Register of Controlled Trials (CENTRAL) (which covered EMBASE, CINAHL, and trial registers including WHO International Trial Registry Platform and ClinicalTrials.gov), Educational Resources Information Centre (ERIC), and PsycInfo databases for published studies till September 12, 2024 (see Appendix S2 for search strategies) without language restriction. We searched the reference lists of relevant reviews for additional studies.

### Study selection and data extraction

Two pairs of authors (MSA, URS and SMHM, FUH) independently screened titles and abstracts for shortlisting using Rayyan (https://rayyan.ai/). Two authors (YSL and PXK) evaluated shortlisted articles in full text to determine eligibility. We used Robot Reviewer (https://www.robotreviewer.net/) as a test tool in extracting study characteristics including population, intervention, comparison, and outcomes. However, the output required extensive editing by two authors (YSL and PXK) as the extracted data were too lengthy and repetitive, often representing a verbatim transfer from the study papers. The edited data were transcribed into an Excel spreadsheet. We resolved disagreements by discussion leading to a consensus, with referral to the third author (NML) as required.

### Risk of bias assessment

Two authors (NML and PXK) independently assessed risk of bias using the Cochrane risk of bias 2 (RoB 2) tool (Higgins et al., 2022) with resources provided in the riskofbias.info website (https://www.riskofbias.info/). The tool consisted of five domains for RCTs (randomization process, deviation from intended interventions, missing outcome data, measurement of the outcome, selection of the reported results) and one additional domain for cluster RCTs (timing of identification or recruitment of participants). We assessed risk‐of‐bias based on the results of the three main outcomes: screen or sedentary time, physical activity, and BMI. We discussed all disagreements and achieved a consensus.

### Assessment of heterogeneity

We evaluated clinical, methodological, and statistical heterogeneity as recommended in the Cochrane Handbook (Higgins et al., 2022). To evaluate clinical heterogeneity, we assessed major differences in participants' age, sex, and school setting, category of intervention delivered (namely, screen‐time‐focused or comprehensive lifestyle intervention) and outcome measurements. For methodological heterogeneity, we assessed differences in risk of bias. For statistical heterogeneity, we used *I* ^2^ statistic with a cutoff of 50% to indicate substantial heterogeneity (Higgins et al., 2022). If substantial heterogeneity was found, we explored possible explanations in study characteristics in terms of population, intervention, comparison, and outcome measurement (as detailed in Appendices S3 and S5).

### Assessment of missing data, publication, and reporting biases

If we found a significant dropout rate (>20%), we would judge the study at high risk of bias in terms of missing outcome data. We did not contact any author to request further information, as we did not consider the missing data to be critical for meta‐analysis.

We created funnel plots to screen for publication bias for outcomes in which there are >10 studies using JASP software version 0.18.3 (Team, 2023) (https://jasp‐stats.org/). If the funnel plot shows significant asymmetry, we would downgrade the certainty of evidence based on publication bias (Sterne, Egger, Moher, & Boutron, 2017).

We did not assess the risk of bias that might have arisen from expected but missing results in a synthesis (i.e. reporting biases), using the recently established risk of bias tool such as the RoB‐ME tool (https://methods.cochrane.org/bias/resources/rob‐me) (Page et al., 2023).

### Data synthesis

We tabulated major components of the intervention and comparison in each study in the characteristics of included studies table (Table S1). Although we categorised the intervention into two major subgroups (i. screen‐time focused intervention and ii. comprehensive lifestyle intervention with screen time reduction components), we considered that their distinction not sufficiently clear cut to warrant separate comparisons. Consequently, we grouped all studies with relevant data under one comparison, and only assessed the nature of intervention (whether predominantly screen‐time focused or a comprehensive lifestyle intervention) via subgrouping as part of our assessment of heterogeneity.

There were instances when we exercised our judgment in selecting outcomes for our meta‐analysis, among multiple similar outcomes reported in a study. For example, for screen or sedentary time, TV viewing and video gaming time were reported without reporting total screen time (Robinson & Borzekowski, 2006) and weekday or weekend media use was reported separately (Andrade et al., 2015; Robinson & Borzekowski, 2006). In the former instance, we selected TV viewing based on the consideration that TV viewing might still be more common than video gaming globally. In the latter instance, we chose the data for weekends based on consistent reports showing children's propensity to have greater screen time during weekends (Esposito et al., 2022; Liangruenrom, Dumuid, & Pedisic, 2023; Sigmundová et al., 2016; Sigmundová, Badura, Sigmund, & Bucksch, 2018; Sigmundová & Sigmund, 2021). Our main consideration was to choose one component outcome that would best represent typical screen time. Among studies that reported different intensities of physical activity (e.g., any physical activity, moderate to vigorous physical activity) without reporting total physical activity (Gortmaker et al., 1999; Harrison, Burns, McGuinness, Heslin, & Murphy, 2006; Jones, Hoelscher, Kelder, Hergenroeder, & Sharma, 2008; Lubans et al., 2013; Peralta, Jones, & Okely, 2009; Salmon, Ball, Hume, Booth, & Crawford, 2008; Smith et al., 2014; Verswijveren et al., 2022), we chose physical activity of the highest intensity as we considered it to be more likely to be differentiating. One study had more than two interventions evaluated (Verswijveren et al., 2022). In this case, we extracted data from the group with the most comprehensive and intensive intervention against the control group.

We performed Bayesian random effects meta‐analysis using neutral priors assigned to model parameters. Prior distributions for effect sizes were determined using a Cauchy distribution with a mean of 0 and a scale parameter of 0.707, which corresponded to a moderately wide variance of 0.5, as recommended by Gronau, Heck, Berkhout, Haaf, and Wagenmakers (2021). To estimate between‐study variation of effect sizes (tau), we chose a wide and weakly informative prior using the inverse gamma distribution with a shape of 1 and a scale of 0.15, as recommended by Berkhout, Haaf, Gronau, Heck, and Wagenmakers (2022). We used Markov Chain Monte Carlo (MCMC) simulation with 3000 iterations to allow convergence in producing the analysis output.

We derived the effect sizes of each study with their corresponding SEs using the generic inverse variance method via RevMan 5.4 (“Review Manager 5 (RevMan 5),” 2020). For continuous outcomes, we used mean difference (MD) or standardized mean difference (SMD) depending on whether the outcomes were measured using the same or different scales among studies. As the included studies reported their outcome data using different scales for all outcomes except for BMI, we pooled the data using SMD in almost all analyses. We categorized SMD according to Cohen et al., with SMD of <0.2 as small, 0.2–0.5 as medium, >0.5–0.8 as medium to large, and >0.8 as large effect size (Cohen, 1988). For dichotomous outcomes, we entered the effect size data from each study in the form of logOR and SE, derived using RevMan 5.4 (“Review Manager 5 (RevMan 5),” 2020). We then exponentiated the synthesized pooled estimates (logOR) to OR in our report. We presented all points estimates with their 95% credible intervals (CrI). Unlike traditional confidence intervals, credible intervals have a more intuitive interpretation: there is a 95% probability that the true effect lies within the interval, given the observed data (Hespanhol, Vallio, Costa, & Saragiotto, 2019).

We derived missing SE by multiplying the SD with the square root of the sample size in the corresponding group or by dividing the distance between the two ends of the 95% CI by 3.92. We used adjusted effect sizes of the individual articles if these were reported.

All meta‐analyses were conducted using the R package metaBMA (Heck, Gronau, Wagenmakers, & Indrajeet, 2019) via JASP software version 0.17.3 (Team, 2023) (https://jasp‐stats.org/).

### Certainty of evidence rating

Two authors (NML and PXK) independently assessed the certainty of evidence for three main outcomes, namely, screen or sedentary time, physical activity, and BMI using the GRADE approach (Schünemann, Brożek, Guyatt, & Oxman, 2013). We considered evidence from RCTs as high certainty to begin with, downgrading one level for serious (or two levels for very serious) limitations based upon five considerations: risk of bias, inconsistency across studies, indirectness of the evidence, imprecision of estimates, and publication bias (see Appendix S3 for details). We used GRADEpro GDT (https://www.gradepro.org/) to create a summary of findings table.

## Results

**Figure 1:**
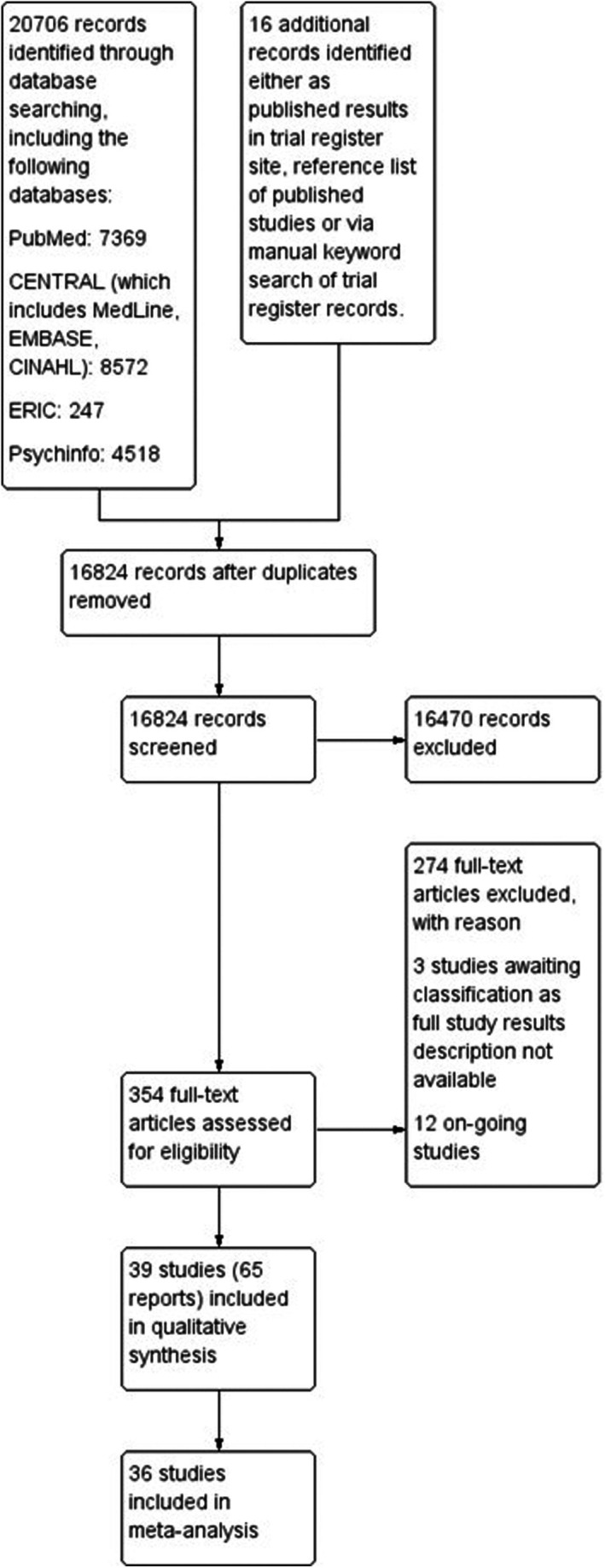
PRISMA flow diagram

Our searches yielded 20,706 records (16,824 after de‐duplication). We shortlisted 354 and included 65 reports that described 39 distinct studies (see Figure 1 for the PRISMA flow diagram). There are 12 recently registered studies with no results available; hence, they were classified as on‐going studies. Thirteen included studies had multiple associated published papers, including two with six (Lubans et al., 2013; Verswijveren et al., 2022), one with four (Robinson, Wilde, Navracruz, Haydel, & Varady, 2001), three with three (Champion et al., 2023; Lindenberg, Kindt, & Szász‐Janocha, 2022; Smith et al., 2014), and seven with two published papers (Aittasalo et al., 2019; Babic et al., 2016; Barbosa Filho et al., 2019; Bergh et al., 2014; Gortmaker et al., 1999; Robinson & Borzekowski, 2006; Salmon et al., 2008).

### Included studies

The characteristics of included studies are summarized as follows (see Table S1 for details).

### Design and setting

Among the 39 studies (*n* = 95,033), 33 were cluster‐RCTs, two were quasi‐randomized trials, and four were individual RCTs conducted within a single or multiple schools (Agbaria, 2023; Ji & Wong, 2023; Peralta et al., 2009; Schmidt et al., 2022). These studies were published between 1999 and 2023 in 18 countries, including the USA (*n* = 9), Australia (*n* = 7), Germany (*n* = 4), Brazil, China, Iran, the Netherlands (*n* = 2), and Bangladesh, Belgium, Ecuador, England, Finland, Ireland, Israel, Italy, Malaysia, Norway, and Spain (*n* = 1). The median participant number was 422 (Lindenberg et al., 2022) (range: 33 (Peralta et al., 2009) to 58, 474 (Wang et al., 2022)).

### Population

Most studies enrolled children and adolescents aged 8–16 years from diverse school communities. Three studies recruited older participants from vocational schools (mean age: 19–20) (Ji & Wong, 2023; Pietsch et al., 2023; Schmidt et al., 2022); one study recruited children in the first grade (aged 7–8) (Brandstetter et al., 2012) and two recruited children from a mixture of preschool and primary schools (Cong, Feng, Liu, & Esperat, 2012; De Coen et al., 2012). Two studies (Peralta et al., 2009; Smith et al., 2014) enrolled exclusively male participants and three (Chavarro, Peterson, Sobol, Wiecha, & Gortmaker, 2005; Jones et al., 2008; Lubans et al., 2013) exclusively female participants. Six studies explicitly stated that participants were recruited from lower‐income communities (De Coen et al., 2012; Harrison et al., 2006; Lubans et al., 2013; Salmon et al., 2008; Smith et al., 2014; Wright, Giger, Norris, & Suro, 2013). Seven studies recruited participants based on risk factors, such as overweight or obesity (Amini et al., 2015; Bagherniya et al., 2018; Mohammed Nawi & Che Jamaludin, 2015), high risk for gaming or internet addiction (Ji & Wong, 2023; Lindenberg et al., 2022; Schmidt et al., 2022), and low cardiovascular fitness scores (Peralta et al., 2009).

### Intervention and comparison

We identified two broad categories of interventions:

- Intervention focused on screen time reduction (14 studies): studies mainly employing health education integrated into the school curriculum (Babic et al., 2016; Dos Santos, Salmon, Arundell, Lopes, & Silva, 2021; Harrison et al., 2006), substitution of screen time with physical activity and behavioral contracts (Andrade et al., 2015; Bagherniya et al., 2018; Bickham, Hswen, Slaby, & Rich, 2018; Brandstetter et al., 2012), a combination of health education and behavioral contracts (Robinson et al., 2001; Robinson & Borzekowski, 2006; Smith et al., 2014), and cognitive‐behavioral therapy or counseling targeting internet or gaming addiction (Agbaria, 2023; Ji & Wong, 2023; Lindenberg et al., 2022; Schmidt et al., 2022). Five studies involved parental participation (Babic et al., 2016; Bagherniya et al., 2018; Brandstetter et al., 2012; Robinson & Borzekowski, 2006; Smith et al., 2014).
- Comprehensive lifestyle interventions with a screen time component (25 studies): studies mainly employing health education on healthy lifestyles integrated into the curriculum (Aittasalo et al., 2019; Barbosa Filho et al., 2019; Champion et al., 2023; Gortmaker, Cheung, et al., 1999; Gortmaker, Peterson, et al., 1999; Mohammed Nawi & Che Jamaludin, 2015; Wang et al., 2022) with behavior modification focusing on dietary adjustments and physical activity (Aceves‐Martins et al., 2022; Ahmed, Kolbe‐Alexander, & Khan, 2022; Amini et al., 2015; Bergh et al., 2014; Champion et al., 2023; Chavarro et al., 2005; Chinapaw, Singh, Brug, & van Mechelen, 2008; Cong et al., 2012; De Coen et al., 2012; Jones et al., 2008; Lubans et al., 2013; Peralta et al., 2009; Salmon et al., 2008; Wang et al., 2022; Wright et al., 2013). Some studies included telephone or app‐based monitoring (Centis et al., 2012; Champion et al., 2023; Pietsch et al., 2023) and homework assignments (Kocken et al., 2016; Lawlor et al., 2016). Eight studies involved parental participation (Amini et al., 2015; Bergh et al., 2014; Centis et al., 2012; Cong et al., 2012; Kocken et al., 2016; Lawlor et al., 2016; Lubans et al., 2013; Wright et al., 2013).

In both categories, intervention was reinforced through seminars, workshops, e‐health messaging, newsletters, banners, social media, smartphone applications, websites, and modifications to the school's food programs. Implementation was typically carried out by the research team in collaboration with teachers, sports services, or physical education specialists and nutritionists, with two studies involving trained psychologists (Lindenberg et al., 2022; Schmidt et al., 2022). Duration of interventions varied widely from 2 hr (Peralta et al., 2009) to two academic years (Andrade et al., 2015; Bergh et al., 2014; Champion et al., 2023; Cong et al., 2012; De Coen et al., 2012; Gortmaker, Cheung, et al., 1999; Gortmaker, Peterson, et al., 1999; Jones et al., 2008; Kocken et al., 2016; Lawlor et al., 2016).

Most studies either did not clearly describe the interventions received in the control group or reported the group as having received standard curriculum. Where stated, control group participants either had equal access to sport equipment or materials (Gortmaker, Cheung, et al., 1999; Mohammed Nawi & Che Jamaludin, 2015), received intervention following study completion (Chavarro et al., 2005; Gortmaker, Cheung, et al., 1999; Lubans et al., 2013; Pietsch et al., 2023; Smith et al., 2014), or were engaged in “weekly classroom conversation” (Agbaria, 2023).

### Outcomes assessment

Twenty‐three studies (Ahmed et al., 2022; Aittasalo et al., 2019; Andrade et al., 2015; Babic et al., 2016; Bagherniya et al., 2018; Bergh et al., 2014; Bickham et al., 2018; Champion et al., 2023; Chavarro et al., 2005; Chinapaw et al., 2008; De Coen et al., 2012; Dos Santos et al., 2021; Gortmaker, Cheung, et al., 1999; Gortmaker, Peterson, et al., 1999; Harrison et al., 2006; Jones et al., 2008; Kocken et al., 2016; Pietsch et al., 2023; Robinson & Borzekowski, 2006; Salmon et al., 2008; Smith et al., 2014; Verswijveren et al., 2022; Wright et al., 2013) reported screen or sedentary time as the primary outcome and four (Centis et al., 2012; Lawlor et al., 2016; Lubans et al., 2013; Peralta et al., 2009) as a secondary outcome. Physical activity was the primary outcome in 15 studies (Ahmed et al., 2022; Aittasalo et al., 2019; Bergh et al., 2014; Champion et al., 2023; Chavarro et al., 2005; Chinapaw et al., 2008; De Coen et al., 2012; Gortmaker, Cheung, et al., 1999; Harrison et al., 2006; Jones et al., 2008; Kocken et al., 2016; Salmon et al., 2008; Smith et al., 2014; Verswijveren et al., 2022; Wright et al., 2013) and the secondary outcome in five studies (Bagherniya et al., 2018; Centis et al., 2012; Gortmaker, Peterson, et al., 1999; Lubans et al., 2013; Peralta et al., 2009).

Additionally, BMI, mental health‐related outcomes such as internet addiction and self‐efficacy were reported, while no studies reported academic performance, bullying, attendance, and user satisfaction. Other outcomes reported included consumption of fruit, vegetables, and high‐energy foods or drinks and aggressive behavior. However, these were not our prespecified outcomes; hence, they were not included in our meta‐analysis.

### Funding

Funding sources, where reported, included government agencies (18 studies) and nongovernmental agencies (five studies). Three studies reported as not funded (see Table S1 for details).

### Excluded studies

We excluded 274 reports for various reasons (see Figure 1 for reasons of exclusion and online supplement Appendix S4 for a full citation).

### Risk of bias assessment

**Figure 2:**
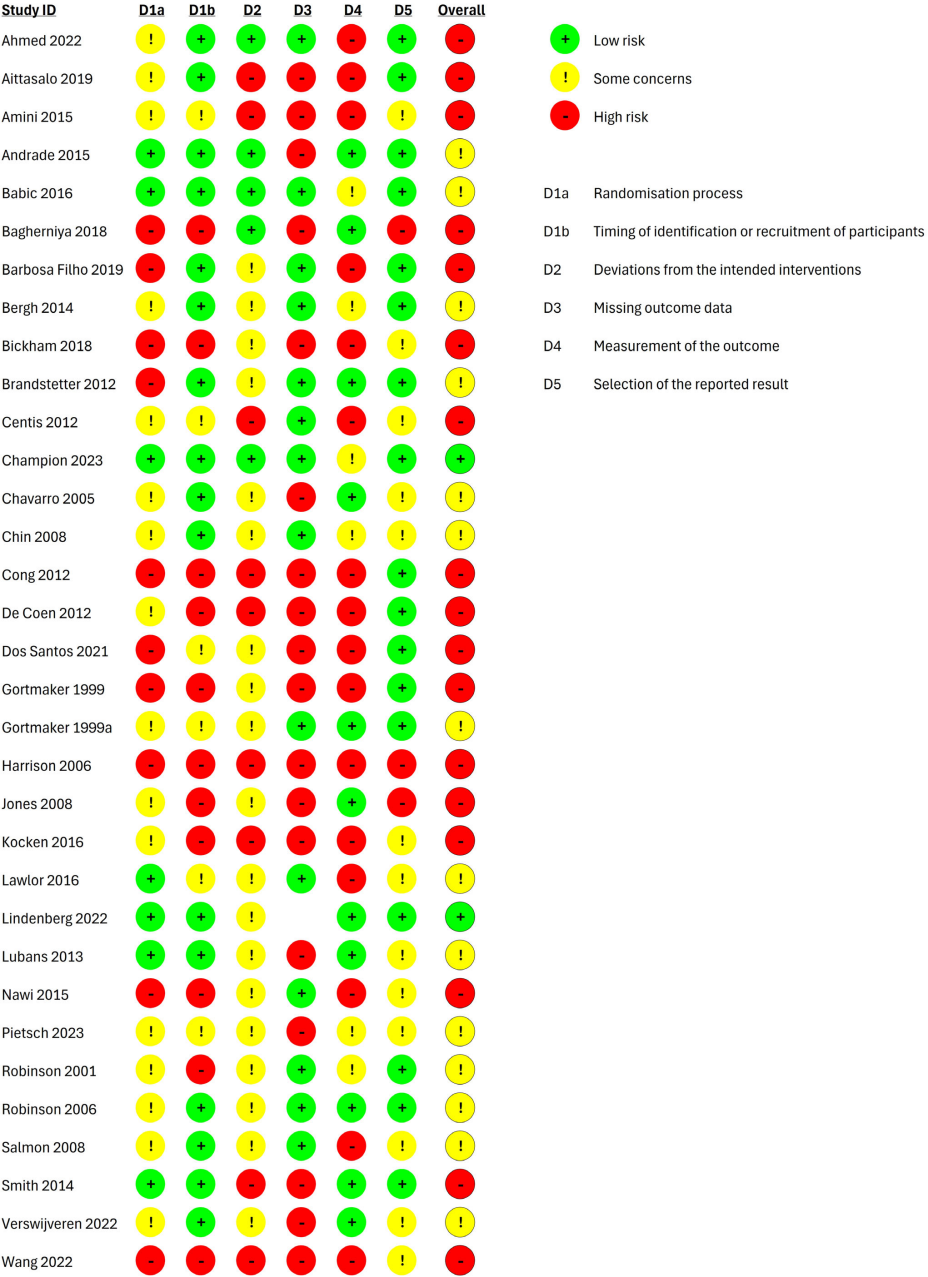
Risk‐of‐bias graph for cluster‐RCTs

Among cluster‐RCTs and quasi‐randomized cluster trials, two (Champion et al., 2023; Lindenberg et al., 2022) were considered to have an overall low risk of bias, 19 were judged to have a high risk of bias, and 14 were judged as having some concerns with regards to the results of screen and sedentary time, physical activity, and BMI. There were concerns in most studies in the following domains that they might lead to bias in the results: randomization process, timing of identification or recruitment of participants, deviation from the intended interventions, missing outcome data, and measurement of the outcome (Figure 2).

**Figure 3:**
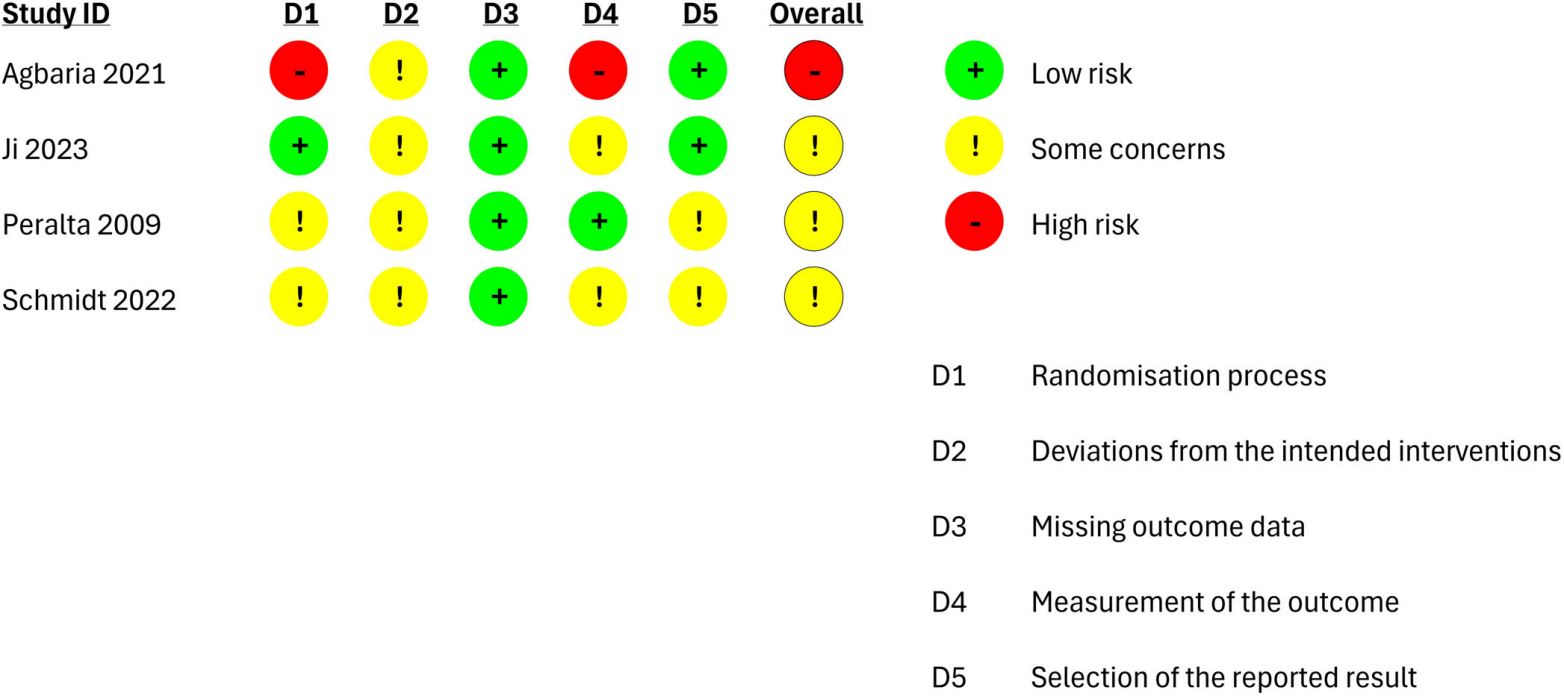
Risk‐of‐bias graph for individual RCTs

Among the four individual RCTs, one (Agbaria, 2023) was judged to have an overall high risk of bias and three (Ji & Wong, 2023; Peralta et al., 2009; Schmidt et al., 2022) as having some concerns with regards to the results of the aforementioned outcomes. In Agbaria 2022 (Agbaria, 2023), there were major concerns in the randomization process and measurement of the outcome, whereas in the remaining three studies (Ji & Wong, 2023; Peralta et al., 2009; Schmidt et al., 2022) there were concerns in the randomization process, deviation from the intended interventions, measurement of the outcome, and selection of the reported results (Figure 3).

### Effect estimates

Overall, 36 studies (*n* = 84,694) contributed suitable data for meta‐analysis. Screen or sedentary time and physical activity were measured differently across studies, employing various metrics such as time duration (in minutes or hours) or frequency (number of blocks per day or week). These estimates were pooled using SMD.

As the estimates of most outcomes show substantial heterogeneity, we detailed our exploration of heterogeneity in Appendix S5 and certainty‐of‐evidence ratings for major outcomes in Table S2. Following is a summary of the results.

### Screen or sedentary time

**Figure 4:**
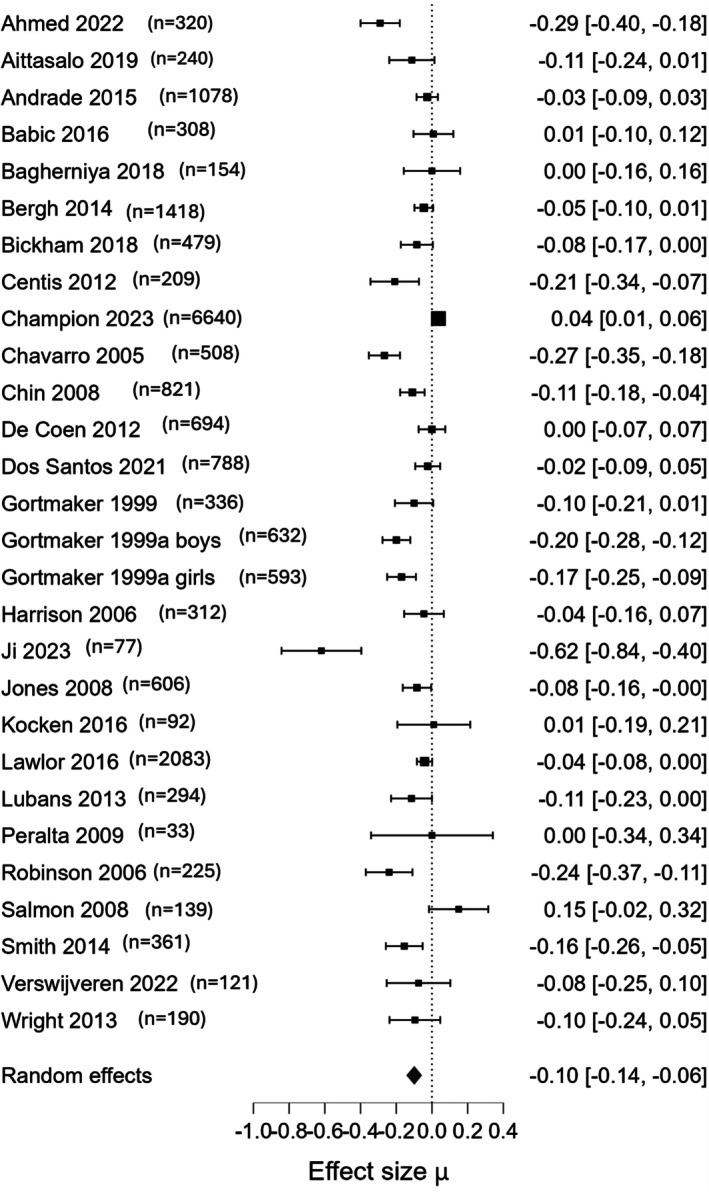
Forest plot for the main outcome: amount of screen time or sedentary (effect measure: SMD)

Based on 27 studies (*n* = 19,751), screen time reduction intervention may slightly reduce screen or sedentary time (SMD: −0.10, 95% CrI: −0.14, −0.06, low‐certainty evidence) (Figure 4). Funnel plot does not indicate publication bias (Figure S1).

Based on four studies (*n* = 9501), we are uncertain whether there is any difference in the likelihood of attaining screen or sedentary time expectations (OR: 0.90, 95% CrI: 0.45, 1.70, *I* ^2^ 89%, very‐low‐certainty evidence) (Figure S2).

### Physical activity

**Figure 5:**
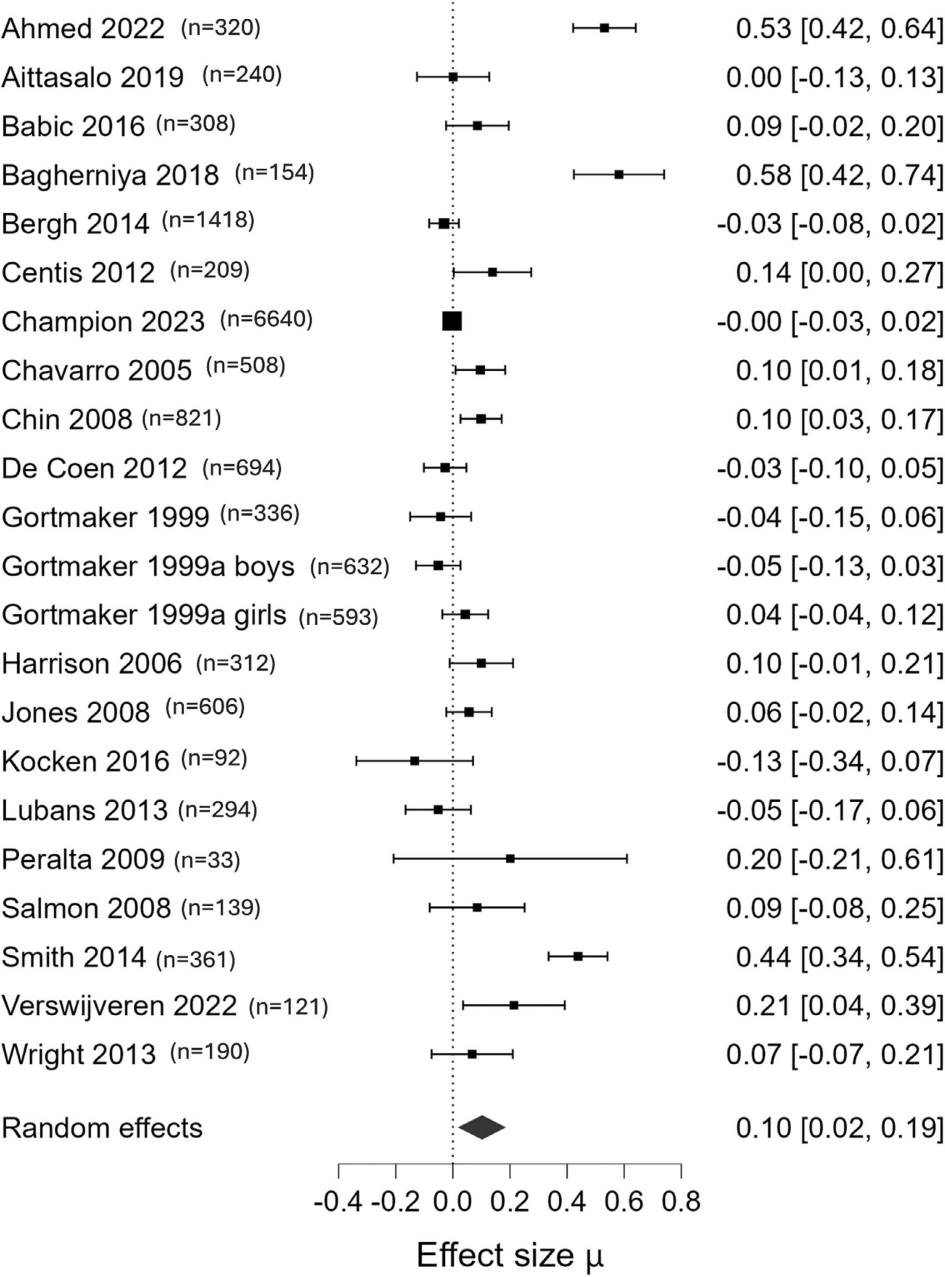
Forest plot for the main outcome: amount of physical activity (effect measure: SMD)

Based on 21 studies (*n* = 14,944), screen time reduction intervention may lead to a slight increase in physical activity (SMD: 0.10, 95% CrI: 0.02, 0.19, *I* ^2^ 88%, low‐certainty evidence) (Figure 5). Funnel plot does not indicate publication bias (Figure S3).

Based on four studies (*n* = 10,988), we are uncertain whether there is any difference in the likelihood of attaining physical activity expectations (OR: 1.43, 95% CrI: 0.90, 2.27, *I* ^2^ 92%) (Figure S4).

### Body mass index

Based on 13 studies (*n* = 4683), there may be little to no difference in BMI between groups (MD: −0.15, 95% CrI: −0.39, 0.03, *I* ^2^ 51%, low‐certainty evidence) (Figure S5a). In our exploration of heterogeneity, we found a significant difference between the pooled subgroup estimates of screen‐time focused interventions (MD −0.1, 95% CrI: −0.17, 0.23; five studies, *n* = 2112) (Figure S5b) and comprehensive lifestyle interventions (MD −0.35, 95% CrI −0.78, −0.05; eight studies, *n* = 2571) (Figure S5c), suggesting that there is a modest reduction in BMI with lifestyle intervention but not with screen‐time focused intervention. The funnel plot does not indicate publication bias (Figure S6).

Based on two studies (*n* = 1995) including one with separate results for girls and boys (Gortmaker, Peterson, et al., 1999), there is little or no difference in the number of participants with obesity (OR: 0.76, 95% CrI: 0.48, 1.20, *I* ^2^: 16.6%) (Figure S7).

### Mental health‐related outcomes

It is uncertain whether there are differences in the severity of gaming or internet addiction (SMD: −0.41, 95% CrI: −0.85, 0.10, four studies, *n* = 1156, *I* ^2^ 97%) (Figure S8) and self‐efficacy or well‐being (SMD: 0.14, 95% CrI: −0.07, 0.34, 10 studies, *n* = 5850, *I* ^2^ 97%) (Figure S9).

### Level of knowledge

Two studies (Champion et al., 2023; Wang et al., 2022) reported knowledge in different manners, which precluded meta‐analysis. Champion et al. (2023) (*n* = 6640) reported that students who received the healthy lifestyle interventions scored higher on questions related to high‐risk health behaviors (MD 0.51, 95% CI: 0.34, 0.68), while Wang et al. (2022) (*n* = 58,474) reported an increase in the number of participants who correctly answered a question on exercise (OR 1.19, 95% CI: 1.07, 1.33).

Other prespecified outcomes in our protocol that were not assessed in the included studies: awareness of the adverse effects of screen time, school absentees/attendance, and incidence of bullying.

## Discussion

Based on mostly low‐certainty evidence, school‐based interventions may slightly reduce screen or sedentary time and increase physical activity. However, the effects varied among studies and were not adequately explained by whether screen time reduction was the main intervention component or a part of a comprehensive lifestyle interventions, except that a slight reduction in BMI appeared to be achieved with comprehensive lifestyle interventions but not with screen‐time‐focused interventions. The difference in the subgroup effects on BMI is not definitive and requires confirmation by well‐designed head‐to‐head trials between the two intervention approaches. Evidence is insufficient for the other outcomes such as academic performance, internet addiction, and self‐efficacy. Besides heterogeneity, the evidence is affected by the risk of bias of the studies and imprecision, as most secondary outcomes were contributed by very few studies.

There are at least 12 published systematic reviews on screen time reduction interventions for children (Friedrich et al., 2014; Jones et al., 2021; Krafft et al., 2023; Liu et al., 2019; Maniccia, Davison, Marshall, Manganello, & Dennison, 2011; Marsh, Foley, Wilks, & Maddison, 2014; Martin, 2020; Raj et al., 2022; Schmidt et al., 2012; Wahi et al., 2011; Wu, Sun, He, & Jiang, 2016; Zhang et al., 2022). Most reviews show substantial heterogeneity in line with the current review, with slight reductions in screen time and mixed findings on BMI. Among them, only Friedrich et al. (2014) focused on school‐based interventions (Friedrich et al., 2014). Based on 16 RCTs involving 8785 participants, Friedrich et al. showed that school‐based interventions led to a small but significant reduction in screen time (SMD of −0.25). The current updated meta‐analysis, with data from 36 studies and over 84,000 participants, revealed a fuller but a more nuanced picture: we reported a smaller reduction in screen time, a slight increase in physical activity, and a slight reduction in BMI in the subgroup that received comprehensive lifestyle interventions.

We performed comprehensive searches and gathered a globally representative sample of studies. Utilizing a Bayesian approach, we offer an alternative to conventional frequentist meta‐analysis with the flexibility of incorporating various degrees of individual prior beliefs about the effects. The strengths of the Bayesian approach include the incorporation of prior information or beliefs, which may be useful when data are limited and may reflect a more realistic process in synthesizing and updating evidence (Sutton & Abrams, 2001). The Bayesian approach also enables a more intuitive interpretation of the plausible range of estimates via the credible interval (Hackenberger, 2020; Higgins, Thompson, & Spiegelhalter, 2009).

We acknowledge the following limitations. The high degree of heterogeneity in our analyses remained inadequately explained, likely stemming from fine details in population characteristics, intervention design, and delivery beyond the crude subgrouping of screen‐time focused versus comprehensive lifestyle interventions as we have undertaken here as well as comparisons and outcome measurements. More in‐depth assessments of these factors are required, including, among others, adherence to interventions and the accuracy of self‐reported outcome measures. However, these details are often lacking in published reports. We included two quasi‐RCTs with an overall high risk of bias due to concerns in allocation methods. However, we did not consider these two quasi‐RCTs to have major influence on the relevant effect estimates because of the large number of included studies and the finding that 20 of these studies were judged to have an overall high risk of bias. The next limitation pertains to the use of BMI as an outcome measure. Although widely reported, BMI alone does not accurately measure body adiposity and serve as a marker of health status (Nuttall, 2015; Rothman, 2008). Despite the aforementioned advantages of the Bayesian approach, the choice of priors may be subjective and varies among researchers, and this may affect the reproducibility of the estimates (Sutton & Abrams, 2001). Next, the current review might not have captured a complete set of relevant studies. Despite a careful assessment of a broad set of 340 studies that appeared relevant, we might have missed eligible studies, for instance, studies that evaluated interventions predominantly implemented outside the school setting or not clearly labeled as school‐based but contained important components in the school. Next, in dealing with multiple reports, we performed careful assessments and merged the reports based on the reported study period, participant characteristics, and settings. Despite our careful assessment, there is a possibility of inappropriate merger of studies.

## Conclusions

School‐based interventions demonstrate modest effectiveness in reducing screen time and increasing physical activity among students. However, the substantial variation in study findings prevents definitive practice recommendations. Head‐to‐head trials between screen‐time focused versus comprehensive lifestyle intervention approaches are required to determine their effect on BMI. While differences in study results are to be expected given the variations in cultural settings, population characteristics, school systems, and intervention approaches, an in‐depth evaluation of these factors in the local context could provide a useful guidance to policymakers and researchers. Future research should focus on improving the randomization process, recruitment strategies in the context of cluster‐RCTs, monitoring and improving participant adherence, as well as standardizing outcome measurement.

## Author contributions

N.M.L. conceptualized the review, drafted the protocol including methodology, performed risk‐of‐bias assessment, curated the data for meta‐analysis and certainty‐of‐evidence rating, drafted the initial manuscript, and critically reviewed and revised the manuscript. M.S., S.M.H.M., U.R.S., T.A., and F.U.L.H. drafted the protocol, including methodology, performed the search, screened and selected studies, checked data accuracy, curated the data for meta‐analysis, critically reviewed and revised the manuscript. Y.S.L. performed data extraction, checked data accuracy, critically reviewed, and revised the manuscript. P.X.K. performed data extraction, checked data accuracy, performed risk‐of‐bias assessment and certainty‐of‐evidence rating, critically reviewed, and revised the manuscript. N.C., S.W.H.L., and T.L. critically reviewed and revised the manuscript.

## Funding information

There is no direct funding received for the development of this systematic review and meta‐analysis. The authors were involved in the Child Health and Mortality Prevention Surveillance Network (CHAMPS) initiative, from Emory Global Health Institute, Emory University, in collaboration with the Directorate General of Health Services, Bangladesh. CHAMPS received funding support from the Bill & Melinda Gates Foundation. The funding was provided for training workshops in systematic review in Bangladesh, in which researchers from Bangladesh, listed as co‐authors of this manuscript, undertook training in systematic review methods.

## Conflict of interest

All authors declared to have no known competing interests.

## Ethics statement

This work is a systematic review and meta‐analysis of previously published studies; therefore, no ethics approval or consent to participate was required. All authors approved the final manuscript as submitted and consented to the publication of the work.

## Trial registration

PROSPERO CRD42022321753, Date of registration: May 2, 2022.

## Supporting information


**Table S1.** Characteristics of included studies.
**Appendix S1.** PRISMA 2020 checklist.
**Appendix S2.** Search strategies.
**Appendix S3.** A detailed description of the review methods.
**Appendix S4.** Citations of shortlisted and excluded studies.
**Appendix S5.** Results: exploration of heterogeneity.
**Appendix S6.** Summary of findings table with ratings of certainty‐of‐evidence.
**Appendix S7.** Citations of published systematic reviews that evaluated interventions to reduce screen time for children.
**Figure S1.** Funnel plot screen time.
**Figure S2.** Forest plot screen time expectation.
**Figure S3.** Funnel plot physical activity.
**Figure S4.** Forest plot physical activities expectation.
**Figure S5.** (a) Forest plot BMI overall. (b) Forest plot BMI screen focused subgroup. (c) Forest plot BMI lifestyle intervention subgroup.
**Figure S6.** Funnel plot BMI.
**Figure S7.** Forest plot participants with obesity.
**Figure S8.** Forest plot severity internet gaming disorder.
**Figure S9.** Forest plot self‐efficacy well‐being.

## Data Availability

All data, including the data extraction form, and data package in RevMan 5.4 and JASP format are available on reasonable request to the corresponding author.
