# Supplementary material for: Review Article: The effectiveness of school‐based interventions for reducing screen time – a systematic review and meta‐analysis
Source: Child Adolesc Ment Health. 2025 Jul 14;30(3):223–37. doi: 10.1111/camh.70022 (PMC12351200; doi:10.1111/camh.70022)

**The Effectiveness of School-based Interventions for Reducing Screen Time: a Systematic Review and Meta-analysis**

**Running head:** The effects of school-based screen time reduction strategies: a meta-analysis

**Supporting Information**

**Table S1.** Characteristics of included studies.

**Appendix S1.** PRISMA 2020 Checklist.

**Appendix S2.** Search Strategies.

**Appendix S3.** a detailed description of the review methods.

**Appendix S4.** Citations of shortlisted and excluded studies.

**Appendix S5.** Results: exploration of heterogeneity.

**Appendix S6.** Summary of findings table with ratings of certainty-of-evidence.

**Appendix S7.** Citations of published systematic reviews that evaluated interventions to reduce screen time for children.

**Figure S1.** Funnel plot screen time.

**Figure S2.** Forest plot screen time expectation.

**Figure S3.** funnel plot physical activity.

**Figure S4.** forest plot physical activities expectation.

**Figure S5a.** Forest plot BMI overall.

**Figure S5b.** Forest plot BMI screen focused subgroup.

**Figure S5c.** Forest plot BMI lifestyle intervention subgroup.

**Figure S6.** funnel plot BMI.

**Figure S7.** forest plot participants with obesity.

**Figure S8.** forest plot severity internet gaming disorder.

**Figure S9.** forest plot self efficacy well being.

**Table S1.** Characteristics of included studies.

| Last name of first author  (Year of Publication) | Study design | Single/  multiple schools | Total number of participants recruited | Population: Inclusion criteria | Intervention | Comparison | Outcomes: Primary | Outcomes: secondary | Funding |
| --- | --- | --- | --- | --- | --- | --- | --- | --- | --- |
| Aceves  (2022) | Cluster RCT | Multiple schools | 223 | Adolescent of 13-to-16 year old from one of the four selected high schools in Spain | Peer Adolescent Challenge Creators (ACC): exercise-related activities presented as challenges ((1.30 hours/week, during 24 weeks, conducted by health promotion and communication specialists) | No specific intervention | Number of participants who met expectations on screen or sedentary time and physical activities, number of participants who met expectations on fruits and vegetable consumption* | Not stated | European Commission (European Directorate General HEALTH-2012 12 19) |
| Agbaria (2022) | RCT | Single school | 160 | 7th to 9th graders from eight schools in northern Israel. Participants were eligible if they exhibited high scores on Young’s (1998) Internet Addiction Test questionnaire, indicating elevated symptoms of Internet addiction | 12 weekly cognitive-behavioural treatment targeting internet addiction | Weekly classroom conversation | Internet addiction (Internet Addiction Questionnaire), self-control (The Adolescent self-control scale)* | Not stated | Not stated |
| Ahmed (2022) | Cluster RCT | Multiple schools | 320 | Students of grade 8 and 9 (aged 13–17 years) from public and private high schools in Dhaka city, Bangladesh | Weekly supervised circuit exercise (30 min/week), health education session (10 min/week) with health educational materials, and lunchtime sports activities (20 min/week) exercise (30 min/week) | No specific intervention | Self-reported amount of physical activities and screen time | Not stated | There was no external ﬁnancial support |
| Aittasalo (2019) | Cluster RCT | Multiple schools | 1550 | Students of 8th grades (aged 14) from all 14 public secondary schools in Tampere, Finland | Three scheduled lessons of increasing physical activities and reducing sedentary lifestyle that were integrated into the curriculum | Standard health education lessons | Number of days per week with more than 2 hours of screen time, number of days per week with at least 1 hour of brisk leisure physical activities, number of days per week walking or cycling to school* | Not stated | Ministry of Education and Culture, Finland |
| Amini (2015) | Cluster RCT | Multiple schools | 334 | Primary school students from Iran who were obese and overweight | The intervention includes three components: education of nutrition for students, life style modification for parents, increased physical activity and changing canteens content | Not stated | Body mass index (BMI) z score*, waist and hip circumference*, triceps skinfold thickness*, time spent working with computer*, time spent watching TV*, physical activities and vigorous physical activities*, energy and fat intake* | Not stated | Not stated |
| Andrade (2015) | Cluster RCT | Multiple schools | 2054 | Students of 8th and 9th grade from 20 schools in Cuenca, Ecuador | Two-stage educational programme consisting in setting individual strategies in increasing physical activity and reducing screen time, and overcoming barriers to effective implementation of individual strategies. Total duration of intervention: 28 months (around 2 academic years) | No specific intervention | Total screen-time and specific screen time components such as time on TV, video games and computer | Not stated | VLIR-UOS and Nutrition Third World and conducted within the IUC cooperation between the Cuenca University (Ecuador) and Ghent University (Belgium). Authors MV is supported by the Research Foundation Flanders (FWO) |
| Babic (2016) | Cluster RCT | Multiple schools | 322 | Students of grade 7 from secondary schools in Newcastle, Hunter and Central Coast of New South Wales, Australia, who reported ≥2 h/day of recreational screen time | The S4HM intervention: Self-Determination Theory (SDT) and includes the following components: an interactive seminar for students, eHealth messaging, behavioural contract and parental newsletters. Duration of intervention was 6 months | Not stated | Recreational screen time measured by the Adolescent Sedentary Activity Questionnaire (ASAQ) | Physical activity, BMI, mental health-related outcomes (psychological well-being, psychological distress*, psychological difficulties*, physical self-perception*) | Hunter Medical Research Institute (HMRI) grant. Authors DRL is funded by an Australian Research Council Future Fellowship. RCP and ALB are funded by Senior Research Fellowships from the National Health and Medical Research Council of Australia |
| Bagherniya (2018) | Cluster RCT | Multiple schools | 172 | Overweight and obese adolescents (between 12-16 yr of age) from schools with the same socioeconomic background in Shahin Shahr, Iran | A multi-faceted lifestyle intervention for 30 weeks covering sports workshops, physical-activity consulting private sessions, free practical and competitive sports sessions, family exercise sessions, text messages, and newsletters | Not stated | BMI, waist circumference* | Physical activity, sedentary time, physical activity self-efficacy, social support* and other psychological outcomes* (outcome expectations (i.e., perceived benefits) and outcome expectancies (i.e., values placed on benefits), intention (i.e., proximal goals) and perceived barriers) | Grant by Tehran University of Medical Sciences |
| Barbosa (2019) | Cluster RCT | Multiple schools | 1085 | Students from grades 7-9 from six schools in Brazil that joined the School Health Program (Programa Saúde na Escola, PSE) | Multi-component lifestyle intervention consisting in teacher training, health education, and environmental changes. (The Fortaleça suaSaúde program) | Standard curriculum | The proportion of adolescents who met screen time and physical activity guidelines, proportion of adolescents who met food, fruit and vegetable consumption guidelines* | Not stated | Study not funded but individual grants received from the Coordenação de Aperfeiçoamento de Pessoal de Nível Superior and Fundação de Amparo à Pesquisa e Inovação do Estado de Santa Catarina |
| Bergh (2014) | Cluster RCT | Multiple schools | 1580 | Sixth-graders from 37 schools in seven counties in the south-eastern part of Norway | Health in Adolescent (HEIA) study, a multi-component educational and behavioural intervention aimed primarily for obesity prevention. It consisted of individual-, group-, and environmental strategies to promote a healthy development in the participants’ dietary, physical activity and screen time behaviours during school hours and in leisure time | Standard curriculum | Amount of time on TV and computer game | Parental factors in mediating the participant's media use* | Funded jointly by the Norwegian Research Council (grant number 175323/V50) and the Throne Holst Nutrition Research Foundation, University of Oslo and the Norwegian School of Sport Sciences. "The paper was prepared in cooperation with the ENERGY project. The ENERGY project is funded by the Seventh Framework Programme (CORDIS FP7) of the European Commission, HEALTH (FP7-HEALTH-2007-B) grant number 223254. The content of this article reflects only the authors’ views and the European commission is not liable for any use that may be made of the information contained therein. In addition, part of the work on the paper was financially supported by the Netherlands Organization for Health Research and Development (grant number ZonMw 121.520.002) and the World Cancer Research Fund (grant number 2008/65) |
| Bickham (2018) | Cluster RCT | Multiple schools | 529 | Students of grade 6-8 from two middle schools from the same rural area in USA | Take the Challenge (Ttc) intervention: an educational programme that is integrated into students' classroom work, aiming to educate children about the health effects of excessive screen media use and to give them the experience of reducing their screen media exposure for a 10-day period | Standard curriculum | Student-reported electronic media use (television, video games, Internet) | Student health behaviours (sleep, exercise, and outdoor play)* and school behaviour* | Supported by the United States Department of Education: Office of Safe and Drug Free Schools, Carol M. White Physical Education Program (PEP) Grant [Q215F050099]; Delta-Schoolcraft Intermediate School District, and Lt. Colonel David Grossman |
| Brandstetter (2012) | Cluster RCT | Multiple schools | 1119 | First-graders from all schools within the Ulm region, Germany | The "URMEL-ICE" intervention focused on health-promoting behaviour change in three areas: drinking sugar-sweetened beverages, spending time with screen media and being physically active. The URMEL-ICE-intervention consists of material for 1 school year with 29 teaching modules | Not stated | BMI | Subscapular and triceps skinfold thickness and waist circumference* | Funded by Baden-Württemberg Stiftung (Stuttgart, Germany) |
| Centis (2012) | Cluster RCT | Multiple schools | 209 | Children of fourth grade of primary school in Bologna, Italy | Multi-component healthy lifestyle intervention focusing on nutrition and physical activity. The main activities included parental and teacher information sessions, counselling and weekly follow-up telephone monitoring. Duration of intervention was 5 months | Only information sessions without counselling and follow-up | BMI, waist and hip circumference*, triceps skinfold thickness*, waist-to-hip and waist-to-height ratios* | Physical activity (open-air games) and TV watching | Fondazione Cassa di Risparmio in Bologna, Bologna, Italy |
| Champion (2023) | Cluster RCT | Multiple schools | 9280 | Year 7-8 students (aged 11-14) from 85 schools across four sites (Sydney, rural New South Wales, Brisbane and Perth) in Australia | Health4Life, a six-module e-Health multiple health behaviour change (MHBC) programme with targeted education about the Big 6 key risk factors for health (Alcohol use, tobacco smoking, recreational screen time, physical inactivity, poor diet, and poor sleep) and developing healthy habits, using stories in cartoon format. Web-based targeted feedback about adhering to national health guidelines. Optional online and teacher-delivered activities to reinforce key messages and an accompanying smartphone app to encourage behaviour change by prompting students to track their behaviours and providing goalsetting opportunities, motivational quotes, and badges or rewards. Total duration of intervention: 24 months | Standard health education curriculum | Change in Big 6 risk factors, including moderate to vigorous physical activity (no of days per week), median daily screen hours, median daily sleep hours*, alcohol use in the past 6 months*, tobacco use in the past 6 months*, frequency of sugar-sweetened beverage consumption*, measured at baseline, 12 and 24 months (outcome data from 24 months were included in meta-analysis). | Knowledge about the Big 6 (20-item scale developed to reflect the intended content of Health4Life) | Funded by the Paul Ramsay Foundation, the Australian National Health and Medical Research Council and the Centre of Research Excellence in the Prevention and Early Intervention in Mental Illness and Substance Use, the Australian Government Department of Health and Aged Care, and the US National Institutes of Health |
| Chavarro (2005) | Cluster RCT | Multiple schools | 508 | Premenarchal girls aged 10-13 years from ten schools from four communities in Boston, USA | Planet Health: a comprehensive lifestyle intervention designed to reduce obesity in adolescents by decreasing television viewing, increasing moderate and vigorous physical activity, decreasing consumption of high-fat foods, and increasing the consumption of fruits and vegetables. Duration of intervention was 19 months (2 academic years) | Standard curriculum. Participants in the control group were offered the intervention after the study ended two school years later | Self-reported screen time, physical activity, BMI, menarchal status*, triceps skinfolds* | Not stated | Not stated |
| Chin (2008) | Cluster RCT | Multiple schools | 854 | Students of first-year (aged 12-13) from 18 secondary schools around Amsterdam, the Netherlands | DOit programme, an educational program on healthy lifestyle covering eleven lessons involving the encouragement of additional physical education classes and changes at school cafeterias | Standard curriculum | Screen-viewing behaviour (min/day), active transport to school (min/day), sugar-containing beverage (ml/day)* and high-calorie snack consumption (portion/day)* | Not stated | Funded by the Netherlands Heart Foundation (No: 2000Z002), the Dutch Ministry of Health, Welfare, and Sports, and the Royal Association of Teachers of Physical Education |
| Cong (2012) | Quasi-experimental cluster trial | Multiple schools | 416 | Children aged 5 to 9 years (from elementary school to second grade) with low-income parents recruited from Hispanic communities in Lubbock and San Elizario school districts, USA | A multicomponent lifestyle intervention program (nutrition education, exercise, gardening, and family involvement) containing 10 lessons as part of science or other classes, with monthly "Food, Fun, & Fitness" newsletters and home visits | Not stated | Sedentary behaviours reported by parents (daily screen time per week) (reported in graphical form with insufficient details to be extracted for meta-analysis)* | Not stated | Funded by the National Research Initiative of the USDA Cooperative State Research, Education and Extension Service, grant#2006-55215-16691 |
| De Coen (2012) | Cluster RCT | Multiple schools | 1102 | Participants were pre-primary and primary school students (aged 3-6) from six communities in lower socio-economic status in Belgium | A multi-component two-year intervention based on the nutrition and physical activity aiming to increase water, milk, fruits and vegetables and decrease soft drink, sweets and savoury snack, increasing physical activities and decreasing screen time | Standard curriculum | BMI, physical activities and screen time, fruits and vegetables, water, soft drink, sweets and savoury snack consumption* | Not stated | The study was commissioned, ﬁnanced and steered by the Ministry of the Flemish Community (Department of Economics, Science and Innovation; Department of Welfare, Public Health and Family) |
| dos Santos (2021) | Cluster RCT | Multiple schools | 921 | Students of 7th to 9th grades from six public municipal schools in Florianopolis, Brazil | Movimente, a multicomponent school-based intervention that consisted of teacher training, education curriculum, and environmental improvements, focusing on mainly sedentary behaviour with contents on physical activity, healthy eating and the relationship between physical activity and academic achievement, with email and personal support and follow-up. Duration of intervention: one academic year | Not stated | Self-reported screen time via questions based on the Youth Risk Behaviour Survey Questionnaire. | Not stated | Funded by the Brazil National Council for Scientific and Technological Development |
| Gortmaker (1999) | Quasi-experimental cluster trial | Multiple schools | 479 | Students in grades 4 and 5 from public schools in Baltimore, USA | Eat Well and Keep Moving Program, focusing on decreasing consumption of food with high calorie and saturated fat, increasing fruit intake and vegetable, reducing television viewing and increasing physical activity | No specific intervention during the period of the study. Intervention materials and training were provided to control schools at the end of the study | Sedentary time, physical activity, dietary intake*. | Additional food frequency and activities measures* | Grant from the Walton Family Foundation, Bentonville, Ark |
| Gortmaker (1999) | Cluster RCT | Multiple schools | 1295 | Students of grades 6 and 7 from 10 public school from 4 communities in Massachusetts, USA | Planet Health Intervention, which focused on 4 behavioural changes: decreasing television viewing, decreasing consumption of high-fat foods, increasing fruit and vegetable intake, and increasing moderate and vigorous physical activity. Duration of intervention: 2 academic years | Usual health curiculla and PE classes | Prevalence of obesity | Behavioural changes relating to food and activity by self-admitting questionnaires* | Grant from National Institutes of Child Health & Human Development, Bethesda; & Prevention Research Center Grant from Centers of Disease Control and prevention, Atlanta |
| Harrison (2006) | Cluster RCT | Multiple schools | 312 | Students of year 4 from 9 schools in the rural areas in regions identified as areas of "greatest social disadvantage" in Ireland | ‘Switch Off—Get Active’, a 16-week controlled health education intervention, in increasing physical activity and reducing screen time and BMI in primary school children | Not stated | Screen time, physical activity, screen time, physical activity self-efficacy, BMI and aerobic fitness* | Not stated | Funded by the South Eastern Health Board under the National Cardiovascular Disease Strategy and by Technological Sector Research Strand III funding to Waterford Institute of Technology |
| Ji (2023) | RCT | Single school | 77 | Students from a secondary vocational school in China who were considered at risk of internet addiction/gaming disorder by scoring higher than 68 in the Chen Internet Addiction Scale (CIAS) | Integrated Cognitive Behavioural Therapy (ICBT) in small groups of 6-7, consisting of understanding participants' gaming patterns, development of cognitive restructuring strategies, identifying strengths, interests and goals and reviewing and consolidating changes. The intervention was delivered by trained counsellors, one of whom was a social worker. Duration of intervention: eight weeks (1.5 hours per session per week) | No active intervention (wait-list control) | Gaming disorder (CIAS, score range: 26-104) at 6 months | Gaming disorder(CIAS) at 1 and 3 months*, time spent gaming (hours per week), gaming motivation (On-line Game Psychological Needs Questionnaire (OGPNQ))*, maladaptive gaming cognition (Chinese- Revised Internet Gaming Cognition Scale (C-RIGCS))*, depression symptoms (Patient Health Questionnaire-9 (PHQ-9)), anxiety (Generalized Anxiety Disorder (GAD-7) | The authors received no financial support for the research, authorship and/or publication of this article |
| Jones (2008) | Cluster RCT | Multiple schools | 718 | Girls from 6 and 7 grades from 12 middle schools in Central Texas, USA | The IMPACT Physical Activity Intervention consisted of three major components: a health curriculum which included classroom lessons and behavioural journalism, a physical education program, and a school food service component that emphasized calcium rich food choices | Standard health curriculum | Sedentary time, moderate to vigorous physical activity (MVPA), vigorous physical activity*, weight-bearing physical activity (WBPA)* | Not stated | Funded by the National Institute of Child Health and Human Development Grant R01 HD37767-04 |
| Kocken (2006) | Cluster RCT | Multiple schools | 1112 | Children aged 9 to 11 from 45 primary schools in the Netherlands | "Extra Fit" education program, which consisted of seven lessons in the ﬁrst school year and nine in the second year focusing on physical activity, computer use, nutrition, and energy balance by using experiments, assignments, videos and classroom discussions  . | Standard curriculum | Sedentary time (inactivity and screen time), physical activity, dietary intake (food diary)* and behavioural determinants of activity* | BMI (prevalence of overweightness/obesity), waist and hip circumference* | Funded by The Netherlands Organization for Health Research and Development (grant 120610007) |
| Lawlor (2016) | Cluster RCT | Multiple schools | 2221 | School children aged 8=9 from Bristol and North Somerset areas in the UK | A lifestyle intervention programme provided by the trial manager, a nutritionist and physical education specialist, with 16 lesson-plans and teaching materials, including pictures, CDs and journals, and 10 parental-child interaction homework activities. The programme focused on increasing physical activity, reducing sedentary behaviour and improving diet | Standard curriculum | Perceived parental influence on physical activity*, fruit and vegetable consumption* | Screen time, physical activity self-efficacy, consumption of snacks and of high energy drinks* | Funded by the UK National Institute for Health Research (NIHR) Public Health Research Programme (09/3005/04).This study was undertaken in collaboration with the Bristol Randomised Trials Collaboration (BRTC), a UKCRC Registered Clinical Trials Unit in receipt of National Institute for Health Research CTU support funding |
| Lindenberg (2022) | Cluster RCT | Multiple schools | 422 | Adolescents aged 12 to 18 years in 33 high schools in the Rhine-Neckar metropolitan region in Germany who were considered at risk of gaming or internet addiction disorder | PROTECT, a cognitive behavioural therapy–based indicated preventive group intervention delivered in 4 sessions by trained psychologists. It targets changes in addictive reward processing and pathological cognitive mechanisms to prevent internet addiction or gaming disorder | No specific intervention | Internet addiction symptom severity after 12 months | Comorbid psychopathology and problem behaviours* | Funded by the Dietmar Hopp Foundation |
| Lubans (2013) | Cluster RCT | Multiple schools | 357 | Adolescent girls aged 12 to 14 years from 12 secondary schools in low-income communities in New South Wales, Australia | A multicomponent school-based intervention program tailored for adolescent girls (the NEAT girls intervention), including teacher professional development, enhanced school sport sessions, interactive seminars, nutrition workshops, lunch-time physical activity sessions, handbooks and pedometers for self-monitoring, parent newsletters, and text messaging for social support | Control group was provided with equipment packs and a condensed version of the intervention following the completion of 24-month assessments | BMI, BMI z scores* and body fat percentage* | Physical activity, screen time, self-esteem and dietary intake* | Funded by grant DP1092646 from the Australian Research Council |
| Nawi (2015) | Cluster RCT | Multiple schools | 97 | Secondary school students in Malaysia aged 16 years with a BMI of > 25 kg/m2 with access to the Internet at home | Intervention group (obeseGO!) was given internet-based intervention i.e. information on healthy lifestyle and diet provided via internet | Control group were provided with printed reading materials (pamphlets) on the same information as in the obeseGO! | BMI, waist circumference*, and the body fat percentage*after 12 weeks of intervention | Not stated | Not stated |
| Peralta (2009) | RCT | Single school | 33 | Student of 7th Grade (12–13 years) from a single-sex (boys) secondary school in Sydney, Australia  Students with the lowest scores from cardiorespiratory fitness results placing them in the bottom 50th percentile among boys this age in New South Wales, Australia were invited to participate | The intervention consisted of one 60-minute curriculum session and two 20-minute lunch time physical activity sessions per week. Total study duration: 6 months. | Standard curriculum | BMI | Screen time, physical activity, waist circumference*, cardiorespiratory fitness* | Not stated |
| Pietsch (2023) | Cluster RCT | Multiple schools | 4591 | Adolescent students from 17 vocational schools in Germany (average age at baseline: 19.2 years) | My Time-off: the Challenge: an app-based intervention developed for implementation in vocational schools, which consisted of challenges in initiating and maintaining abstinence from major adverse health behaviours such as smoking, alcohol, cannabis, digital media use and gambling. Total duration of intervention: 2 weeks | No active intervention (students given access to the app after completion of follow-up survey) | Number of participants who attained expectation of positive changes in the following: alcohol use*, substance use*, cigarette smoking*, digital media use, gaming time, gambling* | Number of participants who attained expectation of positive changes in the following: physical activity (past month), positive mental health*, general self-efficacy | Funded by the German Federal Ministry of Health (ZMVL1-2519DSM216) |
| Robinson (2001) | Cluster RCT | Multiple schools | 225 | Third-and fourth-grade children (mean age, 8.9 years) in two public schools in San Jose, CA, USA | Intervention included eighteen 30- to 50-minute lessons as part of the standard curriculum in the intervention school. The sessions focussed on screen time control to 7 hr/wk, including self-monitoring of screen time followed by TV turn-off | Standard curriculum | Children's physical and verbal aggression*; parental report of children's aggressive behaviour* | | Funded by the American Heart Association, California Affiliate |
| Robinson (2006) | Cluster RCT | Multiple schools | 181 | All third- and fourth- grade students in two public schools in one school district in San Jose, CA, USA | Student media awareness to reduce television (SMART) programme, a 18-lesson programme focusing on personal ,behavioural and environmental factors relating to screen time reduction and targeted non-selective and selective reduction of television viewing, videotape viewing, and video game use, without providing specific alternative or substitute activities | Not stated | Screen time: media use by the participant, including TV, VCR, video gaming time during weekdays and weekends, media use by parents and siblings* | Parental factors in mediating the participant's media use* | Funded in part by the American Heart Association, California Affiliate and Robert Wood Johnson Foundation Generalist Physician Faculty Scholar Award |
| Salmon (2008) | Cluster RCT | Multiple schools | 295 | Students of grade 5 (10–11 years old) from three government schools in low socioeconomic areas of Melbourne, Australia | A lifestyle intervention focusing on increasing physical activities with two components: behavioural modification and fundamental skills movement. Duration of intervention: one academic year | Standard curriculum | BMI, physical activity, self-reported screen time, self-reported satisfaction of physical activity, fundamental movement skills* | | Funded by the Victorian Health Promotion Foundation |
| Schmidt (2022) | RCT | Multiple schools | 497 | Students from 17 vocational schools in Germany (average age: 20.6) who fulfilled the criteria of internet use disorder (IUD) (assessed according to DSM-5) | Telephone counselling (up to two-weekly, each lasting 30-45 minutes) and follow-up interviews by trained psychologists using motivational interviewing techniques to reduce internet use. Total duration of intervention: up to 6 weeks, follow-up interviews were made at 5 and 10 months after commencement | No active intervention (participated in the same follow-up interviews as the intervention group) | IUD criteria change (no from 2 to 9) | Readiness to change*, self-efficacy, impairment* | The iPIN-study was funded by the German Federal Ministry of Health (Grant number ZMVI1-2517DSM210) |
| Smith (2014) | Cluster RCT | Multiple schools | 361 | Adolescent boys (aged 12–14 years) considered at risk of obesity from 14 secondary schools in low-income communities in New South Wales, Australia | Intervention that focused on reducing screen time and increasing physical activities by means of teacher professional development, provision of fitness equipment to schools, face-to-face physical activity sessions, lunchtime student mentoring sessions, researcher-led seminars, a smartphone application and web site, and parental strategies for reducing screen-time. Duration of intervention: 20 weeks | Standard curriculum. Control group received an equipment pack and a condensed version of the program after the completion of 18-month assessment | BMI, waist circumference*, percent body fat*, physical activity, screen-time, sugar-sweetened beverage intake*, muscular fitness*, and resistance training skill competency* | | Funded by an Australian Research Council Discovery Project grant (DP120100611) |
| Verswijveren (2022) | Cluster RCT | Multiple schools | 267 | Children aged 8 to 9 years from primary schools within 50 km of the Melbourne Central Business District, Australia | The study consisted of three intervention groups and a control group: group one targeted increases in physical activity (PA-I); group two targeted reductions in sedentary time (SB-I); group three targeted both physical activity and sedentary time (PA +SB-I). We extracted data from group three against the control group | Standard curriculum | Low*, moderate* and vigorous physical activities, sedentary time | | This research is secondary data analysis and did not receive any specific grant. The parent study, Transform-Us! trial was funded by the National Health & Medical Research Council (NHMRC) of Australia Project Grant (ID: 533815) and Diabetes Australia Research Trust |
| Wang (2022) | Cluster RCT | Multiple schools | 58474 | Students from 92 primary and secondary schools across seven provinces in China (Hunan, Ningxia, Tianjin, Chongqing, Liaoning, Shanghai, and Guangdong) | The intervention consisted of four components, including the provision of supportive facilities for healthy lifestyle in schools, health education and mandatory physical activities, enhanced school physical education and self-monitoring of high-risk behaviours for obesity. Specific aims included increased daily fruits and vegetables intake, reduced meat intake, reduced TV and video gaming time and increased moderate or vigorous physical activity. Total duration of intervention: five months | No active intervention | Knowledge: number who attained expectations on physical activity, diet*, obesity*, belief: number who attained expectations on the relationship of weight and diet*, practice: number who attained expected consumption of fruit and sugar-sweetened beverage. Outcomes were measured at around 6 months after study commencement | | Funded by the Research Special Fund for Public Welfare Industry of Health (201202010) and the National Natural Science Foundation of China (81673192). Capital’s Funds for Health Improvement and Research (2022-1G-4251) and the Natural Science Foundation of Beijing (Grant No. 7222247) |
| Wright (2013) | Cluster RCT | Multiple schools | 251 | Children ages 8–12 from elementary schools in urban, low-income neighbourhoods in Los Angeles, USA | Kids N Fitness©, a weekly program focusing on physical activity and healthy eating that involved parents and children. Programme included health and counselling services, staff professional development in health promotion, parental education newsletters, and provision of healthy foods in the school. Duration of intervention: six weeks | Standard curriculum | Physical activity, participation in team sports*, attendance of physical education class*, TV viewing/computer gameplaying, BMI, height*, weight*, resting blood pressure*, and waist circumference* | | This work was partially supported by a grant from the NIH/NCMHD Loan Repayment Program (data analysis and interpretation of data in the writing of the report) and a grant from the Robert Wood Johnson Foundation [Grant No. 64195] (implementation of intervention, collection, analysis and interpretation of data; in  the writing of the report) |

* Outcomes not included in our meta-analysis, either because they were not part of our pre-specified review outcomes, or because we selected other outcome within the same outcome group in our meta-analysis based on consideration of importance as well as similarity with outcomes reported by other studies

**Appendix S1.** PRISMA 2020 Checklist.

| **Section and Topic** | **Item #** | **Checklist item** | **Location where item**  **is reported** |
| --- | --- | --- | --- |
| **TITLE** | | |  |
| Title | 1 | Identify the report as a systematic review. | Title |
| **ABSTRACT** | | |  |
| Abstract | 2 | See the PRISMA 2020 for Abstracts checklist. | Abstract |
| **INTRODUCTION** | | |  |
| Rationale | 3 | Describe the rationale for the review in the context of existing knowledge. | Introduction, paragraphs 1-5 (specifically addressed in paragraph 5, lines 5-11) |
| Objectives | 4 | Provide an explicit statement of the objective(s) or question(s) the review addresses. | Introduction, paragraph 5, lines 11-13. |
| **METHODS** | | |  |
| Eligibility criteria | 5 | Specify the inclusion and exclusion criteria for the review and how studies were grouped for the syntheses. | Methods, Inclusion criteria |
| Information sources | 6 | Specify all databases, registers, websites, organisations, reference lists and other sources searched or consulted to identify studies. Specify the date when each source was last searched or consulted. | Methods, Search strategies, Appendix 3: Search strategies |
| Search strategy | 7 | Present the full search strategies for all databases, registers and websites, including any filters and limits used. | Appendix 2 |
| Selection process | 8 | Specify the methods used to decide whether a study met the inclusion criteria of the review, including how many reviewers screened each record and each report retrieved, whether they worked independently, and if applicable, details of automation tools used in the process. | Methods, Screening and selection, data extraction and risk-of-bias assessment, Appendix 3: Data selection, extraction and coding |
| Data collection process | 9 | Specify the methods used to collect data from reports, including how many reviewers collected data from each report, whether they worked independently, any processes for obtaining or confirming data from study investigators, and if applicable, details of automation tools used in the process. | Methods, Screening and selection, data extraction and risk-of-bias assessment, Appendix 3: Data selection, extraction and coding |
| Data items | 10a | List and define all outcomes for which data were sought. Specify whether all results that were compatible with each outcome domain in each study were sought (e.g. for all measures, time points, analyses), and if not, the methods used to decide which results to collect. | Methods, Primary outcomes, secondary outcomes, Appendix 3: Primary outcomes, secondary outcomes. |
|  | 10b | List and define all other variables for which data were sought (e.g. participant and intervention characteristics, funding sources). Describe any assumptions made about any missing or unclear information. | Methods, Inclusion criteria, Appendix 3: Inclusion criteria- population, intervention, comparison, Dealing with missing data. |
| Study risk of bias assessment | 11 | Specify the methods used to assess risk of bias in the included studies, including details of the tool(s) used, how many reviewers assessed each study and whether they worked independently, and if applicable, details of automation tools used in the process. | Methods, Screening and selection, data extraction and risk-of-bias assessment, Appendix 3: Risk-of-bias assessment |
| Effect measures | 12 | Specify for each outcome the effect measure(s) (e.g. risk ratio, mean difference) used in the synthesis or presentation of results. | Methods, Assessment of heterogeneity, meta-analysis and certainty-of-evidence rating, Appendix 3: Strategy for data synthesis, paragraph 3. |
| Synthesis methods | 13a | Describe the processes used to decide which studies were eligible for each synthesis (e.g. tabulating the study intervention characteristics and comparing against the planned groups for each synthesis (item #5)). | Appendix 3: Strategy for data synthesis, paragraph 1. |
|  | 13b | Describe any methods required to prepare the data for presentation or synthesis, such as handling of missing summary statistics, or data conversions. | Appendix 3: Strategy for data synthesis, paragraphs 3 and 4. |
|  | 13c | Describe any methods used to tabulate or visually display results of individual studies and syntheses. | Appendix 3, Strategy for data synthesis. |
|  | 13d | Describe any methods used to synthesize results and provide a rationale for the choice(s). If meta-analysis was performed, describe the model(s), method(s) to identify the presence and extent of statistical heterogeneity, and software package(s) used. | Methods, Assessment of heterogeneity, meta-analysis and certainty-of-evidence rating, Appendix 3: Strategy for data synthesis, paragraph 2; Strategy for dealing with heterogeneity. |
|  | 13e | Describe any methods used to explore possible causes of heterogeneity among study results (e.g. subgroup analysis, meta-regression). | Methods, Assessment of heterogeneity, meta-analysis and certainty-of-evidence rating, Appendix 3: Strategy for dealing with heterogeneity. |
|  | 13f | Describe any sensitivity analyses conducted to assess robustness of the synthesized results. | Appendix 3: Sensitivity analysis. |
| Reporting bias assessment | 14 | Describe any methods used to assess risk of bias due to missing results in a synthesis (arising from reporting biases). | Appendix 3: Assessment of reporting bias (we included a statement stating that we did not specifically assess risk-of-bias of missing evidence using tools such as RoB-ME). |
| Certainty assessment | 15 | Describe any methods used to assess certainty (or confidence) in the body of evidence for an outcome. | Methods, Assessment of heterogeneity, meta-analysis and certainty-of-evidence rating, Appendix 3: Assessment of the certainty-of-evidence. |

| **Section and Topic** | **Item #** | **Checklist item** | **Location where item**  **is reported** |
| --- | --- | --- | --- |
| **RESULTS** | | |  |
| Study selection | 16a | Describe the results of the search and selection process, from the number of records identified in the search to the number of studies included in the review, ideally using a flow diagram. | Results: paragraph 1, Figure 1. |
|  | 16b | Cite studies that might appear to meet the inclusion criteria, but which were excluded, and explain why they were excluded. | Results, Excluded studies, Appendix 4. |
| Study characteristics | 17 | Cite each included study and present its characteristics. | Results, Included studies, eTable 1. |
| Risk of bias in studies | 18 | Present assessments of risk of bias for each included study. | Results, risk-of-bias assessment, Figure 2, Figure 3. |
| Results of individual studies | 19 | For all outcomes, present, for each study: (a) summary statistics for each group (where appropriate) and (b) an effect estimate and its precision (e.g. confidence/credible interval), ideally using structured tables or plots. | Results, Effect estimates. |
| Results of syntheses | 20a | For each synthesis, briefly summarise the characteristics and risk of bias among contributing studies. | Results, Effect estimates. |
|  | 20b | Present results of all statistical syntheses conducted. If meta-analysis was done, present for each the summary estimate and its precision (e.g. confidence/credible interval) and measures of statistical heterogeneity. If comparing groups, describe the direction of the effect. | Results, Effect estimates. |
|  | 20c | Present results of all investigations of possible causes of heterogeneity among study results. | Results: Effect estimates, |
|  | 20d | Present results of all sensitivity analyses conducted to assess the robustness of the synthesized results. | Methods: Sensitivity analysis (unable to be conducted), Results: NA |
| Reporting biases | 21 | Present assessments of risk of bias due to missing results (arising from reporting biases) for each synthesis assessed. | Methods: Assessment of publication or reporting bias (RoB-ME not used). |
| Certainty of evidence | 22 | Present assessments of certainty (or confidence) in the body of evidence for each outcome assessed. | Results, Effect estimates. |
| **DISCUSSION** | | |  |
| Discussion | 23a | Provide a general interpretation of the results in the context of other evidence. | Discussion, paragraph 1 |
|  | 23b | Discuss any limitations of the evidence included in the review. | Discussion, paragraph 1 |
|  | 23c | Discuss any limitations of the review processes used. | Discussion, paragraph 4 |
|  | 23d | Discuss implications of the results for practice, policy, and future research. | Conclusions |
| **OTHER INFORMATION** | | |  |
| Registration and protocol | 24a | Provide registration information for the review, including register name and registration number, or state that the review was not registered. | Methods, paragraph 1 |
|  | 24b | Indicate where the review protocol can be accessed, or state that a protocol was not prepared. | Methods, paragraph 1 |
|  | 24c | Describe and explain any amendments to information provided at registration or in the protocol. | Appendix 3 (at the end of each section where applicable, under the heading of ‘Differences between review and protocol”). |
| Support | 25 | Describe sources of financial or non-financial support for the review, and the role of the funders or sponsors in the review. | Acknowledgment |
| Competing interests | 26 | Declare any competing interests of review authors. | Declaration of competing interest |
| Availability of data, code and other materials | 27 | Report which of the following are publicly available and where they can be found: template data collection forms; data extracted from included studies; data used for all analyses; analytic code; any other materials used in the review. | Data sharing statement |

*From:* Page MJ, McKenzie JE, Bossuyt PM, Boutron I, Hoffmann TC, Mulrow CD, et al. The PRISMA 2020 statement: an updated guideline for reporting systematic reviews. BMJ 2021;372:n71. doi: 10.1136/bmj.n71

For more information, visit: <http://www.prisma-statement.org/>

**Appendix S2.** Search Strategies.

**MedLine (PubMed) search strategy (adapted for PsycInfo)**

| #1 "School children"[Title/Abstract] |
| --- |
| #2 “schools"[MeSH Terms] |
| #3 child*[Title/Abstract] |
| #4 adolescent[Title/Abstract]  #5 adolescent[MeSH Terms]  #6 teenage*[Title/Abstract]  #7 teenager[MeSH Terms]  #8 youth[Title/Abstract]  #9 school-age[Title/Abstract]  #10 #1 OR #2 OR #3 OR #4 OR #5 Or #6 OR #7 OR #8 OR #9 |
| #11 "screen time"[MeSH Terms] |
| #12 "screen time"[Title/Abstract] |
| #13 "electronic media"[Title/Abstract]  #14 television[Title/Abstract]  #15 television[MeSH Terms]  #16 TV[Title/Abstract]  #17 computer*[Title/Abstract]  #18 "computers"[MeSH Terms]  #19 internet[Title/Abstract]  #20 internet[MeSH Terms]  #21 cell phones[MeSH Terms]  #22 phone*[Title/Abstract]  #23 tablet*[Title/Abstract]  #24 laptop*[Title/Abstract]  #25 gaming[Title/Abstract]  #26 "games, recreational"[MeSH Terms]  #27 "video games"[MeSH Terms]  #28 #11 OR #12 OR #13 OR #14 OR #15 OR #16 OR #17 OR #18 OR #19 OR #20 OR #21 OR #22 OR #23 OR #24 OR #25 OR #26 OR #27  #29 #10 AND #28  *For PubMed, we used PubMed Filter of “randomised controlled trials” to limit our yields to RCTs, as follows (the use of the full Cochrane Highly Sensitive Search Strategy yield too many articles (over 39,000)):*  #30 Randomized controlled trials (publication type)  #31 #29 AND #30  *For PsycInfo, there is no filter for RCT under publication type, so we tried to limit the yields to RCTs by including the following key terms in the title and abstract field, as follows:*  #30 randomised[Title/Abstract]  #31 randomly[Title/Abstract]  #32 trial[Title/Abstract]  #33 groups[Title/Abstract]  #34 #29 AND #33 |
| **Cochrane Central Register of Controlled Trials (CENTRAL) search strategy**  #1 ("School children"):ti,ab,kw  #2 MeSH descriptor: [Schools] explode all trees  #3 (child*):ti,ab,kw  #4 (adolescent):ti,ab,kw  #5 MeSH descriptor: [Schools] explode all trees  #6 (teenage*):ti,ab,kw  #7 MeSH descriptor: [Adolescent] explode all trees  #8 (youth):ti,ab,kw  #9 (school-age):ti,ab,kw  #10 #1 OR #2 OR #3 OR #4 OR #5 OR #6 OR #7 OR #8 OR #9  #11 MeSH descriptor: [Screen Time] explode all trees  #12 ("screen time"):ti,ab,kw  #13 ("electronic media"):ti,ab,kw  #14 (television):ti,ab,kw  #15 MeSH descriptor: [Television] 4 tree(s) exploded  #16 (TV):ti,ab,kw  #17 (computer*):ti,ab,kw  #18 MeSH descriptor: [Computers] explode all trees  #19 (internet):ti,ab,kw  #20 MeSH descriptor: [Internet] explode all trees  #21 MeSH descriptor: [Cell Phone] 1 tree(s) exploded  #22 (phone*):ti,ab,kw  #23 (tablet*):ti,ab,kw  #24 (laptop*):ti,ab,kw  #25 (gaming):ti,ab,kw  #26 MeSH descriptor: [Games, Recreational] explode all trees  #27 MeSH descriptor: [Video Games] explode all trees  #28 #11 OR #12 OR #13 OR #14 OR #15 OR #16 OR #17 OR #18 OR #19 OR #20 OR #21 OR #22 OR #23 OR #24 OR #25 OR #26 OR #27  #29 (reduc*):ti,ab,kw  #30 #28 AND #29  #31 #10 AND #30 |

**Appendix S3.** a detailed description of the review methods.

**Criteria for selection of studies**

**Inclusion criteria**

**Study design**

We included randomised controlled trials (RCTs) and cluster RCTs that fulfilled the following criteria:

**Population**

Students of elementary school or primary, secondary or high schools or colleges, including special schools and home schools. We excluded studies on pre-school children of as there are several published and on-going reviews covering children of this age group ^1^.

**Intervention**

Any intervention implemented at the school level with the aim of reducing screen time, either as a stand-alone or a predominant mode of intervention or as part of a multifaceted intervention package to improve health behaviour and outcomes.

**Comparison**

No intervention, or any current standard educational programme in the school.

**Primary outcomes**

1. Screen time or frequency of screen use, variously measured and reported in terms of length of time (e.g. minutes/hours) or number of use per defined time period.

2. Academic performance, measured using any assessment method established in the school.

3. Physical activity, as variously measured by the study authors using validated self-reported measures or using objective measurement via for example, wearable digital devices.

**Secondary outcomes**

1. Level of knowledge or awareness on the adverse effects of screen time

2. Level of knowledge or awareness on healthy lifestyle

3. School absentees/attendance

4. Mental health, for example, incidence of anxiety or depression

5. Incidence of bullying

6. Body mass index (BMI); (kg/m2) or BMI z-score or incidence of overweight or obesity

7. User-satisfaction of the interventional programmes on screen time reduction

**Differences between review and protocol:**

1. We excluded non-randomised cluster trials, controlled before-and-after trials and interrupted time series because of the large number of RCTs and cluster-RCTs gathered in the initial search.
2. Population: we excluded population from pre-schools, special school and home school, because of the large number of trials retrieved that focused on primary and secondary schools, and on-going reviews on pre-schools.

**Search strategies**

Without restrictions in publication period and language, we searched MEDLINE (PubMed), Cochrane Central Register of Controlled Trials (CENTRAL) (which covered EMBASE, CINAHL and trial registers including WHO International Trial Registry Platform and ClinicalTrials.gov) and PsycInfo for published studies till August 2022 (see Appendix 2 for search strategies). We also searched the reference lists of relevant reviews for additional studies.

**Differences between review and protocol:** we did not search Web of Science, Literatura Latinoamericana y del Caribe en Ciencias de la Salud (LILACS) and trial registers separately as we found Cochrane CENTRAL database to cover MedLine, EMBASE, CINAHL and trial registers, and is now recommended by Cochrane as the main database for randomised trials. A preliminary search on LILACS returned no relevant hits. We included PsycInfo following recommendations by one of the review team members. In view of the large number of initial search hits after searching MedLine (PubMed) and CENTRAL, we decided to focus on these two databases.

**Data extraction, selection and coding**

Two pairs of authors (MSA&URS, SMHM&FUH) independently screened titles and abstracts for shortlisting using Rayyan (<https://rayyan.ai/>). Two authors (YSL, PXK) evaluated shortlisted articles in full texts to determine final eligibility. We used Robot Reviewer (https://www.robotreviewer.net/) to extract the first draft of the study characteristics including population, intervention, comparison and outcomes, with extensive editing by two authors (YSL, PXK) who entered the finalised data into a dedicated spreadsheet. We resolved disagreements by discussion leading to a consensus, with referral to the third author (NML) as required.

**Risk-of-bias assessment**

Two authors (NML, PXK) independently assessed the risk-of-bias of included studies using the Cochrane risk-of-bias 2 (rob 2) tool ^2^, using the resources provided in the riskofbias.info website (https://www.riskofbias.info/). The tool consisted of five domains (randomisation process, deviation from intended interventions, missing outcome data, measurement of the outcome, selection of the reported results) for individually randomised trials and six domains for cluster trials (randomisation process, timing of identification or recruitment of participants, deviation from intended interventions, missing outcome data, measurement of the outcome, selection of the reported results), with a series of signalling questions within each domain.

We discussed any disagreement leading to a consensus and if necessary, the involvement of the third review author (NC).

**Differences between review and protocol:** we used only the Cochrane RoB 2 tool for risk of bias assessment, and did not use other tools mentioned in the protocol (ROBINS-I tool and Joanna Briggs Institute Checklist) following our decision to include only RCTs and cluster-RCTs.

**Dealing with missing data**

We determined the dropout rates from each study and assessed the number of participants that were initially randomised against the total number analysed. We considered a dropout rate greater than 20% as significant ^3^. We determined that if we found a significant dropout rate with no reasonable explanation, or markedly different dropout rates between the assigned groups, we would judge the study at high risk in the domain of missing outcome data in our risk-of-bias assessment. If we considered the extent of missing data to be critical to the final estimates in our meta-analysis, we would have contacted the authors of the individual studies to request further information. In this review, we did not consider the proportion of missing data to be critical, and so we did not contact any study author for further information.

**Strategy for dealing with heterogeneity**

We visually inspected the forest plots for any evidence of heterogeneity. We used the I^2^ statistics to measure heterogeneity among the trials in each analysis. We identified and measured clinical, methodological and statistical heterogeneity as recommended in the Cochrane Handbook for Systematic Reviews of Interventions^2^. To evaluate clinical heterogeneity, we assessed variability in the participants’ age, sex and school setting, major category of intervention delivered (namely, screen-time-focussed or a comprehensive lifestyle intervention with screen time component) and outcome measurements. We also assessed variability in the risk-of-bias of included studies. For statistical heterogeneity, the assessment was done by looking into the observed intervention effects such as mean difference, standard mean difference, relative risk and odds ratio. We classified heterogeneity with an I^2^ ^2^ as follows:

- 0% to 40%: might not be important;
- 30% to 60%: may represent moderate heterogeneity
- 50% and above: may represent substantial heterogeneity

We derived I^2^ using JASP software version 0.17.3^4^ and checked for extreme outliers in our forest plots to determine marked differences between trials results.

If substantial heterogeneity was found, we attempted to separate the analysis of the studies into subgroups according to our exploration of possible factors that could have provided a plausible explanation for the variation in study results, as described above. In this review, we found that the strongest possible explanatory factor was the type of intervention administered, namely, screen-time-focussed or comprehensive lifestyle intervention. We therefore performed subgroup analysis for the major outcomes by separating the studies in which participants received screen-time-focussed intervention and that in which participants received lifestyle intervention, and reported the results of the test of subgroup differences, obtained using RevMan 5.4 software^5^.

**Assessment of reporting bias**

We created funnel plots to screen for publication bias for outcomes in which there were 10 or more included studies, using JASP software version 0.17.3^4^. If publication bias was suggested by significant asymmetry in the funnel plot, we would have included a statement in our results with a corresponding note of caution in our result presentation and downgraded the certainty-of-evidence for concerns on strong suspicion of publication bias ^6^.

In this review, we did not specifically assess the risk-of-bias that arose from expected but missing results in a synthesis (i.e. reporting biases), using recently-established risk-of-bias tool such as RoB-ME tool ^7^.

**Strategy for data synthesis**

We tabulated the major characteristics of the intervention evaluated in each study with its comparison in our Characteristics of included studies table (eTable 1). Although we identified two major subgroups of intervention (namely, screen-time focused intervention and comprehensive lifestyle intervention with screen time reduction components), we considered that the differentiation between these two groups as not sufficiently major and at times not clear cut to warrant separate comparisons. Consequently, we grouped all studies with relevant data for meta-analysis under one comparison in a single synthesis, and only assessed the nature of intervention (whether predominantly screen-time focused or a comprehensive lifestyle intervention) via subgrouping as part of our exploration on plausible explanatory factor for heterogeneity.

There were specific instances when we exercised our judgment in selecting outcomes for our meta-analysis, among multiple similar outcomes reported in a study: TV viewing time and video gaming time without the report of total screen time^8^, as well as separate reports of weekday or weekend media use ^8,9^, under the domain of screen or sedentary time. In the former instance, we chose TV viewing for our meta-analysis, based on the consideration that TV viewing might still be more widespread than videogaming globally. In the latter instance, we chose the data for weekends, based on consistent reports showing the propensity of children to spend more time on screen during weekends^10-14^. Among studies that reported different intensities of physical activities separately (e.g. moderate to vigorous physical activities versus any physical activities) without concurrent reporting of total physical activities ^15-23^, we chose physical activities of the highest intensity (eg. moderate-to-vigorous physical activities) as we considered them as more likely to be differentiating between groups.

We perform meta-analysis via a Bayesian random effect model with weakly-informative priors assigned to model parameters. Prior distributions for the effect sizes were determined using a Cauchy distribution with mean of 0 and a scale parameter of 0.707 (0,0.707), which corresponded to a moderately wide variance of 0.5, as recommended by Gronau et al ^24^. For the associated estimation of true between-study variation of effect sizes (tau), we chose a wide and non-informative prior using the inverse gamma distribution with the shape of 1 and a scale of 0.15, as recommended by Berkaut et al ^25^. We used Markov Chain Monte Carlo (MCMC) simulation with 3,000 iterations to allow convergence in producing the analysis output.

To obtain data for meta-analysis, we derived the effect sizes of each study with their corresponding standard errors (SE) using the generic-inverse-variance method via RevMan 5.4 ^5^. For continuous outcomes, we used mean difference (MD) or standardised mean difference (SMD) depending on whether the outcomes were measured using the same or different scales among studies. As the included studies reported their outcome data using different scales for all outcomes except for BMI, we pooled the data using standardised mean difference (SMD) in almost all analyses. We defined the magnitude of SMD according to Cohen et al, with SMD of lower than 0.2 as small, 0.2 to 0.5 as medium, 0.5 to 0.8 as medium to large, and above 0.8 as large effect size ^26^. For dichotomous outcomes, we entered the effect size data from each study in the form of logOdds Ratio (logOR) and standard error (SE), derived using RevMan 5.4 ^5^. We then exponentiated the synthesised pooled estimates (logOR) to Odds Ratio (OR). All points estimates were accompanied by their 95% credible intervals (CrI).

If the required data (e.g. SE) was missing, we derived them from the available data such as SD (by multiplying the SD with the square root of the sample size in the group) or 95% CI (by dividing the distance between the two ends of the CI by 3.92). We used adjusted effect sizes of the individual articles if these were reported.

We used Forest plots to display the meta-analysis results graphically. The Forest plots contain effect estimates from individual studies as well as pooled estimate for each outcome.

All meta-analyses were conducted using the R package metaBMA ^27^ via the JASP software application (version 0.17.3)^4^.

Where there were more than two interventions evaluated ^15^, we extracted data from the group that we considered to have had the most comprehensive or intensive intervention (in this case, group three that received interventions from both group one and two) to compare against the control group.

**Differences between review and protocol:** in our protocol, we stated that we will use random effect meta-analysis but did not specify the approach used in our meta-analysis (i.e. whether frequentist or Bayesian). In our review, we have specified that we used Bayesian random effect meta-analysis with relevant information as detailed above.

**Sensitivity analysis**

Had we sufficient data, we would have performed sensitivity analyses for the primary outcomes, and any secondary outcomes with a sufficient number of studies included, to assess the impact of excluding studies with high overall risk of bias from studies with low overall risk-of-bias.

In the review, there was only one study ^28^ that was judged to have overall low risk-of-bias. We have therefore not performed any sensitivity analysis based on risk-of-bias of the studies.

**Subgroup analysis**

If there were sufficient data that allowed for differentiation of subgroups, we would have separated into subgroups based on the following criteria:

1. Studies type: RCTs versus cluster RCT (as all except two studies were cluster-RCTs or quasi-experimental cluster studies, we could not perform subgroup analysis for the major outcomes according to study type).

2. Settings: different levels of education: primary (lower vs higher), secondary (lower vs higher), high school and college (as most studies were conducted in primary school on children of eight to 14 years, we could not perform subgroup analysis for the major outcomes based on levels of education).

3. Intervention: studies that evaluated screen time reduction as a primary component versus studies that evaluated a comprehensive multifaced lifestyle intervention that included screen time reduction as a component or an outcome of interest (we performed subgroup analysis for the major outcomes based on the nature of intervention, as detailed in the exploration of heterogeneity in Appendix 5.

**Assessing the certainty-of-evidence**

We used the GRADE approach, as outlined in the GRADE Handbook, to assess the certainty of evidence ^29^ for three major outcomes of screen time, physical activities and BMI.

Two review authors (NML, PXK) independently assessed the certainty of the evidence for each of the above outcomes. We considered evidence from RCTs as high certainty to begin with, downgrading by one level for serious (or two levels for very serious) limitations based upon five considerations: design (risk of bias), inconsistency across studies, indirectness of the evidence, imprecision of estimates, and suspicion of publication bias. We used GRADEpro GDT to create summary of findings tables for the three outcomes (see Appendix 6).

We interpreted the GRADE ratings as follows.

- High certainty: we are very confident that the true effect lies close to that of the estimate of the effect.
- Moderate certainty: we are moderately confident in the effect estimate; the true effect is likely to be close to the estimate of the effect, but there is a possibility that it is substantially different.
- Low certainty: our confidence in the effect estimate is limited; the true effect may be substantially different from the estimate of the effect.
- Very low certainty: we have very little confidence in the effect estimate; the true effect is likely to be substantially different from the estimate of effect.

We justified all decisions to downgrade the certainty of the evidence using footnotes and made comments to aid the reader's understanding of the review where necessary.

**References**

1. Raj D, Mohd Zulkefli NA, Minhat HS, N. A. Parental Intervention Strategies to Reduce Screen Time Among Preschool-aged Children: A Systematic Review *Malaysian Journal of Medicine and Health Sciences.* 2022;18(6):295-304.

2. Higgins JPT, Thomas J, Chandler J, et al. Cochrane Handbook for Systematic Reviews of Interventions version 6.3 (updated February 2022). Cochrane, 2022. In. *Available from* [*www.training.cochrane.org/handbook*](file:///C:\Users\finley.overland.ACAMH\Downloads\www.training.cochrane.org\handbook)*.*: The Cochrane Collaboration; 2022.

3. Guyatt GH, Sackett DL, Cook DJ. Users' guides to the medical literature. II. How to use an article about therapy or prevention. A. Are the results of the study valid? Evidence-Based Medicine Working Group. *Jama.* 1993;270(21):2598-2601.

4. *JASP (version 0.17.3)* [computer program]. 2023.

5. *Review Manager 5 (RevMan 5)* [computer program]. Copenhagen: The Cochrane Collaboration; 2020.

6. Sterne JA, Egger M, Moher D, Boutron I. Chapter 10: Addressing reporting biases. . In: Higgins JP CR, Chandler J, Cumpston MS, ed. *Cochrane Handbook for Systematic Reviews of Interventions Version 5.2.0.* The Cochrane Collaboration 2017.

7. Page MJ, Sterne JAC, Boutron I, et al. ROB-ME: a tool for assessing risk of bias due to missing evidence in systematic reviews with meta-analysis. *BMJ (Clinical research ed).* 2023;383:e076754.

8. Robinson TN, Borzekowski DLG. Effects of the SMART Classroom Curriculum to Reduce Child and Family Screen Time. *Journal of Communication.* 2006;56(1):1-26.

9. Andrade S, Verloigne M, Cardon G, et al. School-based intervention on healthy behaviour among Ecuadorian adolescents: effect of a cluster-randomized controlled trial on screen-time. *BMC public health.* 2015;15:942.

10. Esposito F, Sanmarchi F, Marini S, et al. Weekday and Weekend Differences in Eating Habits, Physical Activity and Screen Time Behavior among a Sample of Primary School Children: The "Seven Days for My Health" Project. *International journal of environmental research and public health.* 2022;19(7).

11. Liangruenrom N, Dumuid D, Pedisic Z. Physical activity, sedentary behaviour, and sleep in the Thai population: A compositional data analysis including 135,824 participants from two national time-use surveys. *PloS one.* 2023;18(1):e0280957.

12. Sigmundová D, Badura P, Sigmund E, Bucksch J. Weekday-weekend variations in mother-/father-child physical activity and screen time relationship: A cross-sectional study in a random sample of Czech families with 5- to 12-year-old children. *European journal of sport science.* 2018;18(8):1158-1167.

13. Sigmundová D, Sigmund E. Weekday-Weekend Sedentary Behavior and Recreational Screen Time Patterns in Families with Preschoolers, Schoolchildren, and Adolescents: Cross-Sectional Three Cohort Study. *International journal of environmental research and public health.* 2021;18(9).

14. Sigmundová D, Sigmund E, Badura P, Vokáčová J, Trhlíková L, Bucksch J. Weekday-weekend patterns of physical activity and screen time in parents and their pre-schoolers. *BMC Public Health.* 2016;16(1):898.

15. Verswijveren SJJM, Ridgers ND, Martín-Fernández JA, et al. Intervention effects on children’s movement behaviour accumulation as a result of the Transform-Us! school- and home-based cluster randomised controlled trial. *International Journal of Behavioral Nutrition and Physical Activity.* 2022;19(1):76.

16. Gortmaker SL, Cheung LW, Peterson KE, et al. Impact of a school-based interdisciplinary intervention on diet and physical activity among urban primary school children: eat well and keep moving. *Archives of pediatrics & adolescent medicine.* 1999;153(9):975-983.

17. Gortmaker SL, Peterson K, Wiecha J, et al. Reducing obesity via a school-based interdisciplinary intervention among youth: Planet Health. *Archives of pediatrics & adolescent medicine.* 1999;153(4):409-418.

18. Harrison M, Burns CF, McGuinness M, Heslin J, Murphy NM. Influence of a health education intervention on physical activity and screen time in primary school children: 'Switch Off--Get Active'. *J Sci Med Sport.* 2006;9(5):388-394.

19. Jones D, Hoelscher DM, Kelder SH, Hergenroeder A, Sharma SV. Increasing physical activity and decreasing sedentary activity in adolescent girls--the Incorporating More Physical Activity and Calcium in Teens (IMPACT) study. *The international journal of behavioral nutrition and physical activity.* 2008;5:42.

20. Lubans D, Dewar D, Morgan P, et al. Two-year outcomes from the NEAT Girls obesity prevention cluster randomized controlled trial. *Journal of Science and Medicine in Sport.* 2013;16:e34.

21. Peralta LR, Jones RA, Okely AD. Promoting healthy lifestyles among adolescent boys: the Fitness Improvement and Lifestyle Awareness Program RCT. *Preventive medicine.* 2009;48(6):537-542.

22. Salmon J, Ball K, Hume C, Booth M, Crawford D. Outcomes of a group-randomized trial to prevent excess weight gain, reduce screen behaviours and promote physical activity in 10-year-old children: switch-play. *International journal of obesity (2005).* 2008;32(4):601-612.

23. Smith JJ, Morgan PJ, Plotnikoff RC, et al. Smart-phone obesity prevention trial for adolescent boys in low-income communities: the ATLAS RCT. *Pediatrics.* 2014;134(3):e723-731.

24. Gronau QF, Heck DW, Berkhout SW, Haaf JM, Wagenmakers E-J. A Primer on Bayesian Model-Averaged Meta-Analysis. *Advances in Methods and Practices in Psychological Science.* 2021;4(3):25152459211031256.

25. Berkhout SW HJ, Gronau QF, Heck AW, Wagenmakers EJ. A Tutorial on Bayesian Model-Averaged Meta-Analysis in JASP. 2022 <https://osf.io/ne8dw/download>. Accessed 6 November 2023

26. Cohen J. *Statistical Power Analysis for the Behavioral Sciences (2nd ed.).* Routledge; 1988.

27. Heck DW GQ, Wagenmakers EJ. metaBMA: Bayesian model averaging for random and fixed effects meta-analysis. 2019. <https://CRAN.R-project.org/package=metaBMA>. Accessed 6 November 2023

28. Lindenberg K, Kindt S, Szász-Janocha C. Effectiveness of Cognitive Behavioral Therapy-Based Intervention in Preventing Gaming Disorder and Unspecified Internet Use Disorder in Adolescents: A Cluster Randomized Clinical Trial. *JAMA network open.* 2022;5(2):e2148995.

29. Schünemann H, Brożek J, Guyatt G, Oxman A, editor. *Handbook for grading the quality of evidence and the strength of recommendations using the GRADE approach (updated October 2013). .* 2013.

**Appendix S4.** Citations of shortlisted and excluded studies.

**Citations of shortlisted and excluded studies**

1. Abbott MW. Television violence: A proactive prevention campaign. Abbott, Max W.: Mental Health Foundation, Director, New Zealand Thousand Oaks, CA: Mental Health Foundation of New Zealand; 1990.

2. Abbott R, Smith A, Howie E, Pollock C, Straker L. Effects of home access to active videogames on child self-esteem, enjoyment of physical activity, and anxiety related to electronic games: Results from a randomized controlled trial. Games Health J. 2014;3(4):260-266.

3. Abraham AA, Chow W, So H, et al. Lifestyle intervention using an internet-based curriculum with cell phone reminders for obese Chinese teens: a randomized controlled study. 2015;10(5):e0125673.

4. Adams E, Marini M, Stokes J, Birch L, Paul I, Savage J. INSIGHT responsive parenting intervention reduces infant's screen time and television exposure. Int J Behav Nutr Phys Act. 2018;15(24):1-9.

5. Ahmad N, Shariff ZM, Mukhtar F, Lye MS. Family-based intervention using face-to-face sessions and social media to improve Malay primary school children's adiposity: a randomized controlled field trial of the Malaysian REDUCE programme. Nutrition Journal. 2018;17(74):1-13.

6. Ahmad N, Shariff ZM, Mukhtar F, Lye MS. Effect of Family-Based REDUCE Intervention Program on Children Eating Behavior and Dietary Intake: randomized Controlled Field Trial. Nutrients. 2020;12(3065):1-13.

7. Al-Hazzaa HM, Abahussain NA, Al-Sobayel HI, Qahwaji DM, Musaiger AO. Physical activity, sedentary behaviors and dietary habits among Saudi adolescents relative to age, gender and region. Int J Behav Nutr Phys Act. 2011;8(140):1-14.

8. Al-Hazzaa HM, Albawardi NM. Activity energy expenditure, screen time and dietary habits relative to gender among Saudi youth: interactions of gender with obesity status and selected lifestyle behaviours. Asia Pac J Clin Nutr. 2019;28(2):389‐400.

9. Alt D, Boniel-Nissim M. Parent-adolescent communication and problematic Internet use: The mediating role of Fear of Missing Out (FoMO). J Fam Issues. 2018;39(13):3391-3409.

10. Altenburg TM, Kist-van Holthe J, Chinapaw MJ. Effectiveness of intervention strategies exclusively targeting reductions in children's sedentary time: A systematic review of the literature. Int J Behav Nutr Phys Act. 2016;13(65):1-18.

11. Arnaud N, Weymann J, Lochbuhler K, et al. Effectiveness of an app-based intervention to reduce substance use, gambling, and digital media use in vocational school students: study protocol for a randomized controlled trial. Trials. 2022;23(277):1-10.

12. Arora T, Hosseini-Araghi M, Bishop J, Yao G, Thomas G, Taheri S. The complexity of obesity in UK adolescents: Relationships with quantity and type of technology, sleep duration and quality, academic performance and aspiration. Pediatr Obes. 2013;8(5):358-366.

13. Arora T, Hussain S, Hubert Lam K, Yao G, Thomas G, Taheri S. Exploring the complex pathways among specific types of technology, self-reported sleep duration and body mass index in UK adolescents. Int J Obes (Lond). 2013;37(9):1254-1260.

14. Askie LM, Espinoza D, Martin A, et al. Interventions commenced by early infancy to prevent childhood obesity-The EPOCH Collaboration: An individual participant data prospective meta-analysis of four randomized controlled trials. Pediatr Obes. 2020;15(6):e12618.

15. Asplund KM, Kair LR, Arain YH, Cervantes M, Oreskovic NM, Zuckerman KE. Early childhood screen time and parental attitudes toward child television viewing in a low-income Latino population attending the Special Supplemental Nutrition Program for women, infants, and children. Child Obes. 2015;11(5):590-599.

16. Axford C, Joosten AV, Harris C. iPad applications that required a range of motor skills promoted motor coordination in children commencing primary school. Aust Occup Ther J. 2018;65(2):146‐155.

17. Azevedo KJ, Mendoza S, Fernández M, et al. Turn off the TV and dance! Participation in culturally tailored health interventions: implications for obesity prevention among Mexican American girls. Ethn Dis. 2013;23(4):452‐461.

18. Babayigit S. Does a truly symmetrically transparent orthography exist? Spelling is more difficult than reading even in an orthography considered highly transparent for both reading and spelling. Read Writ 2022;35:2453–2472.

19. Backlund C, Sundelin G, Larsson C. Effects of a 2-year lifestyle intervention on physical activity in overweight and obese children. Advances in Physiotherapy. 2011;13(3):97‐109.

20. Backlund C, Sundelin G, Larsson C. Evaluation of a 2-year family-based lifestyle intervention regarding physical activity among children with overweight and obesity. World Physical Therapy. 2011;97(1):eS94‐eS95.

21. Baer SA. Strategies of children's attention to and comprehension of television. 1997;57(11):7243.

22. Baiden P, Tadeo SK, Peters KE. The association between excessive screen-time behaviors and insufficient sleep among adolescents: Findings from the 2017 youth risk behavior surveillance system. Psychiatry Res. 2019;281:112586.

23. Bar-Or O, Foreyt J, Bouchard C, et al. Physical activity, genetic, and nutritional considerations in childhood weight management. Med Sci Sports Exerc. 1998;30(1):2-10.

24. Barbee SS. Integrative technology-enhanced physical education: An exploratory study with elementary school students. 2017:1-78.

25. Bauer KW, Neumark-Sztainer D, Fulkerson JA, Hannan PJ, Story M. Familial correlates of adolescent girls' physical activity, television use, dietary intake, weight, and body composition. Int J Behav Nutr Phys Act. 2011;8(25):1-10.

26. Bayer O, von Kries R, Strauss A, et al. Short- and mid-term effects of a setting based prevention program to reduce obesity risk factors in children: a cluster-randomized trial. Clin Nutr. 2009;28(2):122-128.

27. Baylis AL, Shriberg LD. Estimates of the prevalence of speech and motor speech disorders in youth with 22q11.2 deletion syndrome. Am J Speech Lang Pathol. 2019;28(1):53-82.

28. Bazzaz MM, Fadardi JS, Parkinson J. Efficacy of the attention control training program on reducing attentional bias in obese and overweight dieters. Appetite. 2017;108:1‐11.

29. Beales J, Kulick R. Does advertising on television cause childhood obesity? A longitudinal analysis. J Public Policy Mark. 2013;32(2):185-194.

30. Beaulac J, Kristjansson E, Calhoun M. 'Bigger than hip-hop?' impact of a community-based physical activity program on youth living in a disadvantaged neighborhood in Canada. J Youth Stud. 2011;14(8):961-974.

31. Beleigoli A, Andrade AQ, Diniz MF, Ribeiro AL. Personalized Web-Based Weight Loss Behavior Change Program With and Without Dietitian Online Coaching for Adults With Overweight and Obesity: randomized Controlled Trial. J Med Internet Res. 2020;22(11):e17494.

32. Bell DL, Garbers S, Catallozzi M, et al. Computer-Assisted Motivational Interviewing Intervention to Facilitate Teen Pregnancy Prevention and Fitness Behavior Changes: a Randomized Trial for Young Men. J Adolesc Health. 2018;62(3):S72‐S80.

33. Bennion KA, Tate D, Muñoz-Christian K, Phelan S. Impact of an Internet-Based Lifestyle Intervention on Behavioral and Psychosocial Factors During Postpartum Weight Loss. Obesity (Silver Spring). 2020;28(10):1860‐1867.

34. Berentzen N, Smit H, van Rossem L, et al. Screen time, adiposity and cardiometabolic markers: Mediation by physical activity, not snacking, among 11-year-old children. Int J Obes (Lond). 2014;38(10):1317-1323.

35. Blomkvist EAM, Helland SH, Hillesund ER, Øverby NC. A cluster randomized web-based intervention trial to reduce food neophobia and promote healthy diets among one-year-old children in kindergarten: study protocol. BMC Pediatrics. 2018;18(18):232.

36. Bonney E, Ferguson G, Smits-Engelsman B. The efficacy of two activity-based interventions in adolescents with Developmental Coordination Disorder. Res Dev Disabil. 2017;71:223‐236.

37. Bonte ML, Blomert L. Developmental dyslexia: ERP correlates of anomalous phonological processing during spoken word recognition. Brain Res Cogn Brain Res. 2004;21(3):360-376.

38. Boone JE, Gordon-Larsen P, Adair LS, Popkin BM. Screen time and physical activity during adolescence: Longitudinal effects on obesity in young adulthood. Int J Behav Nutr Phys Act. 2007;4(26):1-10.

39. Brambilla P, Bedogni G, Buongiovanni C, et al. "Mi voglio bene": a pediatrician-based randomized controlled trial for the prevention of obesity in Italian preschool children. Ital J Pediatr 2010;36(55):1-5.

40. Brown B, Harris KJ, Heil D, et al. Feasibility and outcomes of an out-ofschool and home-based obesity prevention pilot study for rural children on an American Indian reservation. Pilot Feasibility Stud. 2018;4(129):1-12.

41. Bryant JA, Bryant J. Effects of entertainment televisual media on children. Bryant, J. Alison: University of Southern California, Los Angeles, CA, US Bryant, Jennings: University of Alabama, AL, US Mahwah, NJ: Lawrence Erlbaum Associates Publishers; US; 2003.

42. Buchanan LR, Rooks-Peck CR, Finnie RKC, et al. Reducing children's recreational sedentary screen time: Recommendation of the community preventive services task force. American journal of preventive medicine. 2016;50(3):402-415.

43. Bucksch J, Sigmundova D, Hamrik Z, et al. International trends in adolescent screen-time behaviors from 2002 to 2010. J Adolesc Health. 2016;58(4):417-425.

44. Buscemi J, Yurasek AM, Dennhardt AA, Martens MP, Murphy JG. A randomized trial of a brief intervention for obesity in college students. Clin Obes. 2011;1(4-6):131‐140.

45. Byrd-Bredbenner C, Santiago E, Eck KM, et al. HomeStyles-2: randomized controlled trial protocol for a web-based obesity prevention program for families with children in middle childhood. Contemp Clin Trials. 2022;112(106644).

46. Calear AL, Christensen H, Mackinnon A, Griffiths KM, O'Kearney R. The YouthMood Project: a cluster randomized controlled trial of an online cognitive behavioral program with adolescents. J Consult Clin Psychol. 2009;77(6):1021‐1032.

47. Calvert SL, Valkenburg PM. The influence of television, video games, and the internet on children's creativity. Calvert, Sandra L.: Children's Digital Media Center, Department of Psychology, Georgetown University, Washington, DC, US Valkenburg, Patti M.: Amsterdam School of Communications Research, Center for Children, Adolescents, and the Media, University of Amsterdam, Amsterdam, Netherlands New York, NY: Oxford University Press; US; 2013.

48. Cameron JD, Maras D, Sigal RJ, et al. The mediating role of energy intake on the relationship between screen time behaviour and body mass index in adolescents with obesity: The HEARTY study. Appetite. 2016;107:437-444.

49. Campbell KJ, Lioret S, McNaughton SA, et al. A parent-focused intervention to reduce infant obesity risk behaviors: a randomized trial. Pediatrics. 2013;131(4):652-660.

50. Carlson SA, Fulton JE, Lee SM, Foley JT, Heitzler C, Huhman M. Influence of limit-setting and participation in physical activity on youth screen time. Pediatrics. 2010;126(1):e89-e96.

51. Carson V, Janssen I. Neighborhood disorder and screen time among 10-16 year old Canadian youth: A cross-sectional study. Int J Behav Nutr Phys Act. 2012;9(66):1-11.

52. Cascarelli NV, Jr. The impact of physical education on childhood obesity in Ohio school children: A hierarchical analysis. Int J Public Health. 2022;836(1):63-71.

53. Cespedes EM, Horan CM, Gillman MW, et al. Participant characteristics and intervention processes associated with reductions in television viewing in the High Five for Kids study. 2014;62:64‐70.

54. Charlton T, Coles D, Lovemore T. Teachers' ratings of nursery class children's behaviour before and after availability of television by satellite. Psychol Rep. 1997;81(1):96-98.

55. Chortatos A, Henjum S, Torheim LE, Terragni L, Gebremariam MK. Comparing three screen-based sedentary behaviours' effect upon adolescents' participation in physical activity: The ESSENS study. PLoS One. 2020;15(11):e0241887.

56. Corkum P, Elik N, Blotnicky-Gallant PAC, McGonnell M, McGrath P. Web-based intervention for teachers of elementary students with ADHD: randomized controlled trial. J Atten Disord. 2019;23(3):257‐269.

57. de Visser R, Sylvester R, Rogers R, et al. Changes in school health program improve middle school students' behaviors. Am J Health Behav. 2016;40(5):568-577.

58. Delmas C, Platat C, Schweitzer B, Wagner A, Oujaa M, Simon C. Association between television in bedroom and adiposity throughout adolescence. Obesity (Silver Spring). 2007;15(10):2495-2503.

59. Dennison BA, Russo TJ, Burdick PA, Jenkins PL. An intervention to reduce television viewing by preschool children. Arch Pediatr Adolesc Med. 2004;158(2):170‐176.

60. dos Santos PC, Barbosa Filho VC, da Silva JA, Bandeira AdS, Minatto G, da Silva KS. What works in sedentary behavior interventions for youth: A review of reviews. Adolescent Res Rev. 2019;4:267-292.

61. Downing KL, Salmon J, Hinkley T, Hnatiuk JA, Hesketh KD. A mobile technology intervention to reduce sedentary behaviour in 2- to 4-year-old children (Mini Movers): study protocol for a randomised controlled trial. Trials. 2017;18(1):97.

62. Kaess M, Fischer G. Short term therapy in adolescents with self-destructive and risk-taking behaviours. 2012.

63. Briegel I, Kobel S. "Healthy Eating, Active Play - Promoting a healthy lifestyle in Young CHILDren (HEALTH SURVEY)" – Study for the investigation of efficacy and cost effectiveness of the prevention program "Join the healthy boat - kindergarten". 2016.

64. Dumuid D, Olds T, Martin-Fernandez J-A, Lewis LK, Cassidy L, Maher C. Academic performance and lifestyle behaviors in Australian school children: A cluster analysis. Health Educ Behav. 2017;44(6):918-927.

65. Emerson JF, Welch M, Rossman WE, et al. A multidisciplinary intervention utilizing virtual communication tools to reduce health disparities: a pilot randomized controlled trial. Int J Environ Res Public Health. 2015;13(1):1-14.

66. Fernández-Ruiz VE, Solé-Agustí M, Armero-Barranco D, Cauli O. Weight loss and improvement of metabolic alterations in overweight and obese children through the I(2)AO(2) family program: A randomized controlled clinical Ttial. Biol Res Nurs. 2021;23(3):488-503.

67. Fitzpatrick C, Pagani LS, Barnett TA. Early childhood television viewing predicts explosive leg strength and waist circumference by middle childhood. Nutr Phys Act. 2012;9(87):1-6.

68. Foley L, Ni Mhurchu C, Marsh S, et al. Screen time weight-loss intervention targeting children at home (SWITCH): process evaluation of a randomised controlled trial intervention. BMC public health. 2016;16(439):2-9.

69. Ge Y, Xin S, Luan D, et al. Independent and combined associations between screen time and physical activity and perceived stress among college students. Addict Behav. 2020;103(106224):1-7.

70. Goran MI, Reynolds K. Interactive multimedia for promoting physical activity (IMPACT) in children. Obes Res. 2005;13(4):762-771.

71. Granich J, Rosenberg M, Knuiman M, Timperio A. Understanding children's sedentary behaviour: A qualitative study of the family home environment. Health Educ Res. 2010;25(2):199-210.

72. Grieco LA, Jowers EM, Errisuriz VL, Bartholomew JB. Physically active vs. sedentary academic lessons: a dose response study for elementary student time on task. Prev Med 2016;89:98‐103.

73. Haley SM, Fragala-Pinkham M, Ni P. Sensitivity of a computer adaptive assessment for measuring functional mobility changes in children enrolled in a community fitness programme. Clin Rehabil. 2006;20(7):616-622.

74. Halken S, Winnergard I, Laursen MK, Riis B, De Blic J. Long-term sustained reduction of allergic rhinoconjunctivitis in children with grass pollen allergy-results from an asthma prevention (GAP) trial. 2017;72:65.

75. Halkjelsvik T, Scheffels J. Standardised snus packaging reduces brand differentiation: a web-based between-subject experiment. BMC public health. 2019;19(1414):1-7.

76. Hall AB, Ho C, Albanese B, et al. User-driven design of child restraint information to reduce errors in use: a pilot randomised controlled trial. Inj Prev. 2020;26(5):432-438.

77. Hall CL, Davies EB, Andren P, et al. Investigating a therapist-guided, parent-assisted remote digital behavioural intervention for tics in children and adolescents - 'Online Remote Behavioural Intervention for Tics' (ORBIT) trial: protocol of an internal pilot study and single-blind randomised controlled trial. BMJ Open. 2019;9(1):e027583.

78. Hall CL, Valentine AZ, Walker GM, et al. Study of user experience of an objective test (QbTest) to aid ADHD assessment and medication management: a multi-methods approach. BMC Psychiatry. 2017;17(66):1-12.

79. Hall CL, Walker GM, Valentine AZ, et al. Protocol investigating the clinical utility of an objective measure of activity and attention (QbTest) on diagnostic and treatment decision-making in children and young people with ADHD-'Assessing QbTest Utility in ADHD' (AQUA): a randomised controlled trial. BMj Open. 2014;4(12):e006838.

80. Hall CW. Computer-assisted instruction in reducing errors in scoring of the WISC-III. Psychological Reports. 1999;85(3):825-833.

81. Hall JA, McKee MD, Vicente MR, et al. Prospective randomized clinical trial investigating the effect of the reamer-irrigator-aspirator on the volume of embolic load and respiratory function during intramedullary nailing of femoral shaft fractures. J Orthop Trauma. 2017;31(4):200‐204.

82. Hall JR, Cuthill IC, Baddeley R, Attwood AS, Munafo MR, Scott-Samuel NE. Dynamic dazzle distorts speed perception. PLoS One. 2016;11(5):1-19.

83. Hall SM, Tsoh JY, Prochaska JJ, et al. Treatment for cigarette smoking among depressed mental health outpatients: a randomized clinical trial. Am J Public Health. 2006;96(10):1808‐1814.

84. Hall WL, Iqbal S, Li H, Gray R, Berry SEE. Modulation of postprandial lipaemia by a single meal containing a commonly consumed interesterified palmitic acid-rich fat blend compared to a non-interesterified equivalent. Eur J Nutr. 2017;56(8):2487-2495.

85. Hallab M, Gallois Y, Chatellier G, Rohmer V, Fressinaud P, Marre M. Comparison of reduction in microalbuminuria by enalapril and hydrochlorothiazide in normotensive patients with insulin dependent diabetes. BMJ Open. 1993;306(6871):175-182.

86. Hallander HO, Ljungman M, Jahnmatz M, Storsaeter J, Nilsson L, Gustafsson L. Should fimbriae be included in pertussis vaccines? Studies on ELISA IgG anti-Fim2/3 antibodies after vaccination and infection. APMIS. 2009;117(9):660-671.

87. Haller G, Haller DM, Courvoisier DS, Lovis C. Handheld vs. laptop computers for electronic data collection in clinical research: a crossover randomized trial. J Am Med Inform Assoc. 2009;16(5):651‐659.

88. Hallett J, Maycock B, Kypri K, Howat P, McManus A. Development of a web-based alcohol intervention for university students: processes and challenges. Drug Alcohol Rev. 2009;28(1):31‐39.

89. Hankonen N, Heino MT, Hynynen ST, et al. Randomised controlled feasibility study of a school-based multi-level intervention to increase physical activity and decrease sedentary behaviour among vocational school students. Int J Behav Nutr Phys Act. 2017;14(37):1-14.

90. Hayes DS, Casey DM. Young children and television: The retention of emotional reactions. Child Dev. 1992;63(6):1423-1436.

91. Hernandez RG, Garcia JT, Amankwah EK. A look back at healthy lifestyle behavioral patterns among school-age children: Are we neglecting healthy weight for overweight kids? Child Obes. 2019;15(4):271-279.

92. Hodder RK, Freund M, Bowman J, et al. Differential intervention effectiveness of a universal school-based resilience intervention in reducing adolescent substance use within student subgroups: exploratory assessment within a cluster-randomised controlled trial. BMJ Open. 2018;8(8):e021047.

93. Högström J, Enebrink P, Melin B, Ghaderi A. Eighteen-Month Follow-Up of Internet-Based Parent Management Training for Children with Conduct Problems and the Relation of Homework Compliance to Outcome. Child Psychiatry Hum Dev. 2015;46(4):577-588.

94. Hong W, Liu R-D, Ding Y, Jiang S, Yang X, Sheng X. Academic procrastination precedes problematic mobile phone use in Chinese adolescents: A longitudinal mediation model of distraction cognitions. Addict Behav. 2021;121(106993):1-7.

95. Howie EK, Campbell AC, Straker LM. An active video game intervention does not improve physical activity and sedentary time of children at-risk for developmental coordination disorder: a crossover randomized trial. Child Care Health Dev. 2016;42(2):253‐260.

96. Huang WY, Wong SH, Salmon J. Correlates of physical activity and screen-based behaviors in Chinese children. J Sci Med Sport. 2013;16(6):509-514.

97. Iaia M, Pasini M, Burnazzi A, Vitali P, Allara E, Farneti M. An educational intervention to promote healthy lifestyles in preschool aged children. Int J Obes (Lond). 2014;41(4):582-590.

98. Isarabhakdi P, Pewnil T. Engagement with family, peers, and Internet use and its effect on mental well-being among high school students in Kanchanaburi Province, Thailand. Int J Adolesc Youth. 2016;21(1):15-26.

99. Surridge G, Brady E. A randomised controlled trial of an interactive family-based lifestyle programme. 2019(3).

100. Brown B. Generations Health Project: Promoting Child Health in Out-of-School and Home Settings. 2019(3).

101. Canário C, Cruz O. Childhood overweight and obesity intervention: effectiveness of a program based on parents as agents of change. 2020(11).

102. Carraro A. Physical education teachers' education to promote students' physical activity and to cope with sedentary behaviour. 2022(3).

103. Jackson LA, Samona R, Moomaw J, et al. What do children do on the Internet: Domains visited and their relationship to socio-demographic characteristics and academic performance. Cyberpsychol Behav. 2007;10(2):182-190.

104. James KS. A school based intervention to reduce television use decreased adiposity in children in grades 3 and 4...commentary on Robinson TN. Reducing children's television viewing to prevent obesity: a randomized controlled trial. JAMA 1999 Oct;282(16): 1561-7. 2000(5):43‐43.

105. Janz KF, Burns TL, Levy SM. Tracking of Activity and Sedentary Behaviors in Childhood: The Iowa Bone Development Study. American journal of preventive medicine. 2005;29(3):171-178.

106. Jason LA. Self-monitoring in reducing children's excessive television monitoring. Psychol Rep. 1983;53(3):1280.

107. Jerome GJ, Fink T, Brady T, et al. Physical activity levels and screen time among youth with overweight/obesity using mental health services. Int J Environ Res Public Health. 2022;19(4):1-9.

108. Jones A, Armstrong B, Weaver R, Parker H, von Klinggraeff L, Beets M. Identifying effective intervention strategies to reduce children's screen time: A systematic review and meta-analysis. Int J Behav Nutr Phys Act. 2021;18(126):1-20.

109. Kaaresen PI, Rønning JA, Tunby J, Nordhov SM, Ulvund SE, Dahl LB. A randomized controlled trial of an early intervention program in low birth weight children: outcome at 2 years. Early Hum Dev. 2008;84(3):201-209.

110. Kabir I, Rahman MM, Haider R, Mazumder RN, Khaled MA, Mahalanabis D. Increased height gain of children fed a high-protein diet during convalescence from shigellosis: a six-month follow-Up study. J Nutr. 1998;128(10):1688-1691.

111. Kable JA, Coles CD, Taddeo E, Strickland D. The GOFAR progam for children with FASD: neurocognitive and behavioral outcomes. Alcohol Clin Exp Res 2015;39(S1):167A.

112. Kabon B, Kugener A, Gruenberger T, et al. Effects of continuous remifentanil administration on intra-operative subcutaneous tissue oxygen tension. Anaesthesia. 2007;62(11):1101-1109.

113. Kachanov D, Filin A, Moiseenko R, et al. Cisplatin alone in therapy of standard-risk hepatoblastoma: feasibility of SIOPEL-3 SR protocol in Russia. Pediatr Blood Cancer. 2015;62:S309‐S310.

114. Kachko L, Katz J, Axer-Siegel R, et al. Sub-Tenon's ropivacaine block for pain relief after primary strabismus surgery. Curr Eye Res. 2010;35(6):529-535.

115. Kadowaki T, Isendahl J, Khalid U, et al. Semaglutide once a week in adults with overweight or obesity, with or without type 2 diabetes in an east Asian population (STEP 6): a randomised, double-blind, double-dummy, placebo-controlled, phase 3a trial. Lancet Diabetes Endocrinol. 2022;10(3):193‐206.

116. Kaess M, Durkee T, Brunner R, et al. Pathological Internet use among European adolescents: Psychopathology and self-destructive behaviours. Eur Child Adolesc Psychiatry 2014;23(11):1093-1102.

117. Kaess M, Koenig J, Bauer S, et al. Self-injury: treatment, Assessment, Recovery (STAR): online intervention for adolescent non-suicidal self-injury - Study protocol for a randomized controlled trial. Trials. 2019;20(425):1-10.

118. Kaess M, Ritter S, Lustig S, et al. Promoting Help-seeking using E-technology for ADolescents with mental health problems: study protocol for a randomized controlled trial within the ProHEAD Consortium. Trials. 2019;20(94):2-11.

119. Kaewpornsawan K, Khuntisuk S, Jatunarapit R. Comparison of modified posteromedial release and complete subtalar release in resistant congenital clubfoot: a randomized controlled trial. J Med Assoc Thai. 2007;90(5):936-941.

120. Kafali N, Cook B, Canino G, Alegria M. Cost-effectiveness of a randomized trial to treat depression among Latinos. J Ment Health Policy Econ. 2014;17(2):41‐50.

121. Kahaly GJ, Riedl M, König J, et al. Mycophenolate plus methylprednisolone versus methylprednisolone alone in active, moderate-to-severe Graves' orbitopathy (MINGO): a randomised, observer-masked, multicentre trial. Lancet Diabetes Endocrinol 2018;6(4):287-298.

122. Kahlon MK, Aksan N, Aubrey R, et al. Effect of layperson-delivered, empathy-focused program of telephone calls on loneliness, depression, and anxiety among adults during the COVID-19 pandemic: A randomized clinical trial. JAMA Psychiatry. 2021;78(6):616-622.

123. Kaier EE. Reaching a rural community: Feasibility of delivering a child posttraumatic nightmare treatment Via televideoconferencing. The University of Tulsa ProQuest Dissertations Publishing. 2018;79(1):10288388.

124. Kain J, Concha F, Moreno L, Leyton B. School-based obesity prevention intervention in Chilean children: effective in controlling, but not reducing obesity. J Obes. 2014;2014:618293.

125. Kain ZN, Wang SM, Mayes LC, Krivutza DM, Teague BA. Sensory stimuli and anxiety in children undergoing surgery: a randomized, controlled trial. Anesth Analg. 2001;92(4):897-903.

126. Kainz JT, Kozel G, Haidvogl M, Smolle J. Homoeopathic versus placebo therapy of children with warts on the hands: a randomized, double-blind clinical trial. Dermatology. 1996;193(4):318‐320.

127. Kaiser HB, Banov CH, Berkowitz RR, et al. Comparative efficacy and safety of once-daily versus twice-daily loratadine-pseudoephedrine combinations versus placebo in seasonal allergic rhinitis. Am J Ther. 1998;5(4):245‐251.

128. Kaiser HB, Findlay SR, Georgitis JW, et al. The anticholinergic agent, ipratropium bromide, is useful in the treatment of rhinorrhea associated with perennial allergic rhinitis. Allergy Asthma Proc. 1998;19(1):23-29.

129. Kaiser HB, Gopalan G, Chung W. Loratadine provides early symptom control in seasonal allergic rhinitis. Allergy Asthma Proc. 2008;29(6):654-658.

130. Kaiser L, Henry D, Flack NP, Keene O, Hayden FG. Short-term treatment with zanamivir to prevent influenza: results of a placebo-controlled study. Clin Infect Dis. 2000;30(3):587-589.

131. Kakoschke N, Hawker C, Castine B, de Courten B, Verdejo-Garcia A. Smartphone-based cognitive bias modification training improves healthy food choice in obesity: a pilot study. Eur Eat Disord Rev. 2018;26(5):526‐532.

132. Kandola A, del Pozo Cruz B, Hayes JF, Owen N, Dunstan DW, Hallgren M. Impact on adolescent mental health of replacing screen-use with exercise: A prospective cohort study. J Affect Disord. 2022;301:240-247.

133. Katapally TR, Laxer RE, Qian W, Leatherdale ST. Do school physical activity policies and programs have a role in decreasing multiple screen time behaviours among youth? Prev Med. 2018;110:106-113.

134. Ke GN, Wong SF. Outcome of the psychological intervention program: Internet use for youth. J Ration - Emot Cogn. 2018;36(2):187-200.

135. Kerr DA, Pollard CM, Howat P, et al. Connecting Health and Technology (CHAT): protocol of a randomized controlled trial to improve nutrition behaviours using mobile devices and tailored text messaging in young adults. BMC public health. 2012;12(477):1-10.

136. Khan A, Burton NW. Screen-based behaviors of adolescents in Bangladesh. J Phys Act Health. 2016;13(11):1156-1163.

137. Kobel S, Wartha O, Lämmle C, Dreyhaupt J, Steinacker JM. Intervention effects of a kindergarten-based health promotion programme on obesity related behavioural outcomes and BMI percentiles. Preventive medicine reports. 2019;15:100931.

138. Koch PA, Contento IR, Gray HL, et al. Food, health, & choices: Curriculum and wellness interventions to decrease childhood obesity in fifth-graders. J Nutr Educ Behav. 2019;51(4):440-455.

139. Koezuka N, Koo M, Allison KR, et al. The relationship between sedentary activities and physical Inactivity among adolescents: Results from the Canadian community health survey. J Adolesc Health. 2006;39(4):515-522.

140. Kriemler S, Zahner L, Schindler C, et al. Effect of school based physical activity programme (KISS) on fitness and adiposity in primary schoolchildren: cluster randomised controlled trial. BMJ 2010;340:c785.

141. Kunesova M, Vignerova J, Steflova A, et al. Obesity of Czech children and adolescents: Relation to parental obesity and socioeconomic factors. J Public Health 2007;15(3):163-170.

142. Lachausse RG. My student body: effects of an internet-based prevention program to decrease obesity among college students. J Am Coll Health. 2012;60(4):324‐330.

143. Lagström H, Jokinen E, Seppänen R, et al. Nutrient intakes by young children in a prospective randomized trial of a low-saturated fat, low-cholesterol diet. The STRIP Baby Project. Special Turku coronary risk factor intervention project for babies. Arch Pediatr Adolesc Med. 1997;151(2):181-188.

144. Laska MN, Lytle LA, Nanney MS, Moe SG, Linde JA, Hannan PJ. Results of a 2-year randomized, controlled obesity prevention trial: effects on diet, activity and sleep behaviors in an at-risk young adult population. Prev Med. 2016;89:230‐236.

145. Lavrisa Z, Hristov H, Kelly B, Pravst I. Regulating children's exposure to food marketing on television: Are the restrictions during children's programmes enough? Appetite. 2020;154(104752).

146. Lazarou C, Soteriades ES. Physical activity patterns among preadolescent children in Cyprus: The CYKIDS study. J Phys Act Health. 2009;6(2):185-194.

147. LeBourgeois MK, Hale L, Chang A-M, Akacem LD, Montgomery-Downs HE, Buxton OM. Digital media and sleep in childhood and adolescence. Pediatrics. 2017;140(5):S92-S96.

148. Lee KS, Lee JK, Yeun YR. Effects of a 10-Day intensive health promotion program combining diet and physical activity on body composition, physical fitness, and blood factors of young adults: A randomized pilot study. Med Sci Monit. 2017;23:1759-1767.

149. Leena K, Tomi L, Arja R. Intensity of mobile phone use and health compromising behaviours--How is information and communication technology connected to health-related lifestyle in adolescence? J Adolesc. 2005;28(1):35-47.

150. Livingstone S, Gorzig A. When adolescents receive sexual messages on the internet: Explaining experiences of risk and harm. Comput Hum Behav. 2014;33:8-15.

151. Maddison R, Foley L, Mhurchu CN, et al. Feasibility, design and conduct of a pragmatic randomized controlled trial to reduce overweight and obesity in children: the electronic games to aid motivation to exercise (eGAME) study. BMC public health. 2009;9(146):1-9.

152. Masse LC, Vlaar J, Macdonald J, et al. Aim2Be mHealth intervention for children with overweight and obesity: study protocol for a randomized controlled trial. Trials. 2020;21(132):1-14.

153. Monroe JT, Lofgren IE, Sartini BL, Greene GW. The Green Eating Project: web-based intervention to promote environmentally conscious eating behaviours in US university students. Public Health Nutr. 2015;18(13):2368‐2378.

154. Moore JA. Examination of the effects of computer assisted mindfulness strategies with adolescents in an alternative high school setting. Dissertations. 2017;3166.

155. Morris J, Firkins A, Millings A, Mohr C, Redford P, Rowe A. Internet-delivered cognitive behavior therapy for anxiety and insomnia in a higher education context. Anxiety Stress Coping. 2016;29(4):415‐431.

156. Motamedi Heravi M, Khosravan S, Mohammadi A, Mansoorian MR. Effects of self-management training on smartphone dependence in low to moderate adolescent males' users. Behaviour Change. 2023;40(1):67-75.

157. Motl RW, McAuley E, Birnbaum AS, Lytle LA. Naturally occurring changes in time spent watching television are inversely related to frequency of physical activity during early adolescence. J Adolesc. 2006;29(1):19-32.

158. Munoz MA. The association between parent and child variables and physical activity and sedentary behaviors in Puerto Rican children. Dissertations. 2016;77(3).

159. Murray DM, Stevens J, Hannan PJ, et al. School-level intraclass correlation for physical activity in sixth grade girls. Med Sci Sports Exerc. 2006;38(5):926-936.

160. (NIDDK) NIoDaDaKD. Modifying the home television watching environment. 2020(1).

161. University S. Sports to prevent obesity: Feasibility and pilot RCT. 2020(1).

162. Patrick K. PACE-PC: Primary care management of adolescent obesity. 2018(5).

163. Institute H. Healthy Homes/Healthy Kids_5-9. 2020(1).

164. Health NL. Starting early obesity prevention program. 2018(5).

165. Patrick K. PACE+: Counseling adolescents for exercise and nutrition. 2018(5).

166. Zajac K. Reducing internet gaming. 2018;5.

167. College C. Efficacy of a comprehensive school-based intervention for high-functioning children with autism spectrum disorder. 2018(5).

168. Mendoza J. Fit 5 kids screen time reduction curriculum for Latino preschoolers. 2019(1).

169. Yang X. A cognitive behavioral therapy intervention for internet gaming disorder. 2020(2).

170. Rojas-Barahona CA. Early development of academic skills in the classroom. 2022(2).

171. Neshteruk CD, Tripicchio GL, Lobaugh S, et al. Screen time parenting practices and associations with preschool children's tv viewing and weight-related outcomes. Int J Environ Res Public Health. 2021;18(7359):1-9.

172. Nguyen P, Le LK-D, Nguyen D, Gao L, Dunstan DW, Moodie M. The effectiveness of sedentary behaviour interventions on sitting time and screen time in children and adults: An umbrella review of systematic reviews. Int J Behav Nutr Phys Act. 2020;17(117):1-11.

173. Nikolopoulou K. Preschool children's use of tablet at home and parents' views. Nikolopoulou, Kleopatra: University of Athens, Athens, Greece Hershey, PA: Information Science Reference/IGI Global; US; 2020.

174. Novotny R, Yamanaka AB, Butel J, et al. Maintenance outcomes of the children's healthy living program on overweight, obesity, and acanthosis nigricans among young children in the US-Affiliated Pacific Region: A randomized clinical trial. JAMA Network Open. 2022;5(6):e2214802.

175. Patel BP, Bellissimo N, Thomas SG, Hamilton JK, Anderson GH. Television viewing at mealtime reduces caloric compensation in peripubertal, but not postpubertal, girls. Pediatr Res. 2011;70(5):513‐517.

176. Patrick K, Norman GJ, Calfas KJ, et al. Diet, physical activity, and sedentary behaviors as risk factors for overweight in adolescence. Arch Pediatr Adolesc Med. 2004;158(4):385‐390.

177. Pedersen J, Rasmussen MGB, Sørensen SO, et al. Effects of limiting recreational screen media use on physical activity and sleep in families with children: A cluster randomized clinical trial. JAMA Pediatr. 2022;176(8):741-749.

178. Piotrowski JT, Jordan AB, Bleakley A, Hennessy M. Identifying family television practices to reduce children's television time. J Fam Commun. 2015;15(2):159-174.

179. Raat H, Struijk MK, Remmers T, et al. Primary prevention of overweight in preschool children, the BeeBOFT study (breastfeeding, breakfast daily, outside playing, few sweet drinks, less TV viewing): design of a cluster randomized controlled trial. BMC public health. 2013;13(974):1-11.

180. Radhakrishna S, Srinivasan I, Setty JV, D R MK, Melwani A, Hegde KM. Comparison of three behavior modification techniques for management of anxious children aged 4–8 years. J Dent Anesth Pain Med. 2019;19(1):29‐36.

181. Rasmussen MGB, Pedersen J, Olesen LG, et al. Short-term efficacy of reducing screen media use on physical activity, sleep, and physiological stress in families with children aged 4-14: study protocol for the SCREENS randomized controlled trial. BMC public health. 2020;20(380):1-18.

182. Rasmussen MGB, Pedersen J, Olesen LG, Kristensen PL, Brønd JC, Grøntved A. Feasibility of two screen media reduction interventions: results from the SCREENS pilot trial. PLoS One. 2021;16(11):e0259657.

183. Raynor HA, Osterholt KM, Hart CN, Jelalian E, Vivier P, Wing RR. Efficacy of U.S. paediatric obesity primary care guidelines: two randomized trials. Pediatr Obes. 2012;7(1):28‐38.

184. Resnicow K, McMaster F, Woolford S, et al. Study design and baseline description of the BMI2 trial: reducing paediatric obesity in primary care practices. Pediatr Obes. 2012;7(1):3‐15.

185. Richardson L, Parker EO, Zhou C, Kientz J, Ozer E, McCarty C. Electronic health risk behavior screening with integrated feedback among adolescents in primary care: Randomized controlled trial. J Med Internet Res. 2021;23(3):e24135.

186. Robidoux H, Ellington E, Lauerer J. Screen time: The impact of digital technology on children and strategies in care. J Psychosoc Nurs Ment Health Serv. 2019;57(11):15-20.

187. Robinson TN, Banda JA, Hale L, et al. Screen media exposure and obesity in children and adolescents. Pediatrics. 2017;140(5):S97-S101.

188. Robinson TN, Kraemer HC, Matheson DM, et al. Stanford GEMS phase 2 obesity prevention trial for low-income African-American girls: design and sample baseline characteristics. Contemp Clin Trials. 2008;29(1):56‐69.

189. Robinson TN, Matheson DM. Environmental interventions to reduce overeating in children. Robinson, Thomas N.: Division of General Pediatrics and Stanford Prevention Research Center, Center for Healthy Weight, Stanford University School of Medicine, Stanford, CA, US Matheson, Donna M.: Stanford Prevention Research Center, Stanford University School of Medicine, Stanford, CA, US New York, NY: Oxford University Press; US; 2012.

190. Robinson TN, Matheson DM. Environmental strategies for portion control in children. Appetite. 2015;88:33-38.

191. Roemmich JN, Gurgol CM, Epstein LH. Open-loop feedback increases physical activity of youth. Med Sci Sports Exerc. 2004;36(4):668‐673.

192. Roemmich JN, Lobarinas CL, Barkley JE, White TM, Paluch R, Epstein LH. Use of an open-loop system to increase physical activity. Pediatr Exerc Sci. 2012;24(3):384‐398.

193. Roman-Juan J, Roy R, Jensen MP, Miro J. The explanatory role of sedentary screen time and obesity in the increase of chronic back pain amongst european adolescents: The hbsc study 2002-2014. Eur J Pain. 2022;26(8):1781-1789.

194. Ronniger P, Melzer J, Petermann F, Rennecke L. Resources in television consumption: Effects of parental supervision. Zeitschrift für Psychiatr Psychol und Psychother. 2019;67(3):165-172.

195. Rosenkoetter LI, Rosenkoetter SE, Acock AC. Television violence: An intervention to reduce its impact on children. J Appl Dev Psychol. 2009;30(4):381-397.

196. Royant-Parola S, Londe V, Trehout S, Hartley S. The use of social media modifies teenagers' sleep-related behavior. Encephale. 2018;44(4):321-328.

197. Rushovich BR, Voorhees CC, Davis C, et al. The relationship between unsupervised time after school and physical activity in adolescent girls. Int J Behav Nutr Phys Act. 2006;3(20):1-18.

198. Russell C, Buhrau D, Hamby A. Reducing television influences on U.S. adolescents who are high reactance. J Child Media. 2021;15(2):153-164.

199. Sacheck JM, Wright CM, Amin SA, et al. The Fueling Learning Through Exercise Study Cluster RCT: Impact on Children's Moderate-to-Vigorous Physical Activity. J Pediatr. 2021;60(6):e239-e249.

200. Sacher PM, Kolotourou M, Chadwick PM, et al. Randomized controlled trial of the MEND program: a family-based community intervention for childhood obesity. Obesity (Silver Spring). 2010;18:S62-68.

201. Salmon J, Hume C, Ball K, Booth M, Crawford D. Individual, social and home environment determinants of change in children's television viewing: the Switch-Play intervention. J Sci Med Sport. 2006;9(5):378‐387.

202. Sanders W, Parent J, Forehand R. Parenting to Reduce Child Screen Time: a Feasibility Pilot Study. J Child Fam Stud. 2018;39(1):46‐54.

203. Schilder J, Brusselaers M, Bogaerts S. The Effectiveness of an Intervention to Promote Awareness and Reduce Online Risk Behavior in Early Adolescence. J Clin Med. 2016;45(2):286‐300.

204. Schleider JL, Dobias M, Fassler J, Shroff A, Pat S. Promoting treatment access following pediatric primary care depression screening: randomized trial of web-based, single-session interventions for parents and youths. J Am Acad Child Adolesc Psychiatry. 2020;59(6):770-773.

205. Schneider M, Dunton GF, Cooper DM. Media use and obesity in adolescent females. Obesity (Silver Spring). 2007;15(9):2328-2335.

206. Schwinn TM, Schinke S, Fang L, Kandasamy S. A web-based, health promotion program for adolescent girls and their mothers who reside in public housing. Health Educ Behav. 2014;39(4):757‐760.

207. Secades-Villa R, Calafat A, Fernandez-Hermida JR, et al. Duration of Internet use and adverse psychosocial effects among European adolescents. Adicciones. 2014;26(3):247-253.

208. Shelton D, Le Gros K, Norton L, Stanton-Cook S, Morgan J, Masterman P. Randomised controlled trial: a parent-based group education programme for overweight children. J Paediatr Child Health. 2007;43(12):799‐805.

209. Shriberg LD, Strand EA, Jakielski KJ, Mabie HL. Estimates of the prevalence of speech and motor speech disorders in persons with complex neurodevelopmental disorders. J Speech Lang Hear Res. 2019;33(8):707-736.

210. Siegrist M, Lammel C, Haller B, Christle J, Halle M. Effects of a physical education program on physical activity, fitness, and health in children: the JuvenTUM project. Scand J Med Sci Sports. 2013;23(3):323-330.

211. Sigmund E, El Ansari W, Sigmundová D. Does school-based physical activity decrease overweight and obesity in children aged 6-9 years? A two-year non-randomized longitudinal intervention study in the Czech Republic. BMC Public Health. 2012;12:570.

212. Sigmund E, Sigmundova D, Badura P. Excessive body weight of children and adolescents in the spotlight of their parents' overweight and obesity, physical activity, and screen time. Int J Environ Res Public Health. 2020;65(8):1309-1317.

213. Staiano AE, Beyl RA, Guan W, Hendrick CA, Hsia DS, Newton RL. Home-based exergaming among children with overweight and obesity: a randomized clinical trial. Pediatr Obes. 2018;13(11):724‐733.

214. Straker L, Abbott R, Mathiassen S. Teaching our children to sit or be active? Sedentary behavior, light activity and moderate/vigorous activity at and away from school. J Sci Med Sport. 2012;15:S280‐.

215. Straker LM, Abbott RA, Piek JP, Pollock CM, Davies PS, Smith AJ. Rationale, design and methods for a randomised and controlled trial to investigate whether home access to electronic games decreases children's physical activity. BMC Public Health. 2009;9:212.

216. Strasburger VC. Policy statement-media education. American Academy of Pediatrics. Pediatrics. 2010;126(5):1012-1017.

217. Suchert V, Hanewinkel R, Isensee B. Screen time, weight status and the self-concept of physical attractiveness in adolescents. J Adolesc. 2016;48:11-17.

218. Temple JL, Giacomelli AM, Kent KM, Roemmich JN, Epstein LH. Television watching increases motivated responding for food and energy intake in children. Am J Clin Nutr. 2007;85(2):355‐361.

219. Thomas G, Bennie JA, De Cocker K, Ireland MJ, Biddle SJ. Screen-based behaviors in Australian adolescents: Longitudinal trends from a 4-year follow-up study. PLoS One. 2020;141.

220. Todd MK, Reis-Bergan MJ, Sidman CL, et al. Effect of a family-based intervention on electronic media use and body composition among boys aged 8--11 years: a pilot study. J Prim Prev. 2008;12(4):344‐358.

221. Tomayko EJ, Prince RJ, Cronin KA, Adams AK. The Healthy Children, Strong Families intervention promotes improvements in nutrition, activity and body weight in American Indian families with young children. J Obes. 2016;19(15):2850‐2859.

222. Torres-Rodriguez A, Griffiths MD, Carbonell X. The treatment of Internet gaming disorder: A brief overview of the PIPATIC program. Int J Ment Health Addict. 2018;16(4):1000-1015.

223. Torres-Rodriguez A, Griffiths MD, Carbonell X, Oberst U. Treatment efficacy of a specialized psychotherapy program for Internet Gaming Disorder. J Behav Addict. 2018;7(4):939‐952.

224. Tremblay MS, LeBlanc AG, Kho ME, et al. Systematic review of sedentary behaviour and health indicators in school-aged children and youth. Int J Behav Nutr Phys Act. 2011;8.

225. Tripathi M, Mishra SK. Screen time and adiposity among children and adolescents: A systematic review. J Public Health (Oxf). 2020;28(3):227-244.

226. Tully L, Sorensen J, O'Malley G. Pediatric Weight Management Through mHealth Compared to Face-to-Face Care: cost Analysis of a Randomized Control Trial. JMIR Mhealth Uhealth. 2021;9(9):e31621.

227. Turel O, Bechara A. Little video-gaming in adolescents can be protective, but too much is associated with increased substance use. Psychol Addict Behav. 2019;54(3):384-395.

228. Van den Bulck J. Television viewing, computer game playing, and Internet use and self-reported time to bed and time out of bed in secondary-school children. Sleep. 2004;27(1):101-104.

229. Van den Bulck J, Eggermont S. Media use as a reason for meal skipping and fast eating in secondary school children. J Hum Nutr Diet. 2006;19(2):91-100.

230. Van den Bulck J, Hofman A. The television-to-exercise ratio is a predictor of overweight in adolescents: Results from a prospective cohort study with a two year follow up. Prev Med. 2009;48(4):368-371.

231. van Grieken A, Ezendam NP, Paulis WD, van der Wouden JC, Raat H. Primary prevention of overweight in children and adolescents: A meta-analysis of the effectiveness of interventions aiming to decrease sedentary behaviour. Int J Behav Nutr Phys Act. 2012;9.

232. van Otterloo SG, van der Leij A, Henrichs LF. Early home-based intervention in the Netherlands for children at familial risk of dyslexia. Dyslexia. 2009;15(3):187-217.

233. Varagiannis P, Magriplis E, Risvas G, et al. Effects of Three Different Family-Based Interventions in Overweight and Obese Children: the "4 Your Family" Randomized Controlled Trial. Child Obes. 2021;13(2).

234. Vasconcellos F, Seabra A, Cunha F, et al. Health markers in obese adolescents improved by a 12-week recreational soccer program: a randomised controlled trial. J Sports Sci. 2016;34(6):564-575.

235. Verbeken S, Braet C, Goossens L, Van Der Oord S. Executive function training with game elements for obese children: a novel treatment to enhance self-regulatory abilities for weight-control. Behav Res Ther. 2013;6:40.

236. Weber D, Rissel C, Hector D, Wen LM. Supported playgroups as a setting for promoting physical activity of young children: Findings from a feasibility study in south-west Sydney, Australia. J Paediatr Child Health. 2014;50(4):301-305.

237. Weintraub DL, Tirumalai EC, Haydel KF, Fujimoto M, Fulton JE, Robinson TN. Team sports for overweight children: the Stanford Sports to Prevent Obesity Randomized Trial (SPORT). Arch Pediatr Adolesc Med. 2008;162(3):232‐237.

238. Wengle JG, Hamilton JK, Manlhiot C, et al. The 'Golden Keys' to health - A healthy lifestyle intervention with randomized individual mentorship for overweight and obesity in adolescents. Paediatr Child Health. 2011;16(8):473‐478.

239. Williamson DA, Champagne CM, Harsha DW, et al. Effect of an environmental school-based obesity prevention program on changes in body fat and body weight: a randomized trial. Obesity (Silver Spring). 2012;20(8):1653‐1661.

240. Williamson DA, Walden HM, White MA, et al. Two-year internet-based randomized controlled trial for weight loss in African-American girls. Obesity (Silver Spring). 2006;14(7):1231‐1243.

241. Willson JM. An exploration of preschool and early school age children's patterns of television viewing and reading behaviour in the longitudinal study of Australian children. Early Child Dev Care. 2022;83(5):No Pagination Specified.

242. Wilson DK, Sweeney AM, Law LH, Kitzman-Ulrich H, Resnicow K. Web-Based Program Exposure and Retention in the Families Improving Together for Weight Loss Trial. Child Obes. 2019;53(4):399‐404.

243. Wofford L, Froeber D, Clinton B, Ruchman E. Free afterschool program for at-risk African American children findings and lessons. J Community Psychol. 2013;36(4):299-310.

244. Wolfe DA, Mendes MG, Factor D. A parent-administered program to reduce children's television viewing. J Appl Behav Anal. 1984;17(2):267-272.

245. Wood KR, Wood E, Gottardo A, Archer K, Savage R, Piquette N. Workshop training to facilitate parent-child instructional opportunities for reading and social development with kindergarten students. Early Child Dev Care. 2020:No Pagination Specified.

246. Xu F-Z, Zhang W-X. Relationship between adolescents' alienation and pathological Internet use: Testing the moderating effect of family functioning and peer acceptance. Psychol Health Med. 2018;43(4):410-419.

247. Yang Y, Liu R-D, Liu J, Ding Y, Hong W, Jiang S. The relations between parental active mediation, parent-child relationships and children's problematic mobile phone use: A longitudinal study. Comput Hum Behav. 2021:No Pagination Specified.

248. Yu JJ, Kim H, Hay I. Understanding adolescents' problematic internet use from a social/cognitive and addiction research framework. Comput Hum Behav. 2013;29(6):2682-2689.

249. ChiCtr. The B.E.S.T. Comprehensive Intervention Technology in the Treatment of Adolescents with Mobile Game Addiction. https://trialsearchwhoint/Trial2aspx?TrialID=ChiCTR2200063673. 2022.

250. Dickinson K, Place M. A Randomised Control Trial of the Impact of a Computer-Based Activity Programme upon the Fitness of Children with Autism. Autism research and treatment. 2014;2014:419653.

251. Djamnezhad D, Bergström M, Andrén P, Hofvander B. Good behavior game - study protocol for a randomized controlled trial of a preventive behavior management program in a Swedish school context. Frontiers in psychiatry. 2023;14:1256714.

252. Irct20230102057027N. comparison of the mindfulness-based cognitive therapy (MBCT) and acceptance and commitment therapy (ACT) on reducing of anxiety and depression in adolescents with Internet addiction. https://trialsearchwhoint/Trial2aspx?TrialID=IRCT20230102057027N1. 2023.

253. Nct. A Co-creational Intervention to Engage Preschoolers in Healthy Movement Behaviors. https://clinicaltrialsgov/ct2/show/NCT06073236. 2022.

254. Raj D, Ahmad N, Mohd Zulkefli NA, Lim PY. Stop and Play Digital Health Education Intervention for Reducing Excessive Screen Time Among Preschoolers From Low Socioeconomic Families: Cluster Randomized Controlled Trial. Journal of medical Internet research. 2023;25:e40955.

255. Tugault-Lafleur CN, De-Jongh González O, Macdonald J, Bradbury J, Warshawski T, Ball GDC, et al. Efficacy of the Aim2Be Intervention in Changing Lifestyle Behaviors Among Adolescents With Overweight and Obesity: Randomized Controlled Trial. Journal of medical Internet research. 2023;25:e38545.

256. Vilardell-Dávila A, Martínez-Andrade G, Klünder-Klünder M, Miranda-Lora AL, Mendoza E, Flores-Huerta S, et al. A Multi-Component Educational Intervention for Addressing Levels of Physical Activity and Sedentary Behaviors of Schoolchildren. International journal of environmental research and public health. 2023;20(4).

257. Dickinson K, Place M. A Randomised Control Trial of the Impact of a Computer-Based Activity Programme upon the Fitness of Children with Autism. Autism research and treatment. 2014;2014:419653.

258. Djamnezhad D, Bergström M, Andrén P, Hofvander B. Good behavior game - study protocol for a randomized controlled trial of a preventive behavior management program in a Swedish school context. Frontiers in psychiatry. 2023;14:1256714.

259. Irct20230102057027N. comparison of the mindfulness-based cognitive therapy (MBCT) and acceptance and commitment therapy (ACT) on reducing of anxiety and depression in adolescents with Internet addiction. https://trialsearchwhoint/Trial2aspx?TrialID=IRCT20230102057027N1. 2023.

260. Nct. A Co-creational Intervention to Engage Preschoolers in Healthy Movement Behaviors. https://clinicaltrialsgov/ct2/show/NCT06073236. 2022.

261. Nct. Personalising Children's Screen Use Reduction for Better Sleep, Mental, and Brain Health. https://clinicaltrialsgov/ct2/show/NCT05956392. 2023.

262. Raj D, Ahmad N, Mohd Zulkefli NA, Lim PY. Stop and Play Digital Health Education Intervention for Reducing Excessive Screen Time Among Preschoolers From Low Socioeconomic Families: Cluster Randomized Controlled Trial. Journal of medical Internet research. 2023;25:e40955.

263. Tugault-Lafleur CN, De-Jongh González O, Macdonald J, Bradbury J, Warshawski T, Ball GDC, et al. Efficacy of the Aim2Be Intervention in Changing Lifestyle Behaviors Among Adolescents With Overweight and Obesity: Randomized Controlled Trial. Journal of medical Internet research. 2023;25:e38545.

264. Vilardell-Dávila A, Martínez-Andrade G, Klünder-Klünder M, Miranda-Lora AL, Mendoza E, Flores-Huerta S, et al. A Multi-Component Educational Intervention for Addressing Levels of Physical Activity and Sedentary Behaviors of Schoolchildren. International journal of environmental research and public health. 2023;20(4).

265. Wang A, Gao Y, Wang J, Tong TK, Sun Y, Yu S, et al. Effects of a School-Based Physical Activity Intervention for Obesity and Health-Related Physical Fitness in Adolescents With Intellectual Disability: Protocol for a Randomized Controlled Trial. JMIR research protocols. 2021;10(3):e25838.

266. Aulbach MB, Puukko S, Palsola M, Haukkala A, Sund R, Vasankari T, et al. How does a school-based intervention impact students' social cognitions on reducing sedentary behavior over 14 months? Psychology, health & medicine. 2024;29(7):1235-1249.

267. Bourke M, Bruijns BA, Vanderloo LM, Irwin J, Heydon R, Carson V, et al. The efficacy of the TEACH e-Learning course at improving early childhood educators’ physical activity and sedentary behaviour self-efficacy, knowledge, intentions, and perceived behavioural control: a randomized controlled trial. International journal of behavioral nutrition and physical activity. 2024;21(1).

268. Collin AW, Diana M, Anthony DM, Ivana B, Dušan P, Dragan S, et al. Student Profiles of Physical Activity, Screen Time, Sleep Quality and Dietary Habits and Their Association with Mental Health and School Satisfaction: An Exploratory Study. 2024;61(4):1667-1693.

269. Davis CG, Goldfield GS. Limiting social media use decreases depression, anxiety, and fear of missing out in youth with emotional distress: A randomized controlled trial. US: Educational Publishing Foundation; 2024. p. No Pagination Specified-No Pagination Specified.

270. Duarte A, Martins J, Magalhaes F, Augusto C, Silva MJ, Martins S, et al. Is the use of screens during mealtime associated with the waist-to-weight ratio of school-aged children. Obesity facts. 2024;17:252‐253.

271. Justin AH, Fenghua S, Chunxiao L, Kwok N, Jihyun L, Stefanie Hwee Chee A, et al. Environmental Correlates of Physical Activity and Screen-Time in Youth with Autism Spectrum Disorder: A Seven-Country Observational Study. 2024;54(5):1740-1748.

272. Madison P, Valerie C. Screen Time Policy in Alberta Childcare Centres. 2024;52(1):13-20.

273. Maree H, Shahid AA. Parents' Knowledge, Perceptions and Support around Appropriate Physical Activity, Screen Time and Sleep Time Levels for Children.18.

274. Schmidt-Persson J, Rasmussen MGB, Sørensen SO, Mortensen SR, Olesen LG, Brage S, et al. Screen Media Use and Mental Health of Children and Adolescents: A Secondary Analysis of a Randomized Clinical Trial. JAMA network open. 2024;7(7):e2419881.

**Appendix S5.** Results: exploration of heterogeneity.

**Results: exploration of heterogeneity**

**Primary outcomes**

**Screen or sedentary time (variously measured)**

There was substantial heterogeneity for this outcome, as indicated by an I^2^ of 68%. We explored possible factors that could have accounted for the heterogeneity, including subgrouping studies that evaluated screen-time focussed interventions (eight studies) and those that evaluated a comprehensive lifestyle intervention (17 studies). We found that separation into these subgroups did not substantially reduce the I^2^ value, with no clear differences in the effect sizes between subgroups (p=0.06). Additionally, there was no clear trend in the effect sizes according to sample size, as reported by a previous systematic review^1^. Consequently, we accepted the pooled estimates while downgraded the certainty-of-evidence in the domain of inconsistency due to unexplained heterogeneity.

For the outcome of the number of participants who met screen time expectations, there was very large degree of heterogeneity as indicated by the I^2^ of 94%. We could not identify a plausible explanation for the very high degree heterogeneity in terms of study design, population, intervention, comparison and outcome measurement. We therefore accepted the pooled estimate with downgraded certainty-of- evidence.

**Physical activity**

For this outcome, I^2^ of 88%. Indicated substantial heterogeneity. We explored possible explanatory factors including subgrouping studies that evaluated screen-time focussed interventions (four studies) and those that evaluated a comprehensive lifestyle intervention (16 studies). We found that separation into subgroups did not substantially reduce the I^2^ value (test for subgroup differences: p=0.20). Additionally, there was no clear trend in the effect sizes according to sample size. Consequently, we downgraded the certainty-of-evidence in the domain of inconsistency due to unexplained heterogeneity.

For the outcome of the number of participants who met physical activity expectations, there was a very large degree of heterogeneity, as indicated by the I^2^ of 82%. We could not identify a plausible explanation for the very high degree heterogeneity in terms of study design, population, intervention, comparison and outcome measurement. We therefore accepted the pooled estimate with downgraded certainty of the evidence. The contradictory findings between individual studies – all of which show clear beneficial effect and the pooled estimate, which shows non-significant effect, was the result of the choice of non-informative prior in the Bayesian analysis, which presumes the mean of a zero effect size and SD of 0.707 logOR (equivalent to OR of 2.03)).

**Secondary outcomes**

**BMI**

There was moderate-to-substantial heterogeneity for this outcome, as indicated by an I^2^ of 51%. We explored possible factors that could have accounted for the variability in the effect sizes of the studies, including the conduct of a subgroup analysis separating studies that evaluated screen-time focussed interventions (five studies, n=2112) and those that evaluated a comprehensive lifestyle intervention (eight studies, n=2571).We found a significant difference in the pooled effect estimates between the two subgroups (test for subgroup differences: p=0.01), as the screen-time focused intervention subgroup showed no clear differences in BMI (MD -0.1, 95%CrI: -0.17,0.23) (eFigure 5b) while the lifestyle intervention subgroup showed a modest but significant difference in BMI favouring the intervention group (MD -0.35, 95%CrI -0.79,-0.05) (eFigure 5c). However, separation into subgroups of screen time-focused versus lifestyle intervention reduced the I^2^ value of screen-time focused subgroup to 18%, it increased the value of lifestyle intervention subgroup to 60%. Additionally, there was no clear trend in the effect sizes according to sample size, as reported by a previous systematic review^1^. Consequently, we considered the nature of the intervention (screen-time focused versus lifestyle intervention) only partially explanatory for the degree of heterogeneity observed, and downgraded the certainty-of-evidence in the domain of inconsistency due to unexplained heterogeneity.

**Mental health-related outcomes**

In both outcomes under this category (severity of gaming or internet addiction disorder and self-efficacy and well-being), there was substantial heterogeneity as indicated by the I^2^ of over 90%. Upon initial assessment, much of the heterogeneity was contributed by the markedly different results between two studies (Agbaria 2022 and Bagherniya 2018)^2,3^ and the remaining studies. We explored possible sources of heterogeneity in terms of study design, population, intervention, comparison and outcome measurement. While we found study design as plausible factor for Agbaria 2022 (as it was an individual RCT vs others that were cluster RCTs), it was not a plausible factor for Bagherniya 2018 as it was a cluster RCT like all the rest of the study. One other plausible explanatory factor was the participant characteristics, as both Agbaria 2022 and Bagherniya 2018 enrolled participants with risk factors (Agbaria 2022 enrolled participants at risk of internet addiction and Bagherniya 2018 enrolled overweight or obese participants, while most of the other studies enrolled general student population. However, we found participant characteristics not a completely convincing explanatory factor, as one other included study Lindenberg 2022^4^ also enrolled at-risk participants (those who were at high risk of internet addiction disorder) but the finding of this study did not differ substantially from the remaining studies that enrolled general populations. There is no other acceptable explanation that could convincingly account for the heterogeneity. As such, we considered our postulation of participant characteristics as a possible contributory factor in the heterogeneity of study results at best preliminary, and accepted the pooled findings with downgraded certainty of the evidence.

**References**

1. Jones A, Armstrong B, Weaver RG, Parker H, von Klinggraeff L, Beets MW. Identifying effective intervention strategies to reduce children’s screen time: a systematic review and meta-analysis. *International Journal of Behavioral Nutrition and Physical Activity.* 2021;18(1):126.

2. Agbaria Q. Cognitive behavioral intervention in dealing with Internet addiction among Arab teenagers in Israel. *International Journal of Mental Health and Addiction.* 2023;21(4):2493-2507.

3. Bagherniya M, Mostafavi Darani F, Sharma M, et al. Assessment of the Efficacy of Physical Activity Level and Lifestyle Behavior Interventions Applying Social Cognitive Theory for Overweight and Obese Girl Adolescents. *Journal of research in health sciences.* 2018;18(2):e00409.

4. Lindenberg K, Kindt S, Szász-Janocha C. Effectiveness of Cognitive Behavioral Therapy-Based Intervention in Preventing Gaming Disorder and Unspecified Internet Use Disorder in Adolescents: A Cluster Randomized Clinical Trial. *JAMA network open.* 2022;5(2):e2148995.

**Appendix S6.** Summary of findings table with ratings of certainty-of-evidence

|  | | | | | | |
| --- | --- | --- | --- | --- | --- | --- |
| **School-based intervention for reducing screen time: a meta-analysis of randomised controlled trials** | | | | | | |
| **Patient or population:** reducing screen time and increasing physical activities among school children  **Setting:** Schools  **Intervention:** school-based interventions with screen time reduction as one of the components  **Comparison:** control (standard curriculum or no active intervention) | | | | | | |
| Outcomes | **Anticipated absolute effects^*^** (95% CrI) | | Relative effect (95% CrI) | № of participants (studies) | Certainty of the evidence (GRADE) | Comments |
|  | **Risk with control** | **Risk with school-based interventions with screen time reduction as one of the main components** |  |  |  |  |
| Screen or sedentary time (variously measured) (Screen time) assessed with: Mean difference in screen time (min) per week, per day, or block of screen or sedentary time per day. | - | SMD **0.10 SD lower** (0.14 lower to 0.06 lower) | - | 19751 (27 trials (RCTs and cluster RCTs)) | ⨁⨁◯◯ Low^a,b^ | School-based screen-time reduction interventions may result in a modest reduction in screen or sedentary time (variously measured). |
| Academic performance |  |  | No studies assessed the outcome | | | |
| Amount of physical activities (variously measured) (Physical activities ) assessed with: variously measured: time in moderate or vigorous physical activities, block of time in moderate to vigorous physical activities. | - | SMD **0.10 SD higher** (0.02 higher to 0.19 higher) | - | 14944 (21 trials (RCTs and cluster RCTs)) | ⨁⨁◯◯ Low^a,b^ | School-based screen-time reduction interventions may result in a modest increase in the amount of physical activities (variously measured). |
| Body mass index (BMI): overall pooled estimate | The median body mass index was **21.2** kg/M^2^ | MD **0.15 kg/M^2^ lower** (0.39 lower to 0.03 higher) | - | 4683 (13 trials (RCTs and cluster RCTs)) | ⨁⨁◯◯ Low^a,b^ | School-based screen-time reduction interventions may result in little to no difference in BMI. |
| Body mass index (BMI): subgroup of studies with screen-time focused intervention | The median body mass index was **20.3** kg/M^2^ | MD **0.1 kg/M^2^ lower** (0.17 lower to 0.23 higher) | - | 2112 (5 cluster RCTs) | ⨁⨁◯◯ Low^a,b^ | School-based interventions that focus on screen time reduction may result in little to no difference in BMI. |
| Body mass index (BMI):  subgroup of studies with comprehensive lifestyle intervention including a screen time component | The median body mass index was **21.1** kg/M^2^ | MD **0.35 kg/M^2^ lower** (0.78 lower to 0.05 lower) | - | 2571 (8 trials (RCTs and cluster RCTs)) | ⨁⨁◯◯ Low^a,c^ | School-based interventions that compose of comprehensive lifestyle interventions with a screen time reduction component may result in slight reduction in BMI. |
| ***The risk in the intervention group** (and its 95% confidence interval) is based on the assumed risk in the comparison group and the **relative effect** of the intervention (and its 95% CI).  **CI:** confidence interval; **MD:** mean difference; **SMD:** standardised mean difference | | | | | | |
| **GRADE Working Group grades of evidence** **High certainty:** we are very confident that the true effect lies close to that of the estimate of the effect. **Moderate certainty:** we are moderately confident in the effect estimate: the true effect is likely to be close to the estimate of the effect, but there is a possibility that it is substantially different. **Low certainty:** our confidence in the effect estimate is limited: the true effect may be substantially different from the estimate of the effect. **Very low certainty:** we have very little confidence in the effect estimate: the true effect is likely to be substantially different from the estimate of effect. | | | | | | |

#### Explanations

1. More than half of the included studies were judged to have an overall high risk-of-bias.
2. There is substantial heterogeneity among the included studies which was not sufficiently explained by the major study characteristics in population, intervention, comparison and outcome measurements.

The estimate is imprecise as reflected by the width of the 95% CrI, ranging from a moderate to a very slight reduction in BMI.

**Appendix S7.** Citations of published systematic reviews that evaluated interventions to reduce screen time for children

1. Friedrich RR, Polet JP, Schuch I, Wagner MB. Effect of intervention programs in schools to reduce screen time: a meta-analysis. *J Pediatr (Rio J).* 2014;90(3):232-241.

2. Jones A, Armstrong B, Weaver RG, Parker H, von Klinggraeff L, Beets MW. Identifying effective intervention strategies to reduce children’s screen time: a systematic review and meta-analysis. *International Journal of Behavioral Nutrition and Physical Activity.* 2021;18(1):126.

3. Krafft H, Boehm K, Schwarz S, Eichinger M, Büssing A, Martin D. Media Awareness and Screen Time Reduction in Children, Youth or Families: A Systematic Literature Review. *Child Psychiatry & Human Development.* 2023;54(3):815-825.

4. Liu Z, Xu HM, Wen LM, et al. A systematic review and meta-analysis of the overall effects of school-based obesity prevention interventions and effect differences by intervention components. *The international journal of behavioral nutrition and physical activity.* 2019;16(1):95.

5. Maniccia DM, Davison KK, Marshall SJ, Manganello JA, Dennison BA. A meta-analysis of interventions that target children's screen time for reduction. *Pediatrics.* 2011;128(1):e193-210.

6. Marsh S, Foley LS, Wilks DC, Maddison R. Family-based interventions for reducing sedentary time in youth: a systematic review of randomized controlled trials. *Obesity reviews : an official journal of the International Association for the Study of Obesity.* 2014;15(2):117-133.

7. Martin K. *Interventions to control children’s screen use and their effect on sleep: a systematic review and meta-analysis* Adelaide, Australia School of Public Health Faculty of Health and Medical Sciences The University of Adelaide 2020.

8. Raj D, Mohd Zulkefli NA, Minhat HS, N. A. Parental Intervention Strategies to Reduce Screen Time Among Preschool-aged Children: A Systematic Review *Malaysian Journal of Medicine and Health Sciences.* 2022;18(6):295-304.

9. Schmidt ME, Haines J, O'Brien A, et al. Systematic review of effective strategies for reducing screen time among young children. *Obesity (Silver Spring, Md).* 2012;20(7):1338-1354.

10. Wahi G, Parkin PC, Beyene J, Uleryk EM, Birken CS. Effectiveness of interventions aimed at reducing screen time in children: a systematic review and meta-analysis of randomized controlled trials. *Archives of pediatrics & adolescent medicine.* 2011;165(11):979-986.

11. Wu L, Sun S, He Y, Jiang B. The effect of interventions targeting screen time reduction: A systematic review and meta-analysis. *Medicine.* 2016;95(27):e4029.

12. Zhang P, Tang X, Peng X, Hao G, Luo S, Liang X. Effect of screen time intervention on obesity among children and adolescent: A meta-analysis of randomized controlled studies. *Preventive medicine.* 2022;157:107014.

**Figure S1.** Funnel plot screen time.


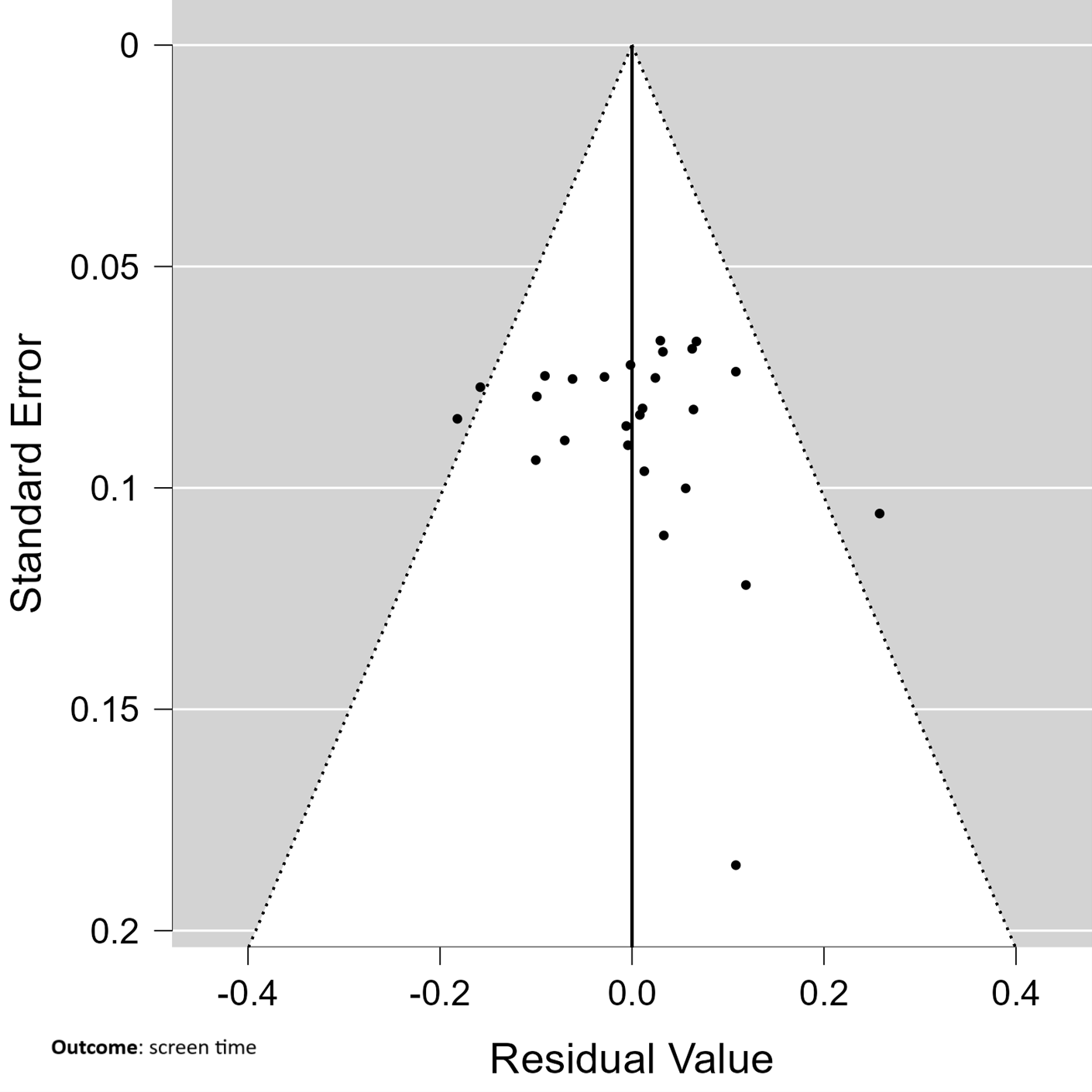


**Figure S2.** Forest plot screen time expectation.


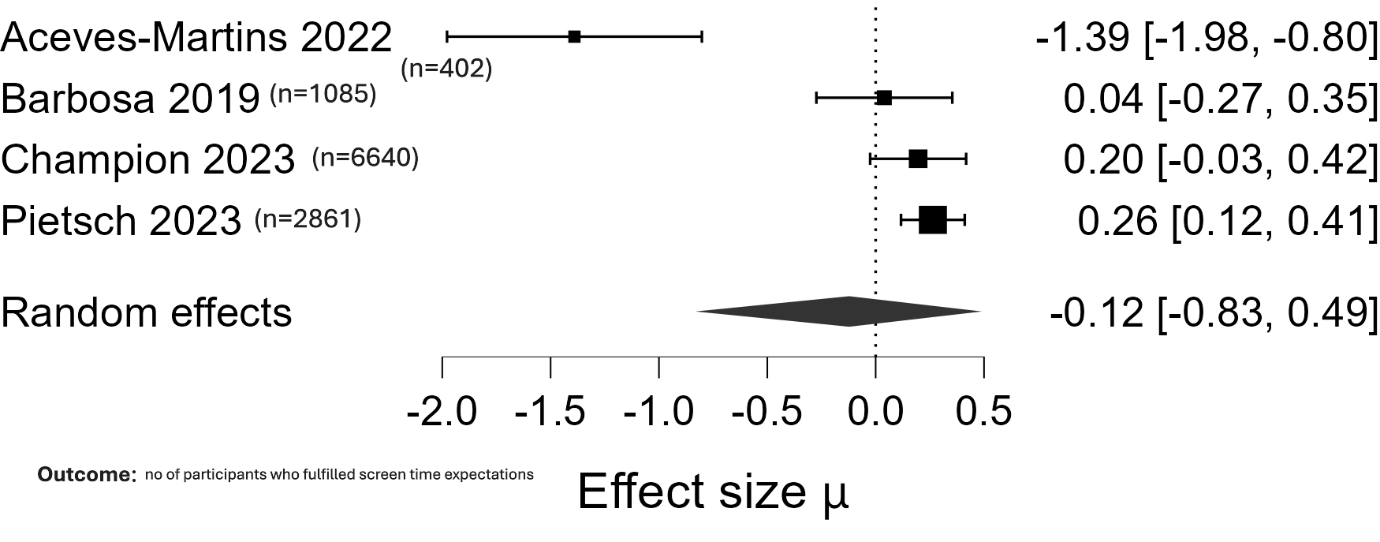


**Figure S3.** funnel plot physical activity.


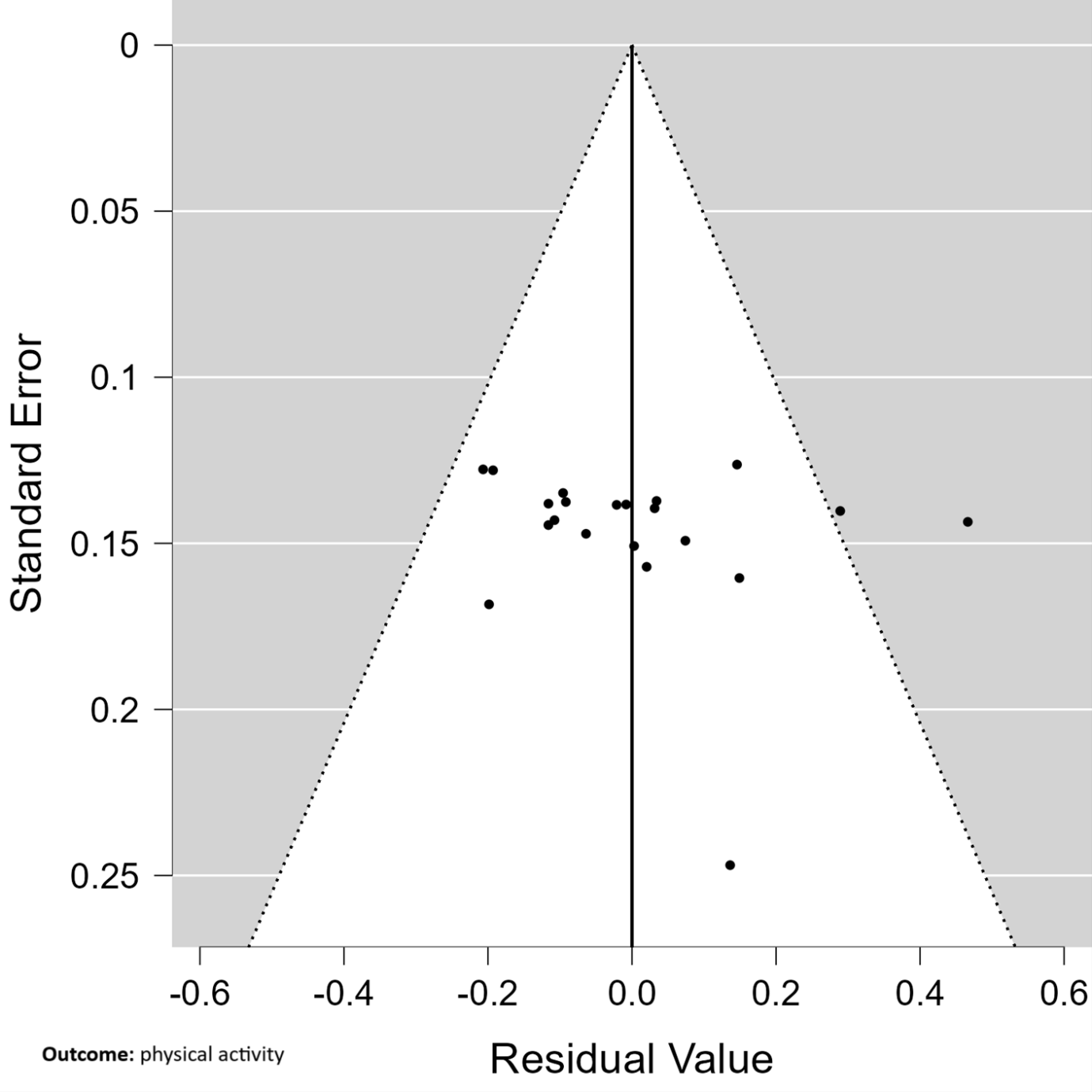


**Figure S4.** forest plot physical activities expectation.


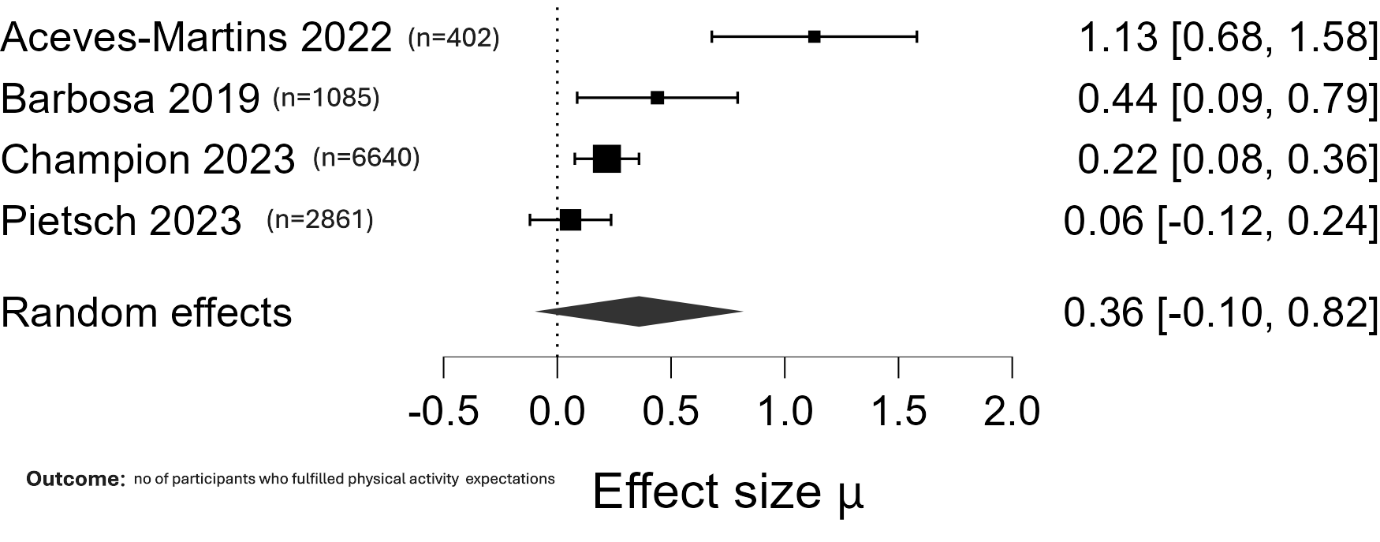


**Figure S5a.** Forest plot BMI overall.


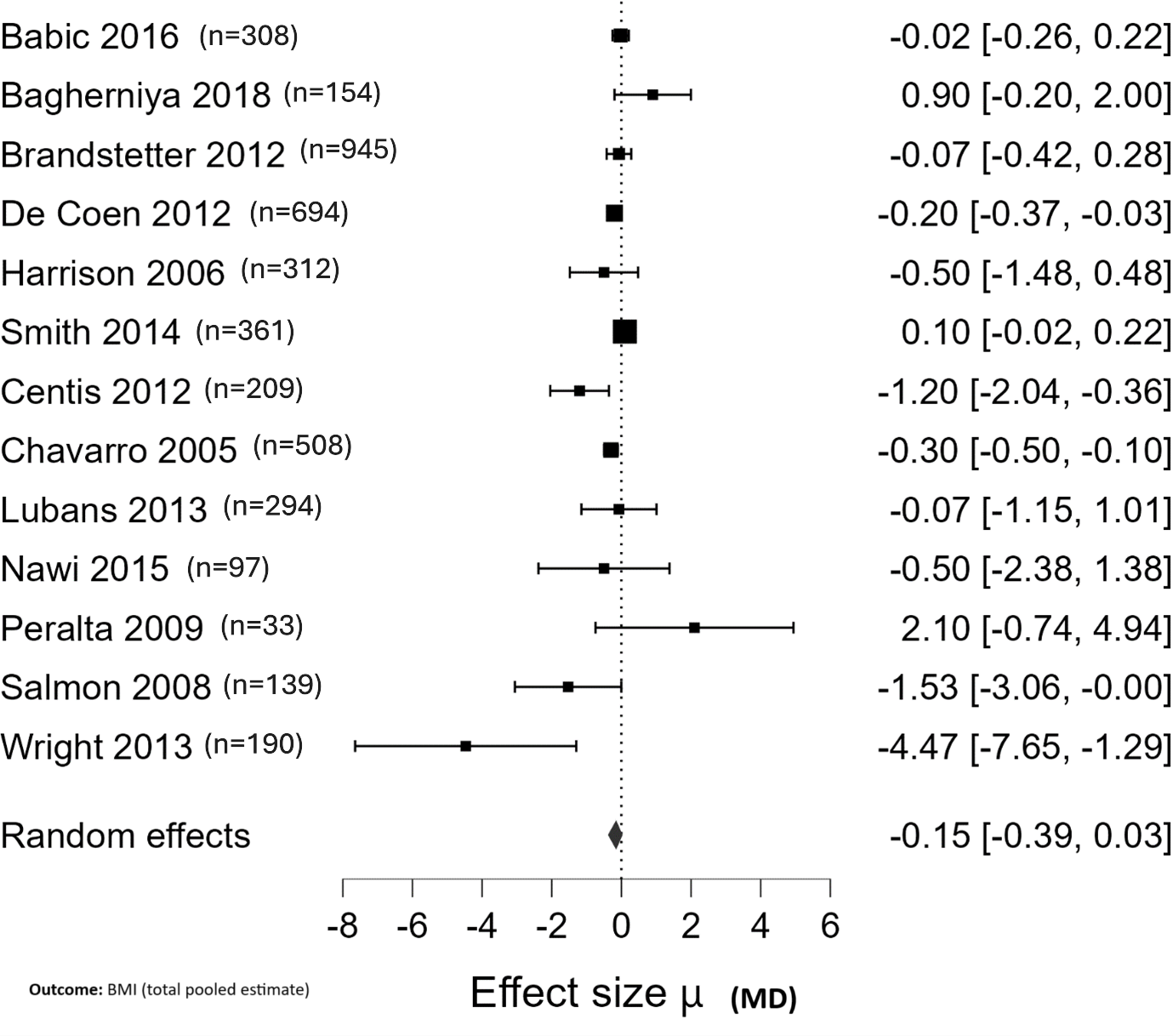


**Figure S5b.** Forest plot BMI screen focused subgroup.


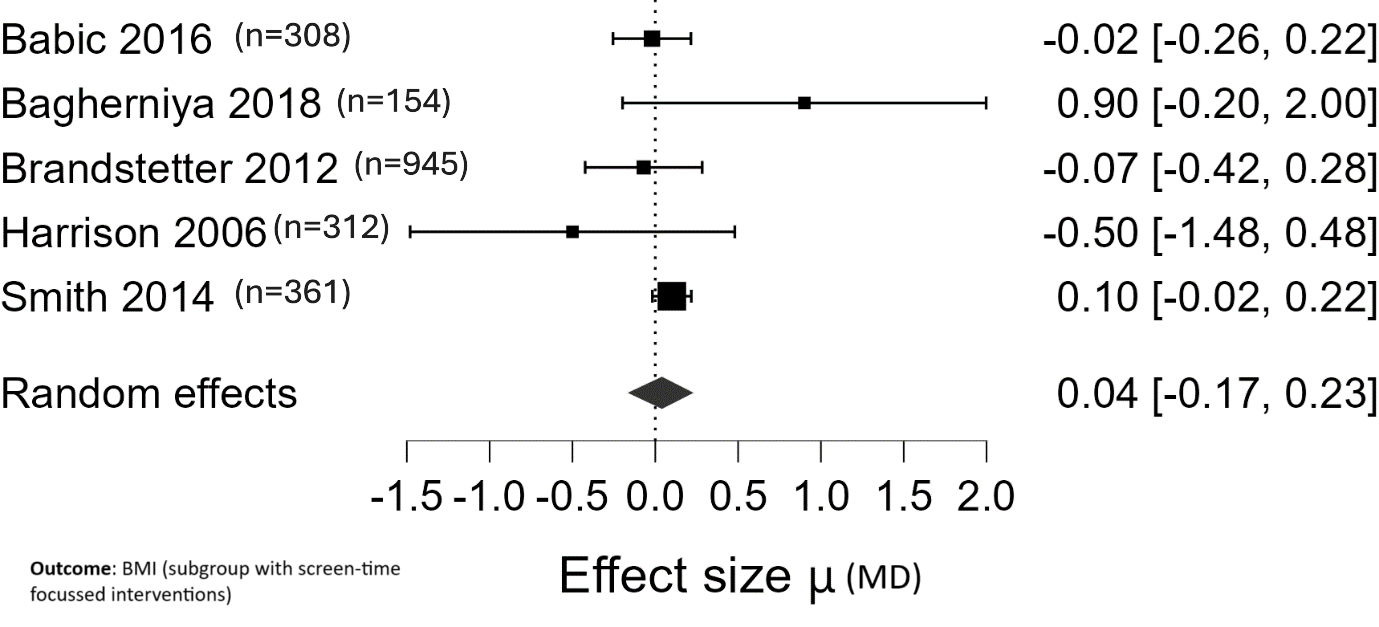


**Figure S5c.** Forest plot BMI lifestyle intervention subgroup


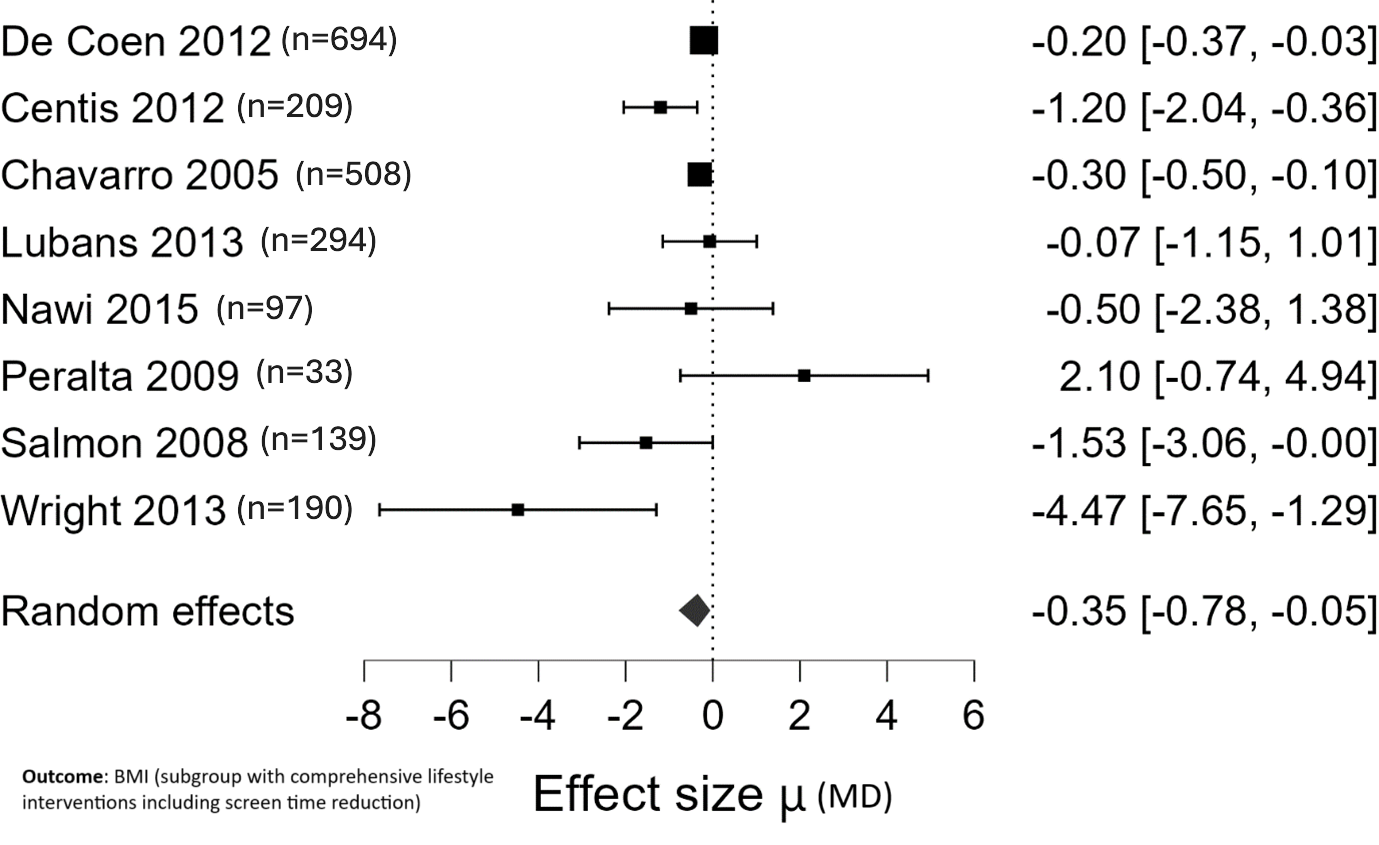


**Figure S6.** funnel plot BMI


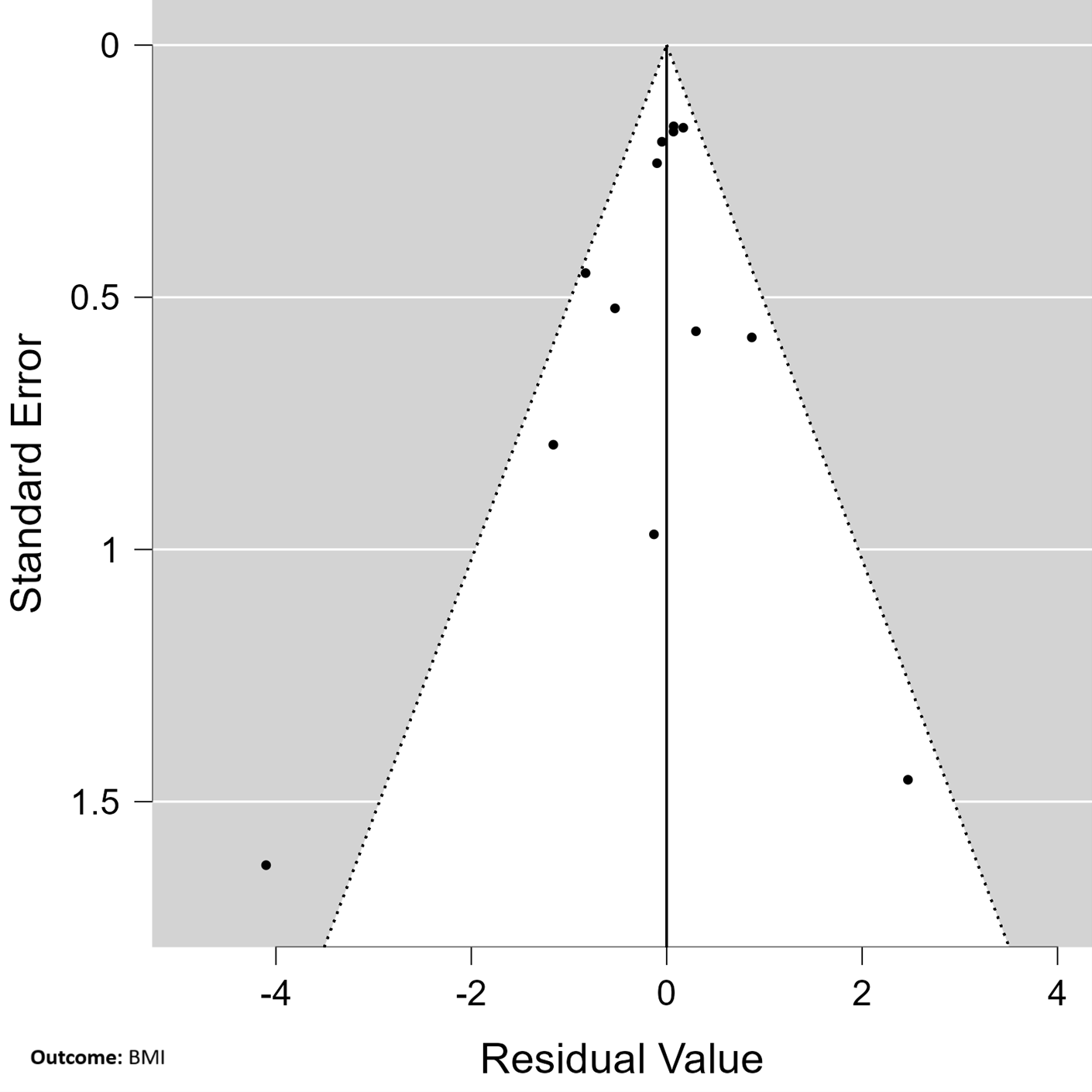


**Figure S7.** forest plot participants with obesity.


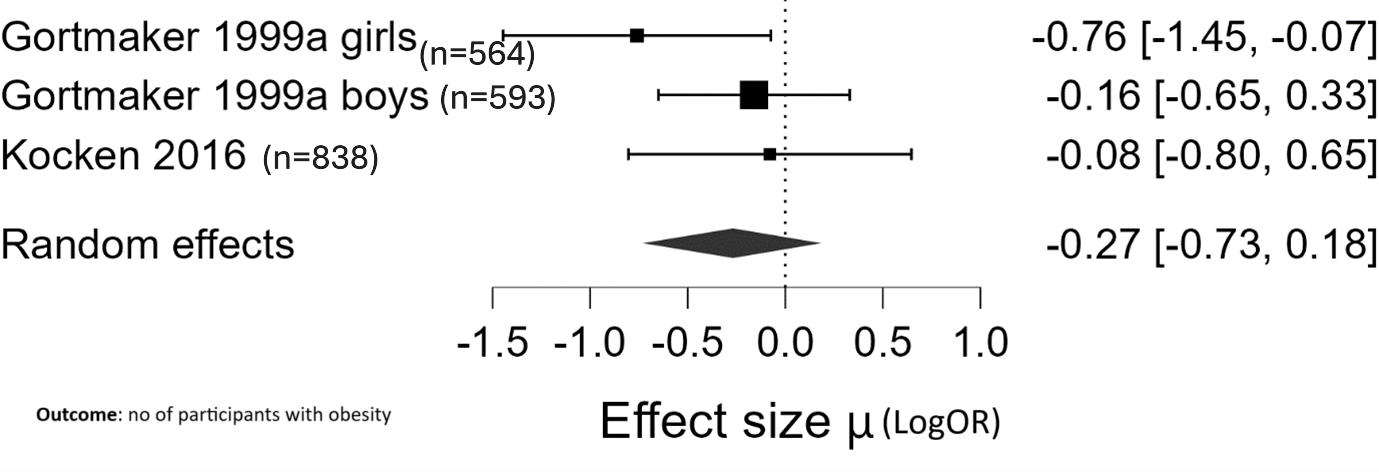


**Figure S8.** forest plot severity internet gaming disorder.


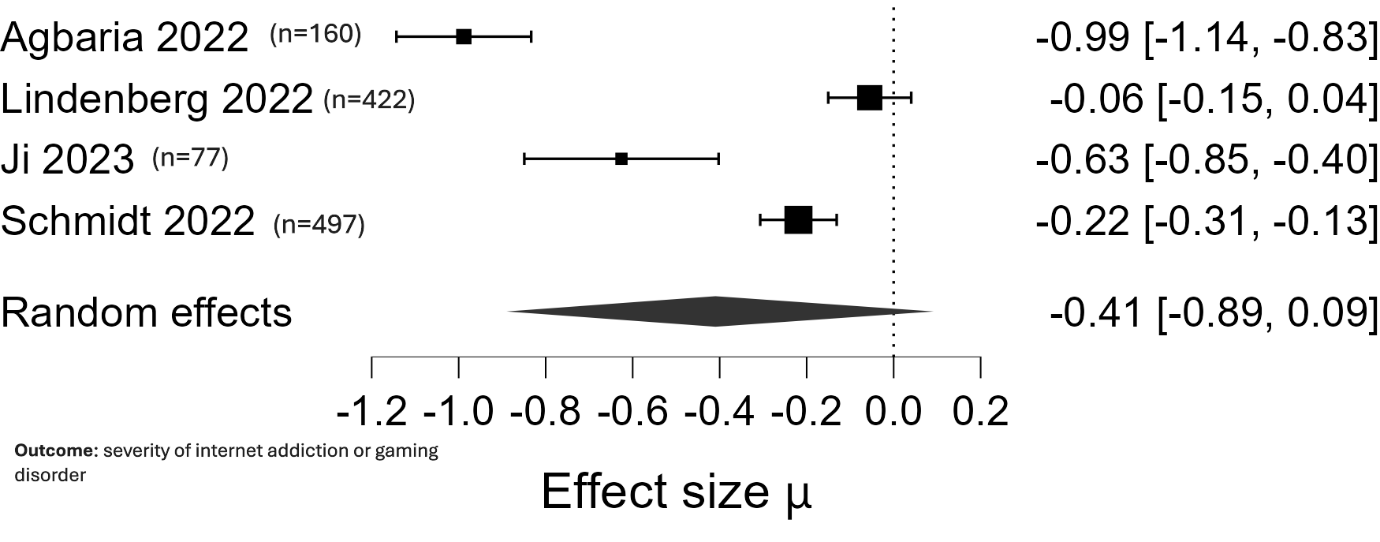


**Figure S9.** forest plot self efficacy well being.


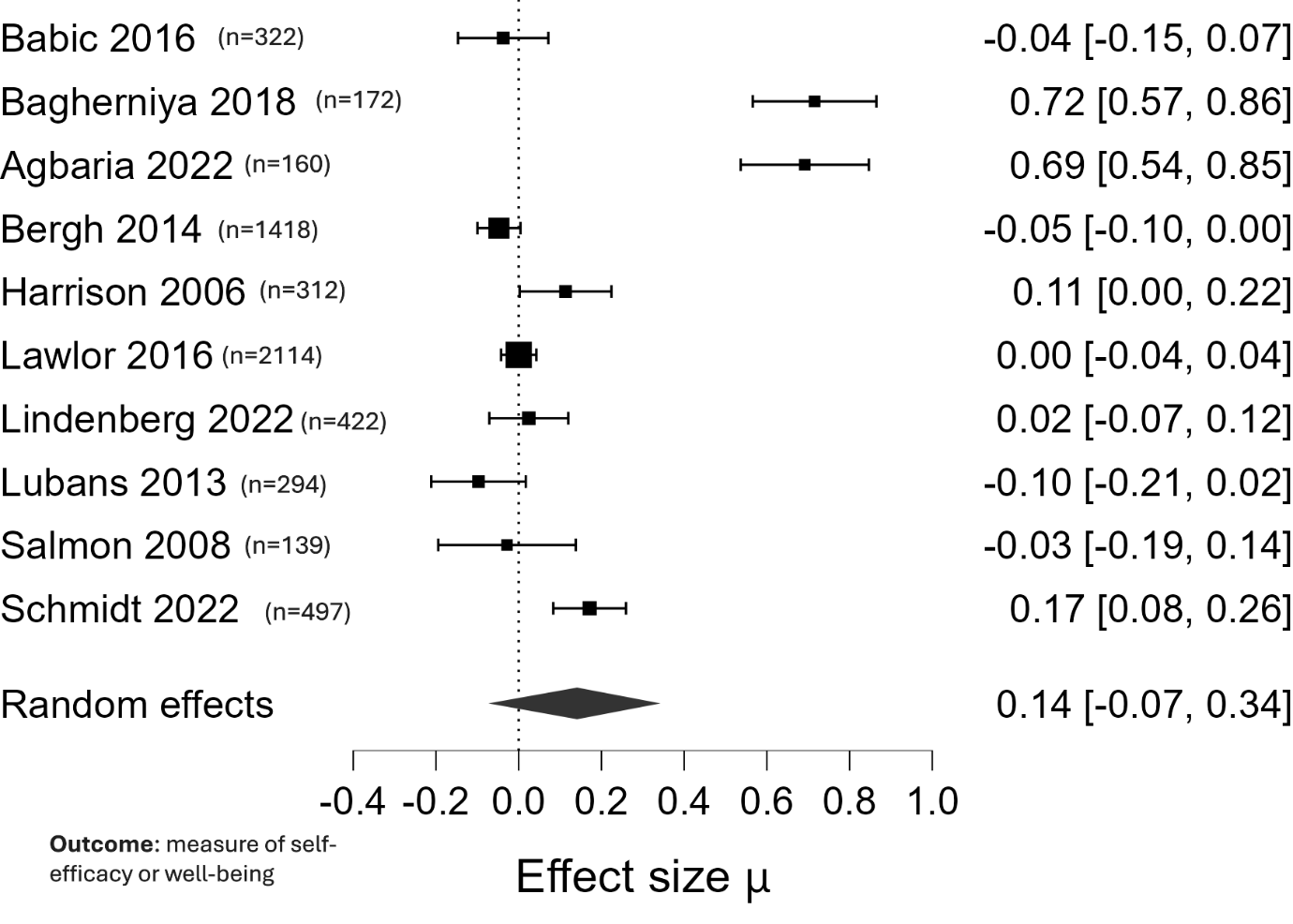

Supplement: Supplementary file 1 — Table S1. Characteristics of included studies. Appendix S1. PRISMA 2020 checklist. Appendix S2. Search strategies. Appendix S3. A detailed description of the review methods. Appendix S4. Citations of shortlisted and excluded studies. Appendix S5. Results: exploration of heterogeneity. Appendix S6. Summary of findings table with ratings of certainty‐of‐evidence. Appendix S7. Citations of published systematic reviews that evaluated interventions to reduce screen time for children. Figure S1. Funnel plot screen time. Figure S2. Forest plot screen time expectation. Figure S3. Funnel plot physical activity. Figure S4. Forest plot physical activities expectation. Figure S5. (a) Forest plot BMI overall. (b) Forest plot BMI screen focused subgroup. (c) Forest plot BMI lifestyle intervention subgroup. Figure S6. Funnel plot BMI. Figure S7. Forest plot participants with obesity. Figure S8. Forest plot severity internet gaming disorder. Figure S9. Forest plot self‐efficacy well‐being. [file CAMH-30-223-s001.docx]
